# The Epileptor Model: A Systematic Mathematical Analysis Linked to the Dynamics of Seizures, Refractory Status Epilepticus, and Depolarization Block

**DOI:** 10.1523/ENEURO.0485-18.2019

**Published:** 2020-03-16

**Authors:** Kenza El Houssaini, Christophe Bernard, Viktor K. Jirsa

**Affiliations:** Aix Marseille University, INSERM, INS, Institut de Neurosciences des Systèmes, 13005 Marseille, France

**Keywords:** bifurcation analysis, depolarization block, dynamical systems theory, epilepsy, neural mass model, refractory status epilepticus

## Abstract

One characteristic of epilepsy is the variety of mechanisms leading to the epileptic state, which are still largely unknown. Refractory status epilepticus (RSE) and depolarization block (DB) are other pathological brain activities linked to epilepsy, whose patterns are different and whose mechanisms remain poorly understood. In epileptogenic network modeling, the Epileptor is a generic phenomenological model that has been recently developed to describe the dynamics of seizures. Here, we performed a detailed qualitative analysis of the Epileptor model based on dynamical systems theory and bifurcation analysis, and investigate the dynamic evolution of “normal” activity toward seizures and to the pathological RSE and DB states. The mechanisms of the transition between states are called bifurcations. Our detailed analysis demonstrates that the generic model undergoes different bifurcation types at seizure offset, when varying some selected parameters. We show that the pathological and normal activities can coexist within the same model under some conditions, and demonstrate that there are many pathways leading to and away from these activities. We here archive systematically all behaviors and dynamic regimes of the Epileptor model to serve as a resource in the development of patient-specific brain network models, and more generally in epilepsy research.

## Significance Statement

Epilepsy is characterized by patient-specific electrophysiological discharges. The range of mechanisms and pathways leading to the same type of seizure, however, is large. The Epileptor model has found many applications in epilepsy research and clinical applications, because it allows the classification and dynamic modeling of seizure types independent of the knowledge of its underlying biophysical mechanisms. It is based purely on the dynamic features of the seizure. We provide here a complete functional atlas of all possible behaviors of the Epileptor model, which serves as a useful resource in modeling brain networks in epilepsy. More, we explore the contribution of the Epileptor model to better understand the dynamics of the refractory status epilepticus and depolarization block phenomena, which are linked to epilepsy.

## Introduction

Epilepsy is a condition of the nervous system in which neuronal populations manifest as repeated epileptic seizures lasting a few minutes. These seizures are spontaneous and commonly accompanied by fast abnormal discharges (10 ms timescale), after which the brain activity slowly returns to normal. Epileptic seizures may present in different forms, as well as transitions from and to these pathological states. Some seizures are controlled by medication, particularly antiepileptic drugs (AEDs). However, there are seizures that last >1 h without returning to baseline, and do not respond to AEDs (31-43%), resulting then in so-called refractory status epilepticus (RSE) (Mayer et al., 2002; Holtkamp et al., 2005; Rossetti et al., 2005). Patients with RSE are at an increased risk of death. Indeed, the short-term mortality rates of RSE are estimated to be between 16% and 39% (Mayer et al., 2002; Holtkamp et al., 2005; Rossetti et al., 2005; Novy et al., 2010).

Other neuroelectric phenomena are linked to epilepsy, among which spreading depression (SD) is the most prominent. SD is characterized by a slowly propagating depolarization wave [or depolarization block (DB)] in neuronal networks, followed by a shutdown of brain activity (Pietrobon and Moskowitz, 2014). DB is a state in which the neuronal membrane is depolarized, but neurons stop firing. Spreading depression may occur during migraine and some seizures (Rogawski, 2008; Pietrobon and Moskowitz, 2014), and was first described by Leo (1944), who observed a depression of electroencephalographic (EEG) activity that moved across the cortex.

The mechanisms underlying the genesis of epilepsy, DB, and RSE are still largely unknown. In this article, we take an integrative approach toward the understanding of these neuroelectric phenomena. Our thinking is inspired by the wish to understand the underlying dynamic underpinnings rather than the biophysiological basis.

In this context, a large number of experimental and computational models have been proposed to clarify the basic mechanisms of seizures and DB. Most computational models rely on biophysically realistic parameters, trying to reproduce experimental data (Kager et al., 2000; Traub et al., 2001; Destexhe, 2008; Cressman et al., 2009; Ullah et al., 2009). Although these models provide important advances in seizures and DB research, they rarely produce general rules. Furthermore, there has been no attempt at evaluating whether or not seizures, RSE, DB, and normal brain activities can coexist, and, if so, under what conditions.

The previously mentioned models are rooted in physiological mechanisms generating a fairly limited range of behaviors. The physiological foundation is critical when the intended therapeutic intervention shall make use of the physiology. Examples include the identification of signaling pathways leading to the control of neurotransmitters linked to excitability such as lamotrigine or topiramate acting on calcium channels. Another type of intervention acts on the brain as a network and harnesses the capacity to modulate networks, such as stimulation, resection, or disconnection. In this case, physiological realism of a mechanism is to be replaced by dynamic realism as the network communication depends more on the type of signal rather than how it is generated. These types of models are phenomenological. A neural mass model of partial seizures called Epileptor was previously developed, which has found many applications in brain network modeling of epilepsy patients and is the network node model used in the European clinical trial EPINOV (www.epinov.com; Jirsa et al., 2014). Given the wide application of this model and its relevance for clinical research, we here archive in detail its dynamic repertoire. The Epileptor comprises one susbsystem (called subsystem 1) with two state variables responsible for generating fast discharges, another subsystem (called subsystem 2) with two state variables generating sharp-wave events (SWEs). The subsystem 1 (fast) and subsystem 2 (slow) are linked to a state variable, *z*, evolving on a very slow timescale called the permittivity variable (Jirsa et al., 2014). Interestingly, the transition from and to the pathologic states can occur autonomously, under the slow *z* evolution. Fast discharges and sharp-wave events are pathological features commonly associated with seizures despite their different forms. The goal of this article is, first, to perform a systematic mathematical analysis of the Epileptor; and, second, to determine the range of behaviors present in the Epileptor, with the further reaching goal to ask whether “normal” brain activities, seizures, RSE, and DB can coexist within the same model. To this aim, we present a qualitative analysis of the Epileptor model based on dynamical systems theory and bifurcation analysis.

## Materials and Methods

We provide here a detailed bifurcation analysis of the Epileptor model, which is a neural mass model of partial seizures, to analyze seizure dynamics (Jirsa et al., 2014). The Epileptor model consists of a system of coupled nonlinear differential equations with five state variables. It comprises two 2D subsystems and one slow variable *z*. Subsystems 1 and 2 are responsible for fast discharges and SWEs, respectively (Jirsa et al., 2014). Analysis of the separated subsystems 1 and 2 was performed to provide more in-depth information on the Epileptor dynamics.

### Analysis of the Epileptor

#### Epileptor equations

The Epileptor equations generate seizure-like events (SLEs) written in the following form:

(1) $${\dot{x}}_{1}=y_{1}-f_{1}(x_{1},x_{2})-z+I_{ext_{1}}$$

(2) $${\dot{y}}_{1}=c_{1}-d_{1}x_{1}^{2}-y_{1}\;$$

(3) $$\dot{z}=\{\begin{array}{ll} r(s(x_{1}-x_{0})-z-0.1z^{7}) & \;\text{if}\;\text{z}<\text{0}\\ r(s(x_{1}-x_{0})-z) & \;\text{if}\;\text{z}\geq \text{0} \end{array}\;$$

(4) $${\dot{x}}_{2}=-y_{2}+x_{2}-x_{2}^{3}+I_{ext_{2}}+0.002g-0.3(z-3.5)\;$$

(5) $${\dot{y}}_{2}=(-y_{2}+f_{2}(x_{2}))/\tau_{2}\;$$

where

(6) $$f_{1}(x_{1},x_{2})=\{\begin{array}{ll} ax_{1}^{3}-bx_{1}^{2} & \;\text{if}\;x_{1}<0\\ -(m-x_{2}+0.6{\left(z-4\right)}^{2})x_{1} & \;\text{if}\;x_{1}\geq 0 \end{array}$$

(7) $$f_{2}(x_{2})=\{\begin{array}{ll} 0 & \;\text{if}\;x_{2}<-0.25\\ a_{2}(x_{2}+0.25) & \;\text{if}\;x_{2}\geq -0.25 \end{array}$$

(8) $$g(x_{1})=\int_{t_{0}}^{t}e^{-\gamma (t-\tau )}x_{1}(\tau )\;\text{d}\tau$$

In what follows, unless otherwise stated, the values of the parameters are as follows:

Subsystem 1: *a* = 1; *b* = 3; *c *= 1; *d *=* *5; $I_{ext_{1}}=3.1$; *m *=* * 0

Subsystem 2: $a_{2}=6$; $\tau_{2}=10$; $I_{ext_{2}}=0.45$; *γ* = 0.01

Slow *z* dynamics: *r * = * *0.00,035; *s * = * *4; *x*_0_ = −1.6.

Equations 1 and 2 describe the subsystem 1 dynamics and equations; Equations 4 and 5 describe subsystem 2. The state variables *x*_1_ and *y*_1_ comprise the subsystem 1 responsible for fast discharges, and *x*_2_ and *y*_2_ the subsystem 2 involved in spike-wave events (Jirsa et al., 2014). The third differential equation represents a slow adaptation variable. The Epileptor model evolves under a slow timescale, *r* (small). Equations 1–3 represent a fast-slow subsystem.

#### Numerical integration

All stochastic simulations are performed with linear additive Gaussian white noise with a zero mean and a variance of 0.0025 using the Euler–Maruyama method. Deterministic simulations are performed using the Runge-Kutta method with a maximal time step of 0.01.

#### Finding equilibrium points and their stability

In order to understand the Epileptor dynamics, we identify the equilibrium points and investigate their stability. This analysis is performed on the deterministic Epileptor model. We find the equilibrium points $E(x_{1}^{*},y_{1}^{*},z^{*},x_{2}^{*},y_{2}^{*})$ by solving ${\dot{x}}_{1}\;=\;{\dot{y}}_{1}\;=\;\dot{z}\;=\;{\dot{x}}_{2}\;=\;{\dot{y}}_{2}\;=\;0$. We obtain a system of algebraic equations that we solve using the Matlab “solve” symbolic solver. The variables to solve for are the state variables of the Epileptor model, which are $x_{1},y_{1},z,x_{2},y_{2}$. The inputs of the solve function are the parameter values of the Epileptor model. Here, we analyze the existence of the equilibrium points when varying the *m* and *x*_0_ parameters. The solutions are the vector equilibrium points. We first determine the equilibrium points of the whole system when the parameter *x*_0_ varies, for two values of the parameter *m* (*m * = * *0 and *m * = * *0.5). We determine the stability of the equilibrium points *E* by evaluating the eigenvalues of the Jacobian matrix *J* at the equilibrium point *E*, which is stable if all the real parts of the eigenvalues of *J* are negative (Izhikevich, 2007).

### Parameter space of equilibrium points

We explore the stability of the coexisting equilibrium points for a range of *m* and *x*_0_ values. For each values combination, we determine the equilibrium points using the solve function and their stability using the Jacobian matrix. The complete results are drawn within a (*m*, *x*_0_) parameter space for equilibrium points, which consists of various areas, each of which is characterized by coexisting equilibrium points with different stability. Moreover, when the parameter $I_{ext_{2}}$ is varied, the Epileptor behavior changes. We therefore explore two parameter spaces of equilibrium points, for $I_{ext_{2}}=0.45$ (default Epileptor value) and $I_{ext_{2}}=0$.

#### Bifurcation diagram

**Figure 1.**
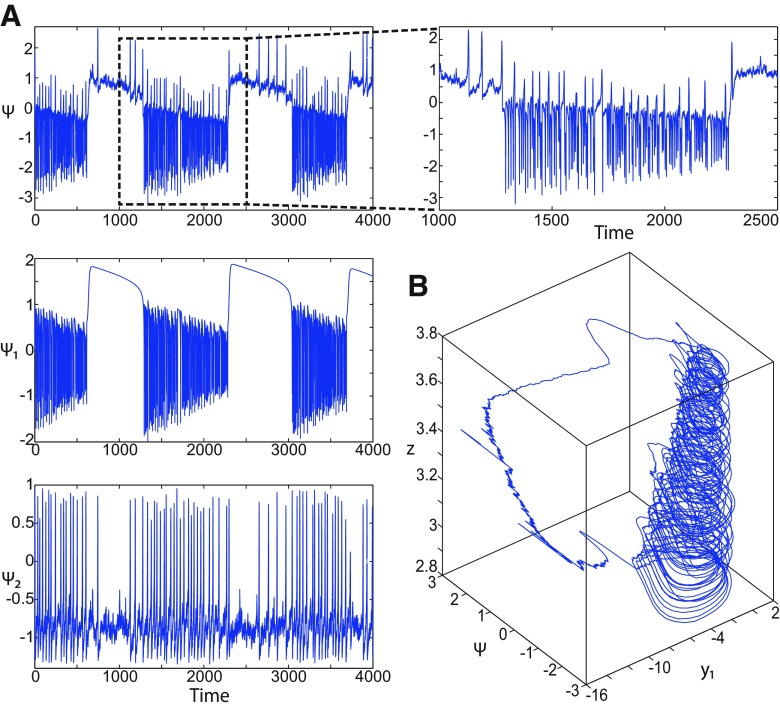
On the seizures dynamics. ***A***, Time series of the Epileptor model (its enlarged view is shown on the right), the first (middle), and second (bottom) subsystem are plotted showing the principal components of a seizure-like event, that is an interictal period with no spikes, emergence of preictal spikes, ictal onset, seizure evolution, and emergence of sharp-wave events toward ictal offset. *ψ*, *ψ*_1_, and *ψ*_2_ correspond to $-x_{1}+x_{2}$, *x*_1_, and *x*_2_ respectively. ***B***, The trajectory of the whole system is sketched in the (*y*_1_, *ψ*, *z*) phase space. Seizure offset and ictal onset emerge through the *z* evolution. Here all stochastic simulations were performed with Gaussian white noise using the Euler–Mayurama method. Main parameters values: *m * = * *0, *x*_0_ = −1.6, and *r * = * *0.00035. Initial conditions are [0 −5 3 0 0 0.01].

**Figure 2.**
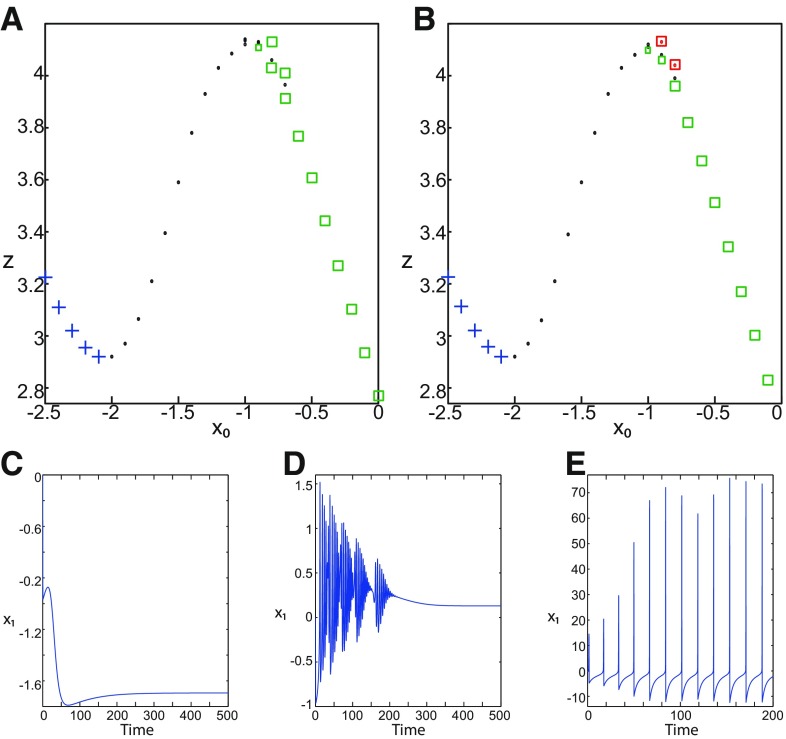
***A***, ***B***, Equilibrium points of the Epileptor model with respect to *x*_0_ for *m * = * *0.5 (*A*) and *m * = * *0 (*B*). *z* is the third coordinate of the vector equilibrium points. Stable nodes and saddles are labeled as blue plus sign markers and black dots, respectively. Stable and unstable foci are labeled as red squares-dotted and green squares, respectively. ***C–E***, Time series (stochastic) of the Epileptor model exhibit a normal activity (***C***), a nonoscillatory state (***D***), and a periodic solution (***E***). The parameters *m* and *x*_0_ are: *m* = 0 and *x*_0_ = −2.5 (***C***), *m*  =  0 and *x*_0_ = −0.9 (***D***), and *m *=* *0.5 and *x*_0_ = −0.9 (***E***).

One important step in the analysis of a mathematical model is the geometrical analysis of bifurcations, which here was performed on the deterministic Epileptor model. First, we draw a (*z*, *x*_1_) bifurcation diagram of the Epileptor for constant *m*, here *m * = * *0.5. The (*z*, *x*_1_) bifurcation diagram comprises the equilibrium points of the Epileptor, where *x*_1_ is the first coordinate of the vector of equilibrium points that we determined with respect to *z*. All equilibrium point solutions were obtained using the Matlab solve symbolic solver, where only the (${\dot{x}}_{1},\;{\dot{y}}_{1},\;{\dot{x}}_{2},\;{\dot{y}}_{2}$) equations were considered for constant *z*. We discretized the space for 801 points in the *z*-dimension and performed numerical continuation computing the linear stability using the Jacobian matrix. The (*z*, *x*_1_) curve is divided into different branches, of which each is numerically continued separately representing a different stability type of the equilibrium points solutions. Bifurcations were identified at stability changes for given *z*-values and are indicated by dots in the corresponding visualizations. As the main parameters *m* and $I_{ext_{2}}$ control the dynamics of the Epileptor, we typically present bifurcation diagrams for varying values of *m* and $I_{ext_{2}}$. All bifurcation diagrams were verified using XPPAUT and explicit numerical simulations of the model system (typically 2000–4000 units of time, of which the initial transients are removed (Figs. 1, 2), for which representative trajectories were plotted in the corresponding diagrams.

#### Finding a fast-slow limit cycle

We found a large attractor in the (*y*_1_, *ψ*, *z*) phase space below the SLE attractor shown in Figure 1 The *z* equation of the Epileptor model (Jirsa et al., 2014) was introduced as follows:

(9) $$\dot{z}=r(s(x_{1}-x_{0})-z),\forall z.$$

When using this *z* equation, the system evolves toward the attractor and then diverges with time, which we show in a (*z*, *x*_1_) bifurcation diagram. Thus, we modify the *z* equation to stabilize the final state. We interpret graphically the divergence of the Epileptor by introducing the averaging method, which is used to locate the periodic orbits in the phase space. The periodic orbits are intersections of the *z*-nullcline and the $<x_{1}>$-curve. The *z*-nullcline is given by $\dot{z}=0$, and the $<x_{1}>$-curve is the average value of *x*_1_ for each *z* constant written in the following form:

(10) $$<x_{1}(z)>=\frac{1}{T(z)}\int_{0}^{T(z)}\phi (t;z)\;\text{d}t,$$

where

(11) $$x_{1}=\phi (t;z).$$

We also determine the periodic orbits by using the Pontryagin’s averaging technique (Shilnikov and Kolomiets, 2008). To illustrate this technique, we introduce a slow averaged nullcline written as follows:

(12) $$<\dot{z}>=\{\begin{array}{ll} r(s(<x_{1}>-x_{0})-z-0.1z^{7}) & \text{if}\;z\;<0\\ r(s(<x_{1}>-x_{0})-z) & \text{if}\;z\geq 0 \end{array}=0$$

where periodic orbits are the zeros of the $<\dot{z}>$. We determine the stability of periodic orbits using the following derivative:

(13) $${\frac{d<\dot{z}>}{dz}|}_{z=z^{*}},$$

which represents the dynamics of the averaged equation. A periodic orbit is stable when Equation 13 is negative, and graphically if the graph of $<\dot{z}>$ decreases at the given zero. First, we find the periodic orbits as the parameter *x*_0_ varies in a (*x*_0_, *z*) bifurcation diagram of periodic orbits. We determine the bifurcation that results in the appearance or disappearance of the periodic orbits. Second, we explore two parameter spaces of periodic orbits for $I_{ext_{2}}=0.45$ and $I_{ext_{2}}=0$.

#### Stabilizing equilibrium points in the Epileptor model

The parameter *x*_0_ can change the stability of equilibrium points and then can control the Epileptor behavior. Using the fact that a trajectory moves around the equilibrium point when it is stable, we use the bifurcation analysis to find the values of *x*_0_ for which an equilibrium point is stable. There is a qualitative distinction between the stable equilibrium points, which we discuss in more detail later.

#### Coexisting attractors in the Epileptor model

The Epileptor behavior depends on the stability of its equilibrium points. More, there is a pre-existing limit cycle (LC) attractor, which is a stable periodic orbit. In this case, the trajectory can have a behavior that is controlled by the stability of the equilibrium points or can jump to the limit cycle attractor. Thus, there is a coexistence of two attractors. We identify all coexisting attractors according to the values of the parameters *m* and *x*_0_.

### Analysis of subsystem 1

We provide a detailed analysis of the subsystem 1 dynamics without coupling (i.e., subsystem 1 is not coupled to subsystem 2).

#### Subsystem 1 equations

The equations of subsystem 1 are given by:

(14) $${\dot{x}}_{1}=y_{1}-f_{1}(x_{1},x_{2})-z+I_{ext_{1}}$$

(15) $${\dot{y}}_{1}=c_{1}-d_{1}x_{1}^{2}-y_{1}\;$$

where

(16) $$f_{1}(x_{1},x_{2})=\{\begin{array}{ll} ax_{1}^{3}-bx_{1}^{2} & \;\text{if}\;{\text{x}}_{\text{1}}<\text{0}\\ -(m+0.6{\left(z-4\right)}^{2})x_{1} & \;\text{if}\;{\text{x}}_{\text{1}}\geq \text{0} \end{array}$$

*z* is constant (r→0).

#### Subsystem 1 equilibrium points and stability

We analytically find the equilibrium points (*x*_1_, *y*_1_) by solving the following equations:

(17) $$\{\begin{array}{l} {\dot{x}}_{1}=y_{1}-f_{1}(x_{1},x_{2})-z+I_{ext_{1}}=0\\ {\dot{y}}_{1}=c_{1}-d_{1}x_{1}^{2}-y_{1}=0.\; \end{array}$$

We determine the stability of the equilibrium points by evaluating the eigenvalues of the Jacobian matrix *J*. An equilibrium point is stable if all the real parts of the eigenvalues of *J* are negative (Izhikevich, 2007).

We graphically find the equilibrium points by intersecting the *x*_1_- and *y*_1_-nullclines. We determine the nullclines of subsystem 1 and show how to find the equilibrium points and their stability in a phase plane.

#### Subsystem 1 bifurcation diagram

When finding the equilibrium points of the subsystem 1, we observe a qualitative change of the phase plane, which is interpreted as a bifurcation. We identify the different types of bifurcations that exist according to *z*, which is considered as a parameter control, and the parameter *m*.

Using the bifurcation diagrams, we analyze these bifurcations and the qualitative behavior of the susbsystem 1 with respect to *z*. The bifurcation analysis is performed on the deterministic subsystem 1. First, we plot the whole bifurcation diagram of the subsystem 1 for *m * = * *0, which consists of two curves. The curves consist of the subsystem 1 equilibrium points for each value of *z*. When the parameter *m* changes, the shape of one of the curves changes on an interval of *z*. As a result, we then plot only this curve in bifurcation diagrams for *m * = * *0 and *m * = * *2, and describe the trajectories behavior for each value of *m*. Below, we summarize the types of bifurcations that exist with respect to *m*.

#### Fast-slow subsystem equations

The equilibrium points of subsystem 1 depend on *z*. When *m * = * *0 and *z * = * *3.1, three equilibrium points coexist: a saddle, a stable node and a stable focus (see *m * = * *0; see Fig. 39*A*). When *m * = * *1.5 and *z * = * *3.1, then equilibrium points coexist: a saddle, a stable node, and an unstable focus (see *m * = * *1.5; see Fig. 39*A*). A stable node and a stable focus are equilibrium points of a resting state and a nonoscillatory state, respectively. When the resting and nonoscillatory states coexist, the trajectories converge to one of them, depending on the initial conditions (see *m * = * *0 and *m * = * *1; see Fig. 39*A*).

To ensure that trajectories switch between two stable states, we introduce the following equation:

(18) $$<\dot{z}>=\{\begin{array}{ll} r(s(x_{1}-x_{0})-z-0.1z^{7}) & \;\text{if}\;\text{z}<\text{0}\\ r(s(x_{1}-x_{0})-z) & \;\text{if}\;\text{z}\geq \text{0} \end{array}=0$$

to the subsystem 1

(19) $${\dot{x}}_{1}=y_{1}-f_{1}(x_{1},x_{2})-z+I_{ext_{1}}\;$$

(20) $${\dot{y}}_{1}=c_{1}-d_{1}x_{1}^{2}-y_{1}.\;$$

*r* and *s* are positive constant parameters (r<<1).*z* changes the input $I_{ext_{1}}$ of the subsystem 1 to $-z+I_{ext_{1}}$. Indeed, when *z* decreases, then $-z+I_{ext_{1}}$ is increased, and only a stable focus exists (see Fig. 39*B*). If *m* increases, the stable focus becomes unstable, surrounded by a stable limit cycle. Hence, only a stable limit cycle exists (see Fig. 39*B*). When *z* increases, then $-z+I_{ext_{1}}$ is reduced, and the stable limit cycle coexists with a stable node (see Fig. 39*A*). The saddle acts as a separatrix (*S*) between them. When *z* further increases, the stable limit cycle disappears through a homoclinic bifurcation, HB (see Fig. 39*C*), and hence only a stable node exists. Thus, *z* mimics a slow adaptation of the subsystem 1 to produce resting and oscillatory states, and ways to switch between them. The dynamics of subsystem 1 is fast and *z* is a slow variable, hence Equations 18–20 represent a fast-slow subsystem.

#### Finding equilibrium points of the fast-slow subsystem

We analytically find the equilibrium points by solving Equations 19 and 20, where *z* is a solution of $\dot{z}=0$ (Eq. 18). We determine the stability of the equilibrium points by analyzing the Jacobian matrix *J*. The equilibrium point is stable if all the real parts of the eigenvalues of *J* are negative (Izhikevich, 2007). We graphically find the equilibrium points of the fast-slow subsystem by using the (*z*, *x*_1_) bifurcation diagram and the *z*-nullcline, which is related to the parameter *x*_0_. We show how the *z*-nullcline moves in the bifurcation diagram when varying *x*_0_, and use the bifurcation diagram to find the equilibrium points of the fast-slow subsystem for each value of *x*_0_.

#### Finding periodic orbits of the fast-slow subsystem

As follows from the Epileptor analysis, we determine the periodic orbits of the fast-slow subsystem as well as their stability by using the averaging method and the Pontryagin’s averaging technique. The slow averaged nullcline is given by the following:

(21) $$<\dot{z}>=\frac{r}{T(z)}\int_{0}^{T(z)}R(z,\phi (t;z))\;\text{d}t=0,$$

where

(22) $$R(z,x_{1})=\{\begin{array}{ll} s(x_{1}-x_{0})-z-0.1z^{7} & \;\text{if}\;\text{z}<\text{0}\\ s(x_{1}-x_{0})-z & \;\text{if}\;\text{z}\geq \text{0}. \end{array}$$

$x_{1}=\phi (t;z))$ is a solution of the subsystem 1 with *z* constant.

Here, we plot the graph of $<\dot{z}>$ to explain how we find the periodic orbits and their stability. The periodic orbits are the zeros of the graph of $<\dot{z}>$. It is stable if the graph of $<\dot{z}>$ decreases at the given zero. More, we find the periodic orbits as *x*_0_ varies and determine the bifurcation leading to their appearance or disappearance.

#### Stabilizing equilibrium points in the fast-slow subsystem

Our approach to stabilizing the equilibrium points of the fast-slow subsystem is the same as that of the Epileptor model: analyze bifurcation diagrams of the fast-slow subsystem and then find the values of *x*_0_ for which an equilibrium point is stable. We show how stable equilibrium points can correspond to different states.

#### Coexisting attractors in the fast-slow subsystem

The fast-slow subsystem exhibits a bistability of two attractors as the Epileptor model. The first attractor is the fast-slow limit cycle. The behavior of the second attractor depends on the parameters *m* and *x*_0_, which control the stability of the equilibrium point. We discuss the coexisting attractors when *m* and *x*_0_ have different values.

### Analysis of subsystem 2

In this section, we analyze the dynamics of subsystem 2 (without coupling), which generates SWEs. We explore the equilibrium points, evaluating their stability and determining the bifurcation types.

#### Subsystem 2 equations

The subsystem 2 equations are given by the following:

(23) $${\dot{x}}_{2}=-y_{2}+x_{2}-x_{2}^{3}+I_{ext_{2}}$$

(24) $${\dot{y}}_{2}=(-y_{2}+f_{2}(x_{2}))/\tau_{2}\;$$

where

(25) $$f_{2}(x_{2})=\{\begin{array}{ll} 0 & \;\text{if}\;{\text{x}}_{\text{2}}<\text{-0}.\text{25}\\ a_{2}(x_{2}+0.25) & \;\text{if}\;x_{2}\geq -0.25 \end{array}.$$

#### Subsystem 2 equilibrium points and stability

We analytically find the equilibrium points (*x*_2_, *y*_2_) by solving the following equations:

(26) $${\dot{x}}_{2}=-y_{2}+x_{2}-x_{2}^{3}+I_{ext_{2}}=0$$

(27) $${\dot{y}}_{2}=(-y_{2}+f_{2}(x_{2}))/\tau_{2}=0\;$$

We determine the stability of the equilibrium points by evaluating the eigenvalues of the Jacobian matrix *J*_2_. *J*_2_ is defined at the equilibrium point *x*_2_, which is stable if all the real parts of the eigenvalues of *J*_2_ are negative (Izhikevich, 2007).

The equilibrium points lie graphically at the intersection of the *x*_2_- and *y*_2_-nullclines. We determine the nullclines of the subsystem 2 and show how the equilibrium points and their stability changes as the parameter $I_{ext_{2}}$ varies.

#### Subsystem 2 bifurcation diagram

As the phase plane changes qualitatively when varying the parameter $I_{ext_{2}}$, we discuss this change in a bifurcation diagram where $I_{ext_{2}}$ is the parameter control. The analysis is performed on the deterministic subsystem 2.

## Results

To get a better understanding of the dynamics of the generation and termination of epileptic seizures, we adopted a computational model that reproduces epileptic activity and perform a detailed analysis using a mathematical approach.

### The Epileptor

#### Epileptor dynamics

##### Epileptor model behavior

The Epileptor equations generate SLEs, which are characterized by an onset and offset (Jirsa et al., 2014). We plot time series of the Epileptor system *ψ*, subsystem 1 *ψ*_1_, and subsystem 2 *ψ*_2_ in Figure 1*A*. We find the major elements of an SLE: onset, timescale, offset of SLEs, and their recurrence. Figure 1*A* shows that during the ictal phase, fast discharges decrease in frequency with time. Ictal states are separated by a period of normal brain activity (non-ictal state). State variables *x*_1_ and *y*_1_ are responsible for generating fast discharges in the ictal state with a fast timescale (Fig. 1*A*; see *ψ*_1_). State variables *x*_2_ and *y*_2_ are responsible for generating the SWEs with an intermediate timescale (Fig. 1*A*; see *ψ*_2_). We plot the Epileptor trajectory in a (*y*_1_, *ψ*, *z*) phase space (Fig. 1*B*). Ictal and normal states (NSs) coexist and their coexistence necessitates a separation in the state space so-called “separatrix.” The ictal onset occurs when the trajectory collides with the separatrix after a transient normal state. The seizure offset occurs when the trajectory collides with the separatrix after a transient ictal state. The separatrix acts as a barrier between ictal and normal states. The slow state variable *z* is responsible for the alternation of both states under a slow timescale (r<<1).

##### Equilibrium points

Using the Matlab solve symbolic solver, we localize the equilibrium points *E* as *x*_0_ varies in a (*x*_0_, z*) diagram for *m * = * *0.5 (Fig. 2*A*) and *m * = * *0 (Fig. 2*B*). z^*^ is the third component of the equilibrium points *E*. For *m * = * *0.5 and *m * = * *0, a unique equilibrium point exists when *x*_0_ = −1.6, while three equilibrium points coexist when *x*_0_ = −0.9 (Fig. 2*A*,*B*). The Jacobian matrix *J* of the Epileptor at the equilibrium point E is given by the following:

$$J=\left|\begin{array}{ccccc} A & 1 & Z_{1} & B & 0\\ -10x_{1} & -1 & 0 & 0 & 0\\ rs & 0 & Z_{2} & 0 & 0\\ 0 & 0 & -0.3 & 1-3x_{2}^{2} & -1\\ 0 & 0 & 0 & C & -1/\tau \end{array}\right|$$

where

(28) $$A=\{\begin{array}{ll} -3x_{1}^{2}+6x_{1} & \;\text{if}\;x_{1}<0\\ m-x_{2}+0.6{\left(z-4\right)}^{2} & \;\text{if}\;x_{1}\geq 0 \end{array}$$

(29) $$B=\{\begin{array}{ll} 0 & \;\text{if}\;x_{1}<0\\ -x_{1} & \;\text{if}\;x_{1}\geq 0 \end{array}$$

(30) $$C=\{\begin{array}{ll} 0 & \text{if}\;x_{2}<-0.25\\ a_{2}/\tau & \text{if}\;x_{2}\geq -0.25 \end{array}$$

(31) $$Z_{1}=\{\begin{array}{ll} -1 & \;\text{if}\;x_{1}<0\\ 1.2(z-4)x_{1}-1 & \;\text{if}\;x_{1}\geq 0 \end{array}$$

(32) $$Z_{2}=\{\begin{array}{ll} r(-1-0.7z^{6}) & \;\text{if}\;z<0\\ -r & \;\text{if}\;z\geq 0 \end{array}.$$

The stability of the Epileptor equilibrium points depends on *x*_0_ (Fig. 2*A*,*B*). For *x*_0_ = −1.6 and *m * = * *0 or *m * = * *0.5, the equilibrium point is a saddle. The stable manifold of the saddle equilibrium point corresponds to a separatrix between ictal and normal states (Fig. 1*B*). The trajectory behavior in Figure 1*A* corresponds to a recurrent alternation between ictal and normal states, which characterizes SLEs.

- When *x*_0_ = −2.5 and *m* = 0 or *m* = 0.5, the equilibrium point is a stable node. The trajectory behavior corresponds to a normal activity (Fig. 2*C*).
- When *x*_0_ = −0.9 and *m* = 0, the equilibrium points are: an unstable focus, a saddle, and a stable focus (Fig. 2*B*). The Epileptor remains in a nonoscillatory state (Fig. 2*D*).
- When *x*_0_ = −0.9 and *m* = 0.5, three equilibrium points coexist. The equilibrium points are as follows: one saddle and two unstable foci (Fig. 2*A*). The trajectory behavior corresponds to a periodic solution (Fig. 2*E*).

##### Parameter space of equilibrium points

**Figure 3.**
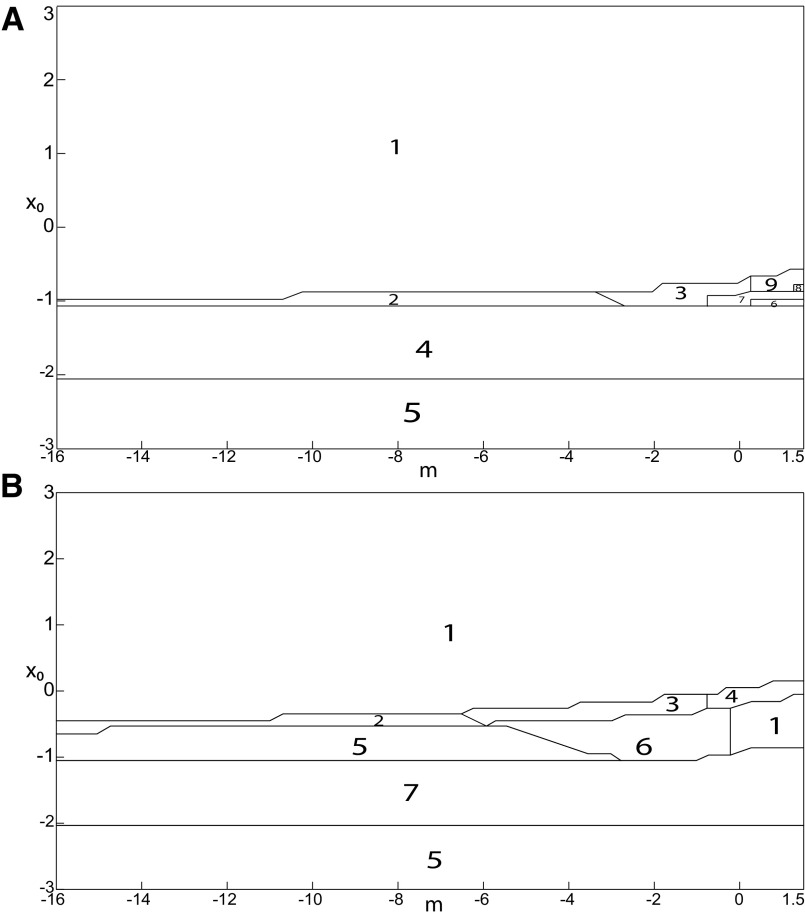
Parameter space for equilibrium points. ***A***, ***B***, There are 9 regions in *I*_ext2_ = 0.45 (*A*) and 7 regions in *I*_ext2_ = 0 (*B*). The description of both parameter spaces is found in the Results section (Epileptor dynamics, Parameter space of equilibrium points).

The stability of equilibrium points changes when we vary *m* and *x*_0_ (Fig. 2*A*,*B*). Trajectories can exhibit: (1) a normal activity when an equilibrium point is a stable node, (2) a nonoscillatory state when an equilibrium point is a stable focus, (3) SLEs when a unique saddle equilibrium point exists, and (4) a periodic solution when equilibrium points are unstable. To explore the various coexisting equilibrium points, we plot a (*m*, *x*_0_) parameter space of equilibrium points in Figure 3*A*.

The parameter space comprises nine areas. The equilibrium point is an unstable focus in area 1, a stable node in area 5, and a saddle in area 4. The Epileptor model has three equilibrium points in areas 2, 3, 6, 7, 8, and 9. In area 2, a stable node, a saddle and an unstable focus coexist. In area 3, a stable focus, a saddle, and an unstable focus coexist. In area 6, three saddles coexist. In area 7, two saddles and one unstable focus coexist. In area 9, two unstable foci and one saddle coexist. In area 8, an unstable focus, an unstable node, and a saddle coexist.

We plot a (*m*, *x*_0_) parameter space of equilibrium points for $I_{ext_{2}}=0$ in Figure 3*B*. The parameter space comprises seven areas. The equilibrium point is an unstable focus in area 1, a stable node in area 5, a stable focus in area 6, and a saddle in area 7. The Epileptor model has three equilibrium points in areas 2, 3, and 4. In area 2, a stable node, a saddle, and an unstable focus coexist. In area 3, a stable focus, a saddle, and an unstable focus coexist. In area 4, two unstable foci and one saddle coexist.

##### Main parameters of the Epileptor model

*m* and *x*_0_ play roles of particular importance on the Epileptor dynamics. We showed above that the equilibrium points of the Epileptor change as *x*_0_ varies, thereby allowing the obtaining of different behaviors of the system, including normal activity, nonoscillatory state (Fig. 2), and the alternation between the normal and ictal states (Fig. 1).

*m* can be considered as a parameter that controls the Epileptor dynamic during the ictal period. The equilibrium points of the Epileptor model are graphically defined by the intersection of the nullclines. The *x*_1_-nullcline (${\dot{x}}_{1}=0$) is a straight line for $x_{1}\geq 0$. The sign of the slope of the straight line varies the direction of movement of the state variable *x*_1_ in the phase portrait ($\forall x_{1}\geq 0$). Then, the stability of the equilibrium points ($\forall x_{1}\geq 0$) changes when varying the slope, which depends on *m*, and hence the dynamic of the Epileptor can pass from fast discharges to nonoscillatory state during the ictal period as *m* varies.

Below, we theoretically demonstrate how the system can switch between normal and epileptic activities by varying *x*_0_ and using bifurcation diagrams. Moreover, we explore the significant role of *m* in controlling the frequency of discharges and in generating a DB.

##### Bifurcation diagram of the Epileptor for *m *=* *0.5

**Figure 4.**
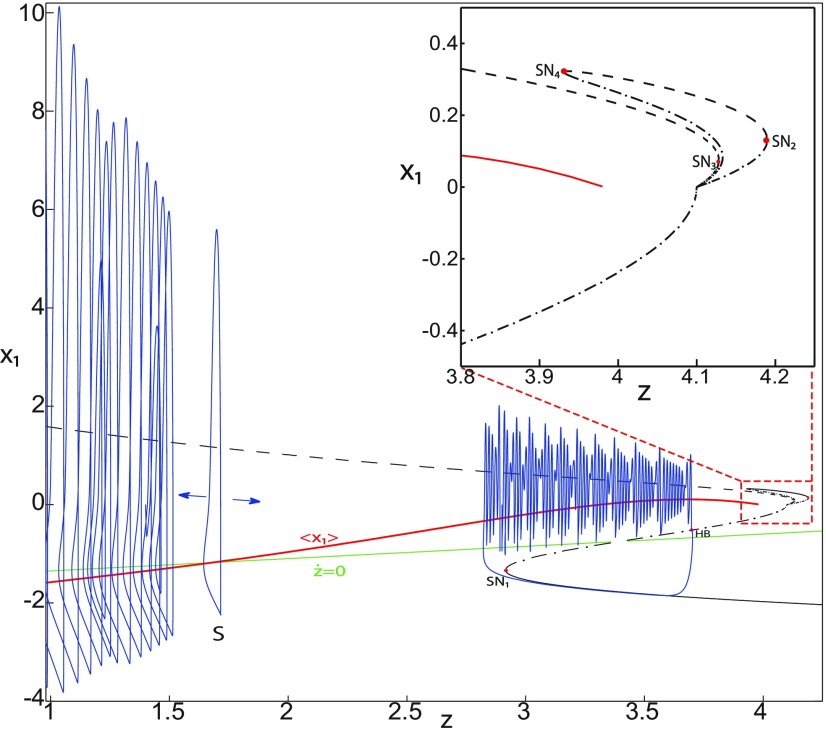
The Epileptor model bifurcation diagram with respect to the slow variable *z* (*m * = * *0.5, *I*_ext2_ = 0.45). The *Z*-lower (solid), *Z*-middle (dash-dotted), and *Z*-upper (dashed) branches consist of stable nodes, saddles, and unstable foci, respectively. Decreasing *z*, the *Z*-lower and *Z*-middle branches collide in an SN_1_ bifurcation. Above the *Z*-curve, lower (dash-dotted) branch consists of saddles, and upper branch is divided into two sub-branches: one sub-branch (dashed) consists of unstable foci and another (dash-dotted) of saddles. Increasing *z*, the two sub-branches collide in an SN_2_ bifurcation. The inset is their enlarged view. Decreasing *z*, upper (dashed) and lower branches above the *Z*-curve collide in an SN_4_ bifurcation. Increasing *z*, the *Z*-upper branch and lower branch above collide in an SN_3_ bifurcation. The $<x_{1}>$-curve is the average value of *x*_1_ for each z constant. Let *x*_0_ = −1.6, the *z*-nullcline ($\dot{z}=0$) is at the *Z*-middle branch. A SLE occurs with a fold/homoclinic bifurcation. A saddle (*S*) periodic orbit separates the SLE attractor (right) and a stable periodic orbit LC (to the left, final orbit not shown). Deterministic trajectories are plotted on both sides of the separatrix S defining two basins of attraction (indicated by arrows). *r * = * *0.003 for LC and *r * = * *0.0007 for SLEs.

The Epileptor can alternate between ictal and normal states (see *ψ*; Fig. 1) when the equilibrium point is a saddle (see *x*_0_ = −1.6; Fig. 2). The alternation is interpreted mathematically as a bifurcation, which is a qualitative change of the system behavior (Izhikevich, 2007). We identify two bifurcation types in an SLE. The first bifurcation corresponds to the transition from normal to ictal activity. The second one corresponds to the transition from ictal to normal activity. We identify bifurcation types and find the equilibrium points in a bifurcation diagram. Using the Matlab solve symbolic solver, we plot a (*z*, *x*_1_) bifurcation diagram of the Epileptor model in Figure 4 for *m * = * *0.5. *x*_1_ is the first component of the equilibrium points *E*. *z* acts as a control parameter (*r * = * *0). Figure 4 first shows a *Z*-shaped curve (*Z*-curve). *Z*-lower and *Z*-upper branches consist of stable nodes and unstable foci, respectively. The *Z*-middle branch separates *Z*-lower and *Z*-upper branches and consists of saddles. The *Z*-middle and *Z*-upper branches collide as *z* increases in a saddle-node bifurcation SN_3_ (Fig. 4, inset). The *Z*-middle and *Z*-lower branches collide as *z* decreases in a saddle-node bifurcation SN_1_.

Moreover, there are two branches above the *Z*-shaped curve (Fig. 4, inset). The lower branch consists of saddles and terminates as *z* decreases in a saddle-node bifurcation SN_4_. The upper branch comprises two sub-branches: one sub-branch (dashed) consists of unstable foci and another (dash-dotted) consists of saddles. The two sub-branches collide as *z* increases in a saddle-node bifurcation SN_2_. The equilibrium points *E* of the Epileptor model lie at the intersection of the *z*-nullcline and the curve of equilibrium points (Fig. 4). The *z*-nullcline ($\dot{z}=0$) is given by the following:

(33) $$\dot{x_{1}}=\{\begin{array}{ll} (z^{7}+10z+10sx_{0})/10s & \;\text{if}\;z<0\\ (z+sx_{0})/s & \;\text{if}\;z\geq 0 \end{array}$$

and depends on *x*_0_. The *z*-nullcline moves downward (*x*_0_ decreases) or upward (*x*_0_ increases) in the bifurcation diagram; hence, it intersects the curve of equilibrium points at different sites. When *x*_0_ = −1.6, then the *z*-nullcline intersects the *Z*-middle branch, which consists of saddles (Fig. 4). Consistent with Figure 2*A*, the equilibrium point is a saddle. The trajectory behavior corresponds to SLEs, which is illustrated in Figure 4 (right). When a trajectory is at the *Z*-lower branch, the stable node disappears as *z* decreases through a saddle-node bifurcation SNs_1_. Then the trajectory switches to the *Z*-upper branch, which consists of unstable foci surrounded by stable periodic orbits. Hence the trajectory exhibits an oscillatory solution on the *Z*-upper branch, which approaches the *Z*-middle branch as *z* increases. The oscillatory solution is homoclinic to one of the saddles along the *Z*-middle branch, then it is destroyed as *z* is increased. The trajectory terminates in a homoclinic bifurcation HB and switches to the *Z*-lower branch. *Z*-lower and *Z*-upper branches correspond to normal and ictal states, respectively. The *Z*-middle branch acts as a separatrix between ictal and normal states. The transition from normal to ictal states (first bifurcation) occurs through a saddle-node bifurcation, SN_1_. The transition from ictal to normal states (second bifurcation) occurs though a homoclinic bifurcation, HB. The homoclinic bifurcation *z*(HB) corresponds to an offset threshold and the saddle-node bifurcation $z(SN_{1})$ corresponds to an onset threshold. Therefore, the transitions between ictal and normal states occur through a fold/homoclinic bifurcation. The system is bistable on [SN_1_, HB].

##### Bifurcation diagram when *m* decreases

**Figure 5.**
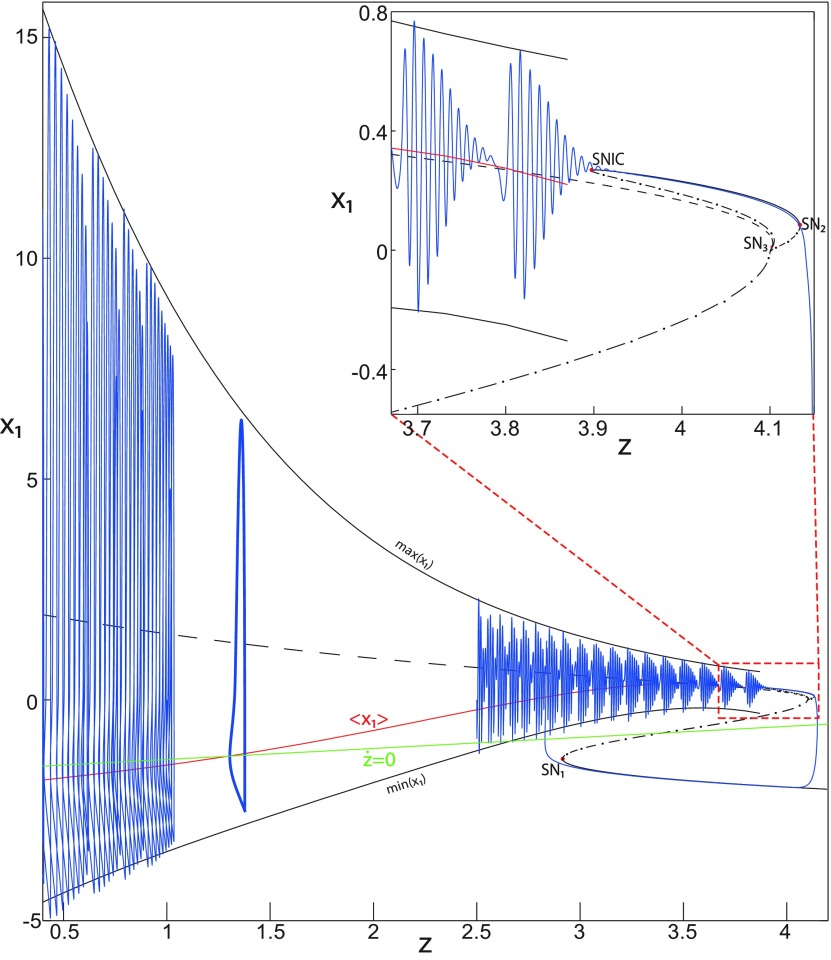
The Epileptor model bifurcation diagram with respect to the slow variable *z* (*m * = * *0, $I_{ext2}=0.45$). The *Z*-lower (solid), *Z*-middle (dash-dotted), and *Z*-upper (dashed) branches consist of stable nodes, saddles, and unstable foci, respectively. Decreasing *z*, the *Z*-lower and *Z*-middle branches collide in an SN_1_ bifurcation. Above the *Z*-curve, lower (dash-dotted) branch consists of saddles, and the upper branch is divided into two sub-branches: one (solid) consists of stable foci and another (dash-dotted) of saddles. Increasing *z*, the two sub-branches collide in an SN_2_ bifurcation. The inset is their enlarged view. Decreasing *z*, upper (dashed) and lower branches above the *Z*-curve collide in a SNIC bifurcation. Increasing *z*, the *Z*-upper branch and lower branch above collide in an SN_3_ bifurcation. The $<x_{1}>$-curve is the average value of *x*_1_ for each z constant. Let *x*_0_ = −1.6, the *z*-nullcline ($\dot{z}=0$) is at the *Z*-middle branch. A SLE occurs with a fold/circle bifurcation. A saddle (*S*) periodic orbit separates the SLE attractor (right) and a stable periodic orbit LC (to the left, final orbit not shown). Deterministic trajectories are plotted on both sides of the separatrix S defining two basins of attraction (indicated by arrows). For the right trajectory, *r * = * *0.0006, I.C = [0 −5 2.5 0 0 0.01] and *T_s_* = [0 1337]. For the left trajectory, *r * = * *0.001, I.C = [0 −5 1 0 0 0.01], and *T_s_* = [0 1000].

To explore the role of *m* in the dynamics of the Epileptor, we plot a (*z*, *x*_1_) bifurcation diagram, when *m* decreases. Let *m * = * *0 (Fig. 5), the plot first shows a *Z*-shaped curve. *Z*-Lower, *Z*-middle, and *Z*-upper branches consist of stable nodes, saddles, and unstable foci, respectively.

The *Z*-upper branch is surrounded by stable periodic orbits (see $max(x_{1})$ and $min(x_{1})$ curves), which terminate as *z* increases in a saddle-node on invariant circle (SNIC) bifurcation with $z(SNIC)>z(SN_{1})$. Above the *Z*-curve, the lower branch consists of saddles and the upper branch comprises two sub-branches (Fig. 5, inset). The first sub-branch (solid) consists of stable foci and terminates as *z* decreases in a SNIC bifurcation. The second sub-branch (dash-dotted) consists of saddles. The two sub-branches collide as *z* increases in a saddle-node bifurcation SN_2_. We plot a trajectory in Figure 5 (right), which corresponds to SLEs. When a trajectory is at the *Z*-lower branch, the stable node disappears as *z* decreases through a saddle-node bifurcation SN_1_ and the trajectory switches to the *Z*-upper branch exhibiting an oscillatory solution, which terminates as *z* increases in a SNIC bifurcation (see inset). Then the trajectory is at the upper branch above the *Z*-curve, which consists of stable foci. The trajectory exhibits a nonoscillatory solution, which terminates as *z* increases in a saddle-node bifurcation SN_2_ and the trajectory switches to the *Z*-lower branch. The *Z*-lower branch corresponds to the normal state. The *Z*-upper branch and the upper branch above the *Z*-curve correspond to the ictal state (see inset). The transition from normal to ictal states (first bifurcation) occurs through a saddle-node bifurcation, SN_1_. The transition from ictal to normal states (second bifurcation) occurs through a saddle-node bifurcation, SN_2_. Fast discharges characterizing the ictal state correspond to the oscillatory solution, which disappears through a saddle-node on invariant circle bifurcation SNIC. Therefore, the transitions between ictal and normal states are said to be of a fold/circle bifurcation. The system is bistable on [SN_1_, SN_2_].

We conclude that the bifurcation diagrams of the Epileptor model is a *Z*-shaped curve. Above, there are the following three branches: two branches consisting of saddles, and one branch consisting of stable foci for *m *≤* *0 and unstable foci for *m *>* *0. The Epileptor model undergoes a SNIC bifurcation for *m *≤* *0 and an HB bifurcation for *m *>* *0. The transitions between ictal and normal states occur through a fold/circle bifurcation for *m *≤* *0 and through a fold/homoclinic bifurcation for *m * >* * 0. The system is bistable on [SN_1_, SN_2_] for *m * ≤ * *0 and on [SN_1_, HB] for *m *>* *0.

##### Bifurcation diagram for *m *=* *0 and $I_{ext_{2}}=0$

**Figure 6.**
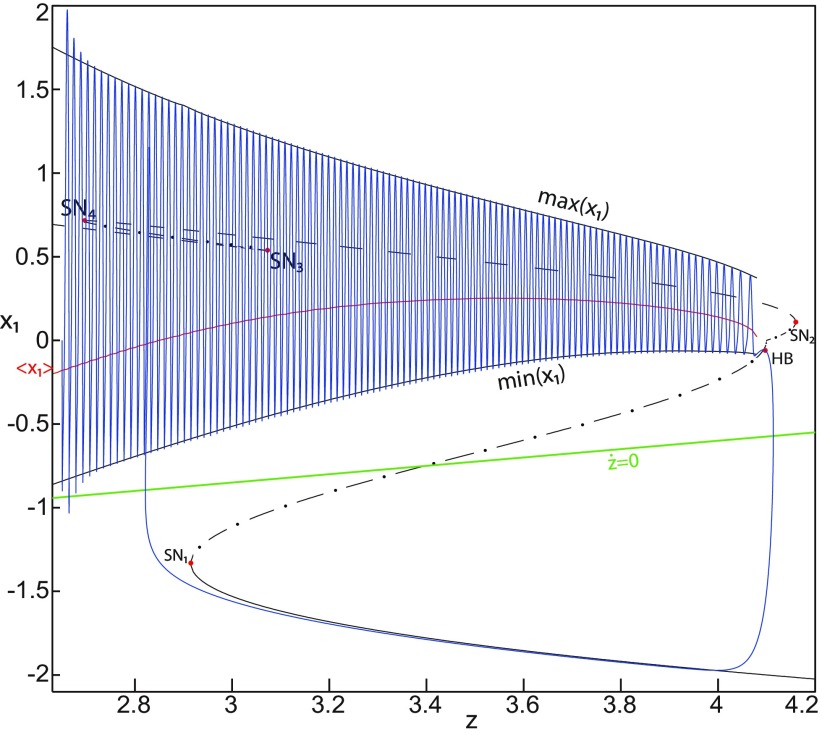
The Epileptor model bifurcation diagram with respect to the slow variable *z* (*m * = * *0, *I*_ext2_ = 0). The *Z*-lower (solid), *Z*-middle (dash-dotted), and *Z*-upper (dashed) branches consist of stable nodes, saddles, and unstable foci, respectively. Decreasing *z*, the *Z*-lower and *Z*-middle branches collide in an SN_1_ bifurcation. Increasing *z*, the *Z*-upper and *Z*-middle branches collide in an SN_2_ bifurcation. Below the *Z*-upper branch, lower (dashed) and upper (dash-dotted) branches corresponding to unstable foci and saddles, respectively, collide in an SN_3_ bifurcation. The *Z*-upper branch and the upper (dash-dotted) branch below collide as *z* decreases in an SN_4_ bifurcation. The $<x_{1}>$-curve is the average value of *x*_1_ for each *z* constant. Let $x_{0}=-1.6$, the *z*-nullcline ($\dot{z}=0$) is at the *Z*-middle branch. A SLE occurs with a fold/homoclinic bifurcation. For the (deterministic) trajectories, *r * = * *0.0005, I.C = [0 −5 2.5 0 0 0.01] and *T_s_* = [0:0.001: 1540].

We now analyze the bifurcation diagram when $I_{ext_{2}}$ decreases. Let $I_{ext_{2}}=0$, we plot a (*z*, *x*_1_) bifurcation diagram for *m * = * *0 in Figure 6, which shows a *Z*-shaped curve. *Z*-lower, *Z*-middle, and *Z*-upper branches consist of stable nodes, saddles, and unstable foci, respectively. Decreasing *z*, *Z*-lower and *Z*-middle branches collide in a saddle-node bifurcation, SN_1_. Increasing *z*, *Z*-upper and *Z*-middle branches collide in another saddle-node bifurcation, SN_2_. The *Z*-upper branch terminates as *z* decreases in a saddle-node bifurcation SN_4_. Moreover, two branches appear as *z* decreases. The lower branch (dashed) consists of unstable foci, and the upper branch (dash-dotted) consists of saddles. Increasing *z*, lower and upper branches collide in a saddle-node bifurcation SN_3_. Decreasing *z*, the upper branch terminates in a saddle-node bifurcation SN_4_.

Let *x*_0_ = −1.6, the *z*-nullcline is at the *Z*-middle branch, then the equilibrium point is a saddle. We plot a trajectory in Figure 6, which shows transitions between *Z*-lower and *Z*-upper branches. When a trajectory is at the *Z*-lower branch, the stable node disappears as *z* decreases through a saddle-node bifurcation, SN_1_, and the trajectory switches to the *Z*-upper branch, which consists of unstable foci. An unstable focus is surrounded by a stable periodic orbit (see $max(x_{1})$ and $min(x_{1})$ curves). Hence, when the trajectory is at the *Z*-upper branch, it exhibits an oscillatory solution, which terminates as *z* increases in a homoclinic bifurcation HB. Then the trajectory switches to the *Z*-lower branch. *Z*-Lower (solid) and *Z*-upper (dashed) branches correspond to normal and ictal states, respectively. The transition from normal to ictal states occurs through a saddle-node bifurcation SN_1_. The transition from ictal to normal states occurs through an HB. Thus, the transitions between ictal and normal states occur through a fold/homoclinic bifurcation. The system is bistable on [SN_1_, HB].

##### Bifurcation diagram when *m* decreases, for $I_{ext_{2}}=0$

**Figure 7.**
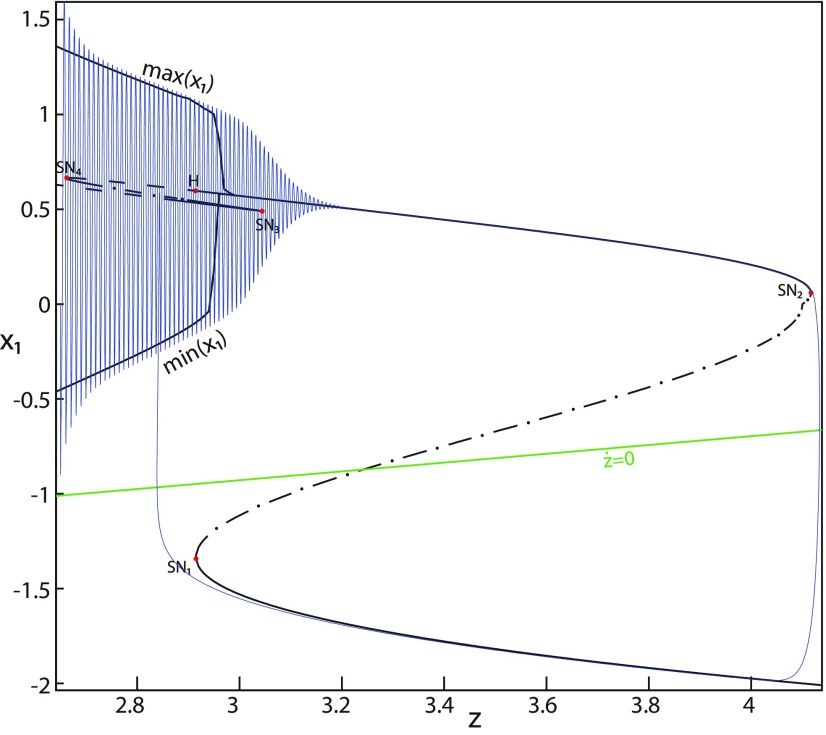
The Epileptor model bifurcation diagram with respect to the slow variable *z* (*m* = −0.5, *I*_ext2_ = 0). The *Z*-lower (solid) and *Z*-middle (dash-dotted) branches consist of stable nodes and saddles respectively. The *Z*-upper branch is divided into sub-branches separated by a Hopf bifurcation, *H*: one sub-branch (solid) consists of stable foci and another (dashed) consists of unstable foci. Decreasing *z*, the *Z*-lower and *Z*-middle branches collide in an SN_1_ bifurcation. Increasing *z*, the *Z*-upper (solid) and *Z*-middle branches collide in an SN_2_ bifurcation. Below the *Z*-upper branch, lower (dashed) and upper (dash-dotted) branches corresponding to unstable foci and saddles, respectively, collide in an SN_3_ bifurcation. The *Z*-upper (dashed) branch and the upper (dash-dotted) branch below collide as *z* decreases in an SN_4_ bifurcation. Let $x_{0}=-1.6$, the *z*-nullcline ($\dot{z}=0$) is at the *Z*-middle branch. A SLE occurs with a fold/Hopf bifurcation. For the (deterministic) trajectories, *r * = * *0.0007, I.C = [0 −5 2.65 0 0 0.01] and *T_s_* = [0:0.001: 1028].

Let *m* = −0.5, we plot a (*z*, *x*_1_) bifurcation diagram in Figure 7. The *Z*-upper branch is divided into two sub-branches separated by a Hopf (H) bifurcation (Fig. 7). The first sub-branch (solid) consists of stable foci and terminates as *z* increases in a saddle-node bifurcation SN_2_. The second sub-branch (dashed) consists of unstable foci and terminates as *z* decreases in a saddle-node bifurcation SN_4_. An unstable focus is surrounded by a stable periodic orbit (see $max(x_{1})$ and $min(x_{1})$ curves), which terminates as *z* increases in an H bifurcation. When *x*_0_ = −1.6, then the *z*-nullcline is at the *Z*-middle branch, hence the equilibrium point is a saddle. We plot a trajectory in Figure 7, which shows transitions between *Z*-lower and *Z*-upper branches. When a trajectory is at the *Z*-lower branch, the stable node disappears as *z* decreases through a saddle-node bifurcation, SN_1_, and the trajectory switches to the *Z*-upper sub-branch (dashed) exhibiting an oscillatory solution, which terminates as *z* increases in an H bifurcation. Then, the trajectory is at the *Z*-upper (solid) sub-branch exhibiting a nonoscillatory solution, which terminates as *z* increases in a saddle-node bifurcation SN_2_. *Z*-lower (solid) and *Z*-upper branches correspond to normal and ictal states, respectively. The oscillatory solution corresponds to fast discharges. The transition from normal to ictal states occurs through a saddle-node bifurcation, SN_1_. The transition from ictal to normal states occurs through a saddle-node bifurcation SN_2_. The oscillatory solution (fast discharges) terminates in an H bifurcation. The transitions between ictal and normal states are said to be of a fold/Hopf bifurcation. The system is bistable on [SN_1_, SN_2_].

**Figure 8.**
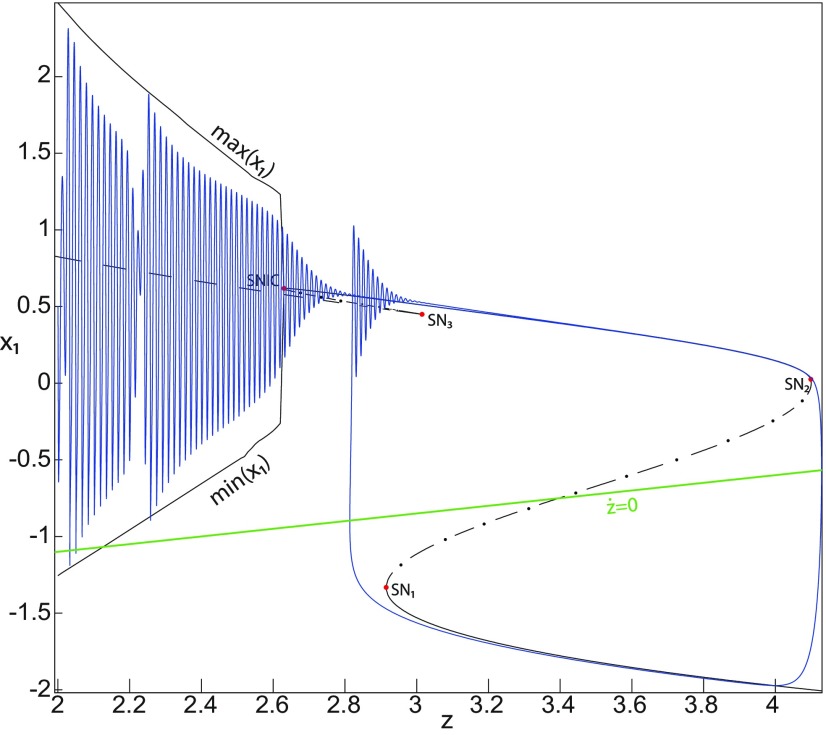
The Epileptor model bifurcation diagram with respect to the slow variable *z* (*m* = − 1, *I*_ext2_ = 0). The *Z*-lower (solid), *Z*-middle (dash-dotted), and *Z*-upper (solid) branches consist of stable nodes, saddles, and stable foci, respectively. Decreasing *z*, the *Z*-lower and *Z*-middle branches collide in an SN_1_ bifurcation. Increasing *z*, the *Z*-upper and *Z*-middle branches collide in an SN_2_ bifurcation. Below the *Z*-upper branch, lower (dashed) and upper (dash-dotted) branches corresponding to unstable foci and saddles respectively, collide in an SN_3_ bifurcation. The *Z*-upper (solid) branch and the upper (dash-dotted) branch below collide as *z* decreases in a SNIC bifurcation. Let *x*_0_ = −1.6, the *z*-nullcline ($\dot{z}=0$) is at the *Z*-middle branch. A SLE reduces to a periodic switch between a nonoscillatory state and a NS, which occurs through a fold/fold bifurcation. For the (deterministic) trajectories, *r * = * *0.0008, I.C = [0.5 −5 2 0 0 0.01] and *T_s_* = [0:0.001: 1400].

Let *m* = −1, we plot a (*z*, *x*_1_) bifurcation diagram in Figure 8, which has a *Z*-shaped curve. The Hopf bifurcation point H disappears, then the *Z*-upper branch consists of stable foci only, which is the equilibrium point of nonoscillatory state. Increasing *z*, the *Z*-upper (solid) branch terminates in a SNIC bifurcation (Fig. 8). Below the *Z*-upper branch, two branches appear as *z* decreases: the upper (dash-dotted) branch consists of saddles, and the lower (dashed) one consists of unstable foci. An unstable focus is surrounded by a stable periodic orbit (see $max(x_{1})$ and $min(x_{1})$ curves), which terminates as *z* increases in a SNIC bifurcation. Let *x*_0_ = −1.6, the *z*-nullcline is at the *Z*-middle branch, then the equilibrium point is a saddle. We plot a trajectory in Figure 8, which shows transitions between *Z*-lower and *Z*-upper branches. When a trajectory is at the *Z*-lower branch, the stable node disappears as *z* decreases through a saddle-node bifurcation, SN_1_, and the trajectory switches to the *Z*-upper (solid) branch, which consists of stable foci, thereby exhibiting a nonoscillatory solution. Then, the stable focus disappears as *z* increases through a saddle-node bifurcation, SN_2_, and the trajectory switches to the *Z*-lower branch. The transition from *Z*-lower to *Z*-upper branches occurs through a saddle-node bifurcation, SN_1_, and the transition from *Z*-upper to *Z*-lower branches occurs through a saddle-node bifurcation, SN_2_. The transitions between *Z*-lower and *Z*-upper branches occur through a fold/fold bifurcation. The system is bistable on [SN_1_, SN_2_]. Here, the SLE attractor reduces to a periodic switch between a nonoscillatory state and a NS.

We conclude that when $I_{ext_{2}}=0$, the transitions between *Z*-lower and *Z*-upper branches occur through a fold/homoclinic bifurcation, ∀m≥0; a fold/Hopf bifurcation for *m* = −0.5; and a fold/fold bifurcation, ∀m<−0.5. The system is bistable on [SN_1_, SN_2_] for *m *<* * 0 and on [SN_1_, HB] for *m * ≥* * 0. Moreover, the transitions between ictal and normal states occur through a fold/circle bifurcation when $I_{ext_{2}}=0.45$, and do not when $I_{ext_{2}}=0$.

#### Finding LC: a stable limit cycle

##### LC in the Epileptor model

**Figure 9.**
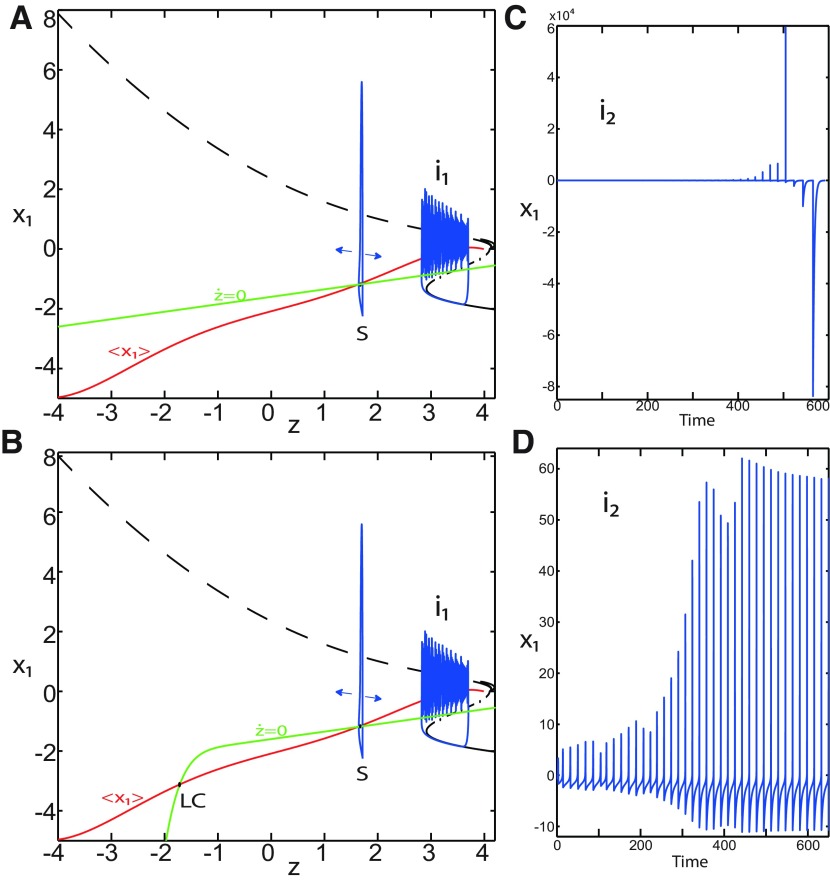
On the LC existence and dynamics. The Epileptor model bifurcation diagram (*z*, *x*_1_) with respect to *z* (*m * = * *0.5, *I*_ext2_ = 0.45, *x*_0_ = −1.6). ***A***, ***B***, The *z*-nullcline is given by Equation 34 (***A***), and results from Equation 35 (***B***). The $<x_{1}>$-curve is the average value of *x*_1_ for each z constant. ***C***, ***D***, Characteristic time series (deterministic) show a divergence of the Epileptor with time (***C***), and the fast-slow limit cycle LC (***D***). *r * = * *0.009 for LC (*i*_2_) and *r * = * *0.0007 for SLEs (*i*_1_).

We found a large geometrical object in the (*y*_1_, *ψ*, *z*) phase space below the SLE attractor shown in Figure 1. When using the *z* equation (Eq. 34), the Epileptor diverges with time (Fig. 9*C*). The initial conditions *i*_2_ are on the left of the separatrix (*S*), which means that *i*_2_ are below the separatrix (S) and the SLE attractor in the phase space. To explain this divergence, we plot the *z*-nullcline and the $<x_{1}>$-curve in a (*z*, *x*_1_) bifurcation diagram for *m * = * *0.5 (Fig. 9*A*). The *z*-nullcline corresponds to

(34) $$z=s(x_{1}-x_{0}).$$

When *x*_0_ = −1.6, then the *z*-nullcline intersects the $<x_{1}>$-curve at one periodic orbit labeled as S (Fig. 9*A*). We consider two different initial conditions *i*_1_ and *i*_2_ such as $z(i_{2})<z(S)<z(i_{1})$. Only the trajectory *i*_1_ is plotted in the bifurcation diagram, which exhibits transitions between *Z*-lower and *Z*-upper branches through a fold/homoclinic bifurcation. We plot a time series for the initial condition *i*_2_ in Figure 9*C*, which shows that the trajectory diverges with time.

To stabilize the final state that the Epileptor evolves toward, we modified the *z*-equation by introducing the following equation:

(35) $$\dot{z}=\{\begin{array}{ll} r(s(x_{1}-x_{0})-z-0.1z^{7}) & \text{if}\;z<0\\ r(s(x_{1}-x_{0})-z) & \text{if}\;z\geq 0 \end{array}$$

We plot the corresponding *z*-nullcline ($\dot{z}=0$; Eq. 35) in a ($z,x_{1}$) bifurcation diagram for *m * = * *0.5 (Fig. 9*B*). When *x*_0_ = −1.6, then the *z*-nullcline intersects the $<x_{1}>$-curve at two periodic orbits: S and LC (Fig. 9*B*). We consider the initial conditions *i*_1_ and *i*_2_ such as $z(i_{2})<z(S)<z(i_{1})$. The trajectory *i*_1_ exhibits transitions between *Z*-lower and *Z*-upper branches, which occur through a fold/homoclinic bifurcation. We plot time series for the initial condition *i*_2_ in Figure 9*D*, which shows a stable LC. We plot the trajectory as its transients toward LC in Figure 4 and the corresponding time series in Figure 9*D*. LC is characterized by a large amplitude and a fast-slow invariant cycle (Fig. 9*D*).

**Figure 10.**
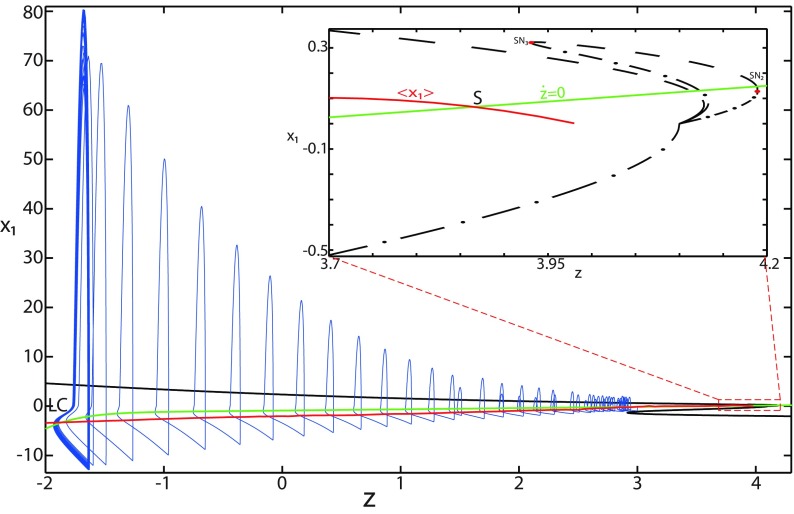
On the LC existence and dynamics. The Epileptor model bifurcation diagram (*z*, *x*_1_) with respect to *z* as plotted in Figure 4. The $<x_{1}>$-curve is the average value of *x*_1_ for each *z* constant. Let *x*_0_ = −0.9, the *z*-nullcline is at three branches of the (*z*, *x*_1_) curve. Two branches (dashed) consist of unstable foci and one branch (dash-dotted) consists of saddles (see inset). Deterministic trajectory converges to LC. The final state is the intersection of the *z*-nullcline and $<x_{1}>$-curve. *m * = * *0.5, *I*_ext2_ = 0.45, *r * = * *0.0035, I.C = [0 −5 3 0 0 0.01] and *T_s_* = [0 1000].

We consider Equation 35 and plot the *z*-nullcline when $x_{0}=-0.9$ in a ($z,x_{1}$) bifurcation diagram (Fig. 10). The *z*-nullcline intersects three branches of the ($z,x_{1}$) curve, as follows: lower (dash-dotted) and upper (dashed) branches above the *Z*-curve, and the *Z*-upper (dashed) branch. Then three equilibrium points coexist: two unstable foci and one saddle. The *z*-nullcline intersects the $<x_{1}>$-curve at two periodic orbits, which are LC and S. Then the trajectory only exhibits an oscillatory solution, which stabilizes at the point LC.

##### Evolution of periodic orbits

We plot the $max(x_{1})$- and $min(x_{1})$-curves to limit the oscillatory solutions (Figs. 5–8). Decreasing *z*, trajectories converge to LC and the amplitude of periodic orbits increases, leading to LC with a large amplitude. The intersection of the *z*-nullcline and the $<x_{1}>$-curve gives rise to S and LC, which are saddle and stable periodic orbits, respectively.

Let *x*_0_ = −1.6, two periodic orbits exist for *m * = * *0.5 (Fig. 4) and *m * = * *0 (Fig. 5). We show only one periodic orbit in Figures 4 and 5, which corresponds to S. The second periodic orbit is shown in Figure 9*B*, which corresponds to LC. LC and S are stable and saddle periodic orbits, respectively. Trajectories plotted in Figures 4 and 5 exhibit SLEs (right) or converge to LC (left), depending on the initial conditions. SLEs and LC are considered as two attractors. The saddle periodic orbit (S) acts as a separatrix between the basin of attraction of SLEs and the basin of attraction of LC.

**Figure 11.**
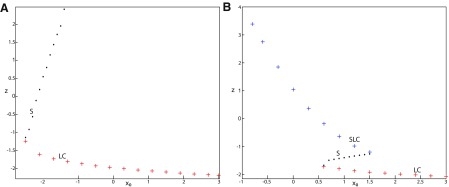
***A***, ***B***, Finding periodic orbits of the Epileptor for *m * = * *0.5 (***A***) and *m* = −16 (***B***). Stable periodic orbits LC and SLC are labeled as red plus sign markers (bottom) and blue plus sign markers (top), respectively. Saddle periodic orbits S are labeled as black dots. ***A***, ***B***, Decreasing *x*_0_, periodic orbits LC and S disappear through a saddle-node of periodic orbits bifurcation. ***B***, Decreasing further *x*_0_ (below −0.8), SLC disappears.

The *z*-nullcline moves downward (*x*_0_ decreases) or upward (*x*_0_ increases) in the bifurcation diagram, hence the existence of periodic orbits depends on *x*_0_. We find S and LC in a (*x*_0_, z^*^) bifurcation diagram of periodic orbits (Fig. 11*A*). *x*_0_ acts as a control parameter. Finding S helps us to limit the basin of attraction of SLEs and the basin of attraction of LC. Finding LC helps us to determine the *z*-value at which LC stabilizes. Red (+) markers and black dots correspond to LC and S, respectively. Only LC exists for large *x*_0_. Decreasing *x*_0_, LC and S coexist, and collide as *x*_0_ is further decreased in a saddle-node of periodic orbits bifurcation (SNPO), then fades.

Figure 11*B* (*m* = −16) shows that for some *x*_0_, a third periodic orbit appears, which is labeled as blue top (+) markers. This periodic orbit is stable, and is localized above LC and S (Fig. 11*B*). Since the amplitude of periodic orbits decreases as *z* increases, the third periodic orbit is a stable limit cycle with a small amplitude, which we denoted as small limit cycle (SLC). SLC and LC are two stable limit cycles that coexist for some *x*_0_, and are separated by S. SLC exists after a SNPO bifurcation occurs when *m* = −16 and disappears when *x*_0_ is further decreased.

##### Parameter space of periodic orbits

**Figure 12.**
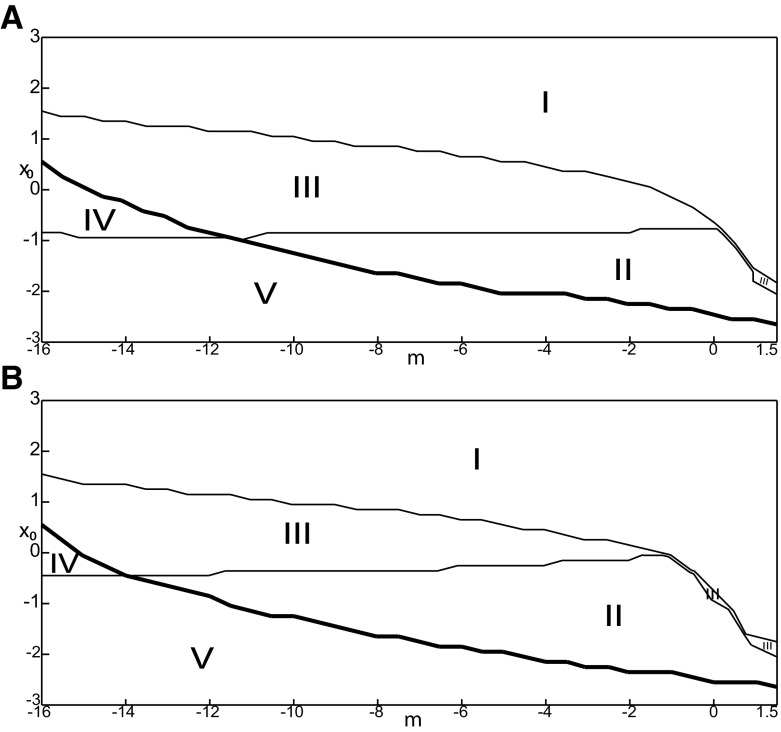
Parameter space of periodic orbits. ***A***, ***B***, There are five regions in *I*_ext2_ = 0.45 (***A***) and *I*_ext2_ = 0 (***B***). LC exists in area I and coexists with S in area II. Only SLC exists in area IV, and coexists with LC and S in area III. LC, SLC, and S disappear in area V. We can visualize in Figure 5 the coexistence of LC and S (*A*, area II), and in Figure 13 the coexistence of LC, *S*, and SLC (*B*, area III).

We explore two (*m*, *x*_0_) parameter spaces of periodic orbits: for $I_{ext_{2}}=0.45$ in Figure 12A and for $I_{ext_{2}}=0$ in Figure 12*B*. There are five areas separated by a bold line (SNPO bifurcation). LC exists above, but not below. LC exists in area I and coexists with S in area II. LC and S coexist with SLC in area III. Only SLC exists in area IV, which is localized below a SNPO bifurcation (bold line). Periodic orbits disappear in area V.

#### SLC dynamics

We used the averaging method to find periodic orbits, which lie at the intersection of the *z*-nullcline and the $<x_{1}>$-curve. When $I_{ext_{2}}=0$, area III shows the coexistence of LC, SLC, and S (Fig. 12*B*).

**Figure 13.**
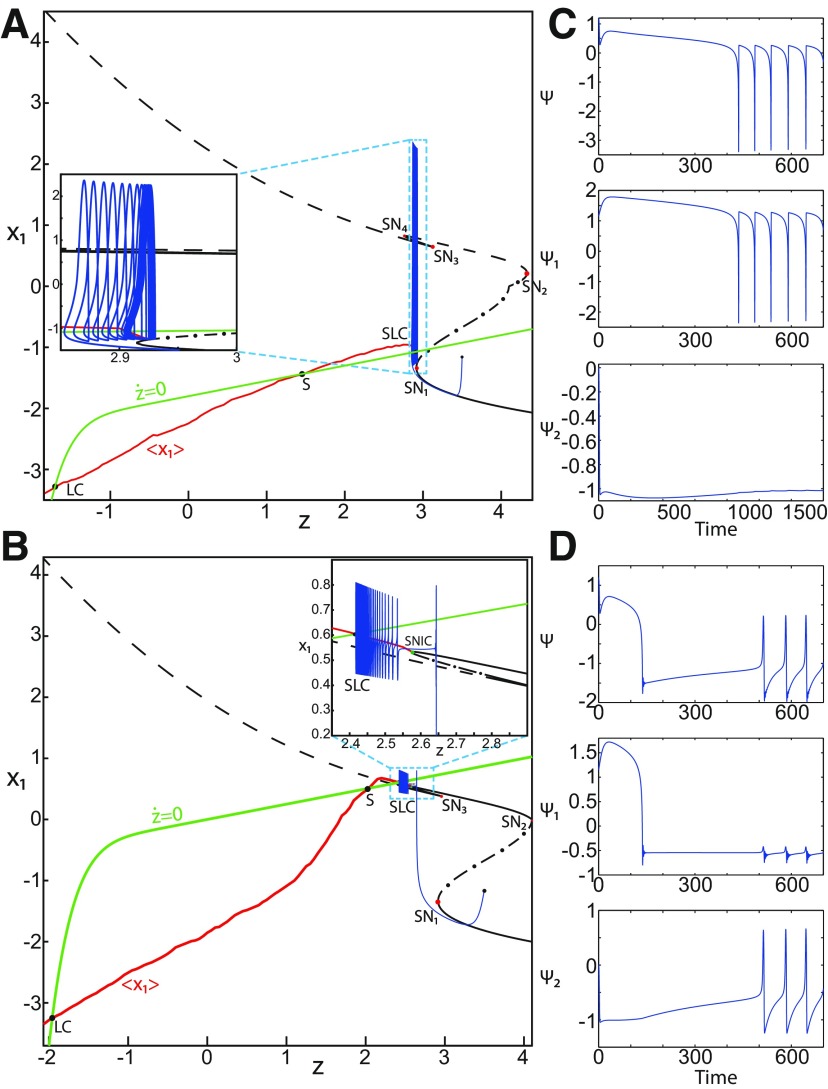
SLC dynamics. ***A***, ***B***, The equilibrium point is a saddle (*m * = * *1, *x*_0_ = −1.8; ***A***) and an unstable focus (*m* = −2, *x*_0_ = 0; ***B***) with *I*_ext2_ = 0, *r * = * *0.0007. ***C***, ***D***, Deterministic time series of the Epileptor system *ψ*, subsystem 1 *ψ*_1_ and subsystem 2 *ψ*_2_ are plotted for the saddle equilibrium point (***C***) and for the unstable focus (***D***). The $<x_{1}>$-curve is the average value of *x*_1_ for each z constant.

The stability of the equilibrium points depends on *m* and *x*_0_ in area III. We analyze the coexistence of LC and SLC for each stability type of equilibrium points. We plot a (*z*, *x*_1_) bifurcation diagram for *m *=* *1 in Figure 13*A*. When $x_{0}=-1.8$, then the *z*-nullcline is at the middle branch and the equilibrium point is a saddle. The *z*-nullcline intersects the $<x_{1}>$-curve at three periodic orbits: LC (left), S (middle), and SLC (right). LC and SLC are two stable limit cycles with large and small amplitudes, respectively. S acts as a separatrix between LC and SLC. When a trajectory is at the *Z*-lower branch, the stable node disappears as *z* decreases through a saddle-node bifurcation SN_1_ and the trajectory switches to the *Z*-upper branch, which consists of unstable foci. Then the trajectory exhibits an oscillatory solution on the *Z*-upper branch and continues to SLC at which *z* stabilizes (Fig. 13*A*, inset). The transition to the *Z*-lower branch does not occur even if the equilibrium point is a saddle. In fact, *z* stabilizes at SLC before the homoclinic bifurcation HB. The trajectory behavior corresponds to a periodic solution.

We plot a (*z*, *x*_1_) bifurcation diagram for *m* = −2 in Figure 13*B*. Let *x*_0_ = 0, the equilibrium point is an unstable focus. The *z*-nullcline intersects the $<x_{1}>$-curve at three periodic orbits (Fig. 13*B*): LC (left), S (middle), and SLC (right). S acts as a separatrix between LC and SLC. When a trajectory is at the *Z*-lower branch, the stable node disappears as *z* decreases through a saddle-node bifurcation SN_1_, and the trajectory switches to the *Z*-upper (solid) branch, which consists of stable foci. Increasing *z*, the trajectory exhibits a nonoscillatory solution on the *Z*-upper branch (solid), which terminates in a SNIC bifurcation (Fig. 13*B*, inset). Then, the trajectory is at the dashed branch exhibiting an oscillatory solution and continues to SLC at which *z* stabilizes. The transition to the *Z*-lower branch does not occur and the trajectory behavior corresponds to a periodic solution.

We conclude that when $I_{ext_{2}}=0$, then LC and SLC coexist in area III and S acts as a separatrix between them. The equilibrium point of the Epileptor model is either a saddle or an unstable focus. Time series of the Epileptor system *ψ*, subsystem 1 *ψ*_1_, and subsystem 2 *ψ*_2_ are plotted in Figure 13*C* when the equilibrium point is a saddle, and in Figure 13*D* when it is an unstable focus. The SLC patterns depend on the stability of the equilibrium point. The subsystem 2 generates a resting state when the equilibrium point is a saddle (see *ψ*_2_; Fig. 13*C*) and a spiking state when it is an unstable focus (see *ψ*_2_; Fig. 13*D*).

#### Transition from epileptiform fast discharges to a DB

The Epileptor exhibits epileptiform fast discharges in the ictal state, which corresponds to an oscillatory solution at the *Z*-upper branch of the (*z*, *x*_1_) curve. Let $I_{ext_{2}}=0$, the *Z*-upper branch consists of unstable foci when *m * = * *0 (Fig. 6), and consists of stable foci when *m* = −1 (Fig. 8). Recall that the SLE reduces to a periodic switch between a nonoscillatory state and NS when *m* = −1.

**Figure 14.**
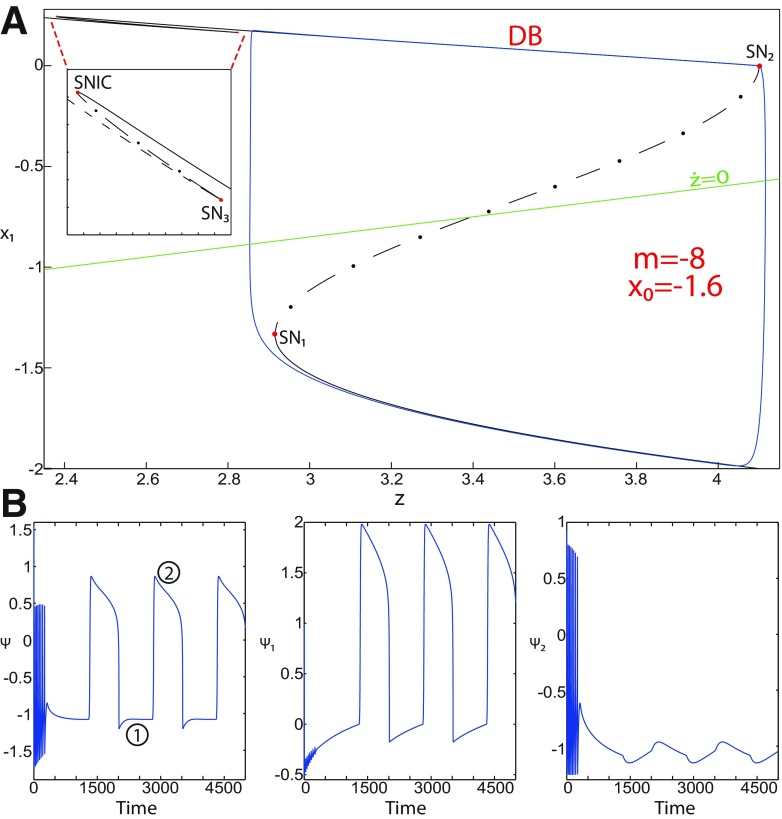
Transition from epileptiform fast discharges to depolarization block. ***A***, Bifurcation diagram (*z*, *x*_1_) of the Epileptor model. The *Z*-lower (solid), *Z*-middle (dash-dotted), and *Z*-upper (solid) branches consist of stable nodes, saddles, and stable nodes, respectively. Decreasing *z*, the *Z*-lower and *Z*-middle branches collide in an SN_1_ bifurcation. Increasing *z*, the *Z*-upper and *Z*-middle branches collide in an SN_2_ bifurcation. Below the *Z*-upper branch (see inset), the top (dash-dotted) branch consists of saddles and the bottom (dashed) branch consists of unstable foci. Increasing *z*, bottom and top branches collide in an SN_3_ bifurcation. Decreasing *z*, the *Z*-upper (solid) branch and upper (dash-dotted) branch below collide in a SNIC bifurcation. Let *x*_0_ = −1.6, the *z*-nullcline ($\dot{z}=0$) is at the *Z*-middle branch. The *Z*-upper (solid) and *Z*-lower (solid) branches correspond to DB and NS, respectively. The SLE attractor reduces then to a periodic transition between DB and NS. ***B***, Deterministic time series of the Epileptor system *ψ*, subsystem 1 *ψ*_1_, and subsystem 2 *ψ*_2_. Parameters are: *m* = − 8, *I*_ext2_ = 0, *r * = * *0.0005.

Further decreasing *m*, the imaginary part of the complex–conjugate eigenvalues goes to zero, and then the *Z*-upper branch only consists of stable nodes. We plot a (*z*, *x*_1_) bifurcation diagram for *m* = −8 ($I_{ext_{2}}=0$) in Figure 14*A*, which shows a *Z*-shaped curve. *Z*-lower (solid), *Z*-middle (dash-dotted), and *Z*-upper (solid) branches consist of stable nodes, saddles, and stable nodes, respectively. The *Z*-upper branch terminates as *z* decreases in a SNIC bifurcation (Fig. 14*A*, inset). Below the *Z*-upper branch (Fig. 14*A*, inset), lower (dashed) and upper (dash-dotted) branches appear, which approach as *m* decreases to the *Z*-upper (solid) branch. The lower branch consists of unstable foci, and the upper branch consists of saddles. Increasing *z*, the lower and upper branches collide in a saddle-node bifurcation, SN_3_. Note that $z(SNIC)<z(SN_{3})<z(SN_{1})$. Let *x*_0_ = −1.6, the equilibrium point is a saddle. When a trajectory is at the *Z*-lower branch, the stable node disappears as *z* decreases through a saddle-node bifurcation, SN_1_, and the trajectory switches to the *Z*-upper branch, which consists of stable nodes. The trajectory exhibits a silent activity on the *Z*-upper branch, which terminates as *z* increases in a saddle-node bifurcation SN_2_. Then, the trajectory switches to the *Z*-lower branch. The transition between *Z*-lower and *Z*-upper branches occur through a fold/fold bifurcation. The system is bistable on [SN_1_, SN_2_]. Time series of the Epileptor model *ψ*, subsystem 1 *ψ*_1_, and subsystem 2 *ψ*_2_ are plotted in Figure 14*B*. The fast discharges during the ictal period disappear with decreasing *m* underlying a slow wave. This scenario corresponds to a depolarization block (or excitation block; Izhikevich, 2007), which is indicated by segment number 1 (see *ψ*; Fig. 14*B*). The NS is indicated by segment number 2. Therefore, the transitions between ictal states and NSs reduce to a periodic transition between DB and NS, which are both slow manifolds.

**Figure 15.**
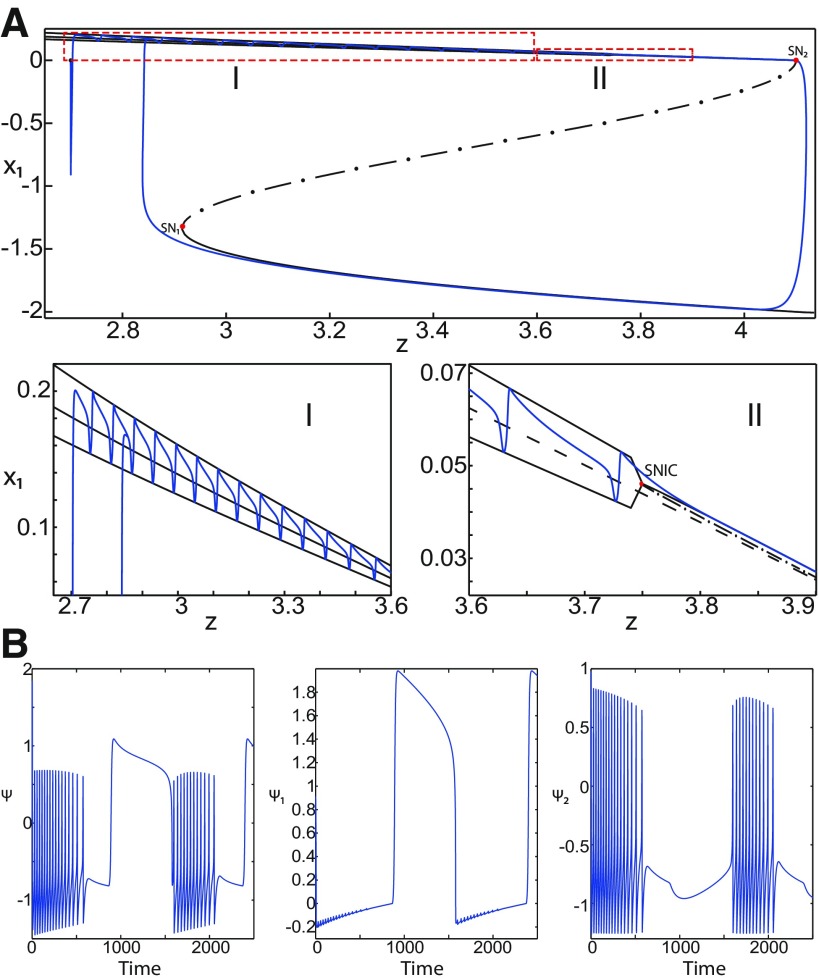
***A***, Top, Bifurcation diagram (*z*, *x*_1_) of the Epileptor model. Parts I and II are zoomed on the bottom. The transitions between the *Z*-upper and *Z*-lower branches occur through a fold/circle bifurcation. ***B***, Deterministic time series of the Epileptor system *ψ*, subsystem 1 *ψ*_1_, and subsystem 2 *ψ*_2_. Parameters are: *m* = −8, *x*_0_ = −1.6, *I*_ext2_ = 0.45, *r * = * *0.0005, I.C = [0 −5 2.7 0 0 0.01], and *T_s_* = [0:0.001: 1600].

The DB occurs when $I_{ext_{2}}=0$ but not when $I_{ext_{2}}=0.45$. To see this, we plot a (*z*, *x*_1_) bifurcation diagram for *m* = −8 ($I_{ext_{2}}=0.45$) in Figure 15*A*, which is with a *Z*-shaped curve. *Z*-lower (solid), *Z*-middle (dash-dotted), and *Z*-upper (dashed) branches consist of stable nodes, saddles, and unstable foci, respectively. Above the *Z*-curve, lower branch consists of saddles and upper branch consists of stable nodes (Fig. 15*A*, inset II). Decreasing *z*, lower and upper branches collide in a SNIC bifurcation. Let *x*_0_ = −1.6, the equilibrium point is a saddle. When a trajectory is at the *Z*-lower branch, the stable node disappears as *z* decreases through a saddle-node bifurcation SN_1_ and the trajectory switches to the *Z*-upper branch exhibiting an oscillatory solution (Fig. 15*A*, inset I), which terminates as *z* increases in a SNIC bifurcation (Fig. 15*A*, inset II). Then, the trajectory exhibits a nonoscillatory solution (Fig. 15*A*, inset II), which terminates as *z* increases in a saddle-node bifurcation, SN_2_ (Fig. 15*A*), and the Epileptor switches to the *Z*-lower branch. The transitions between *Z*-lower and *Z*-upper branches occur through a fold/circle bifurcation. The system is bistable on [SN_1_, SN_2_]. Time series of the Epileptor model *ψ*, subsystem 1 *ψ*_1_, and subsystem 2 *ψ*_2_ are plotted in Figure 15*B*. The Epileptor exhibits fast discharges in the ictal state (see *ψ*), and hence does not enter into a DB.

To understand why a DB occurs for $I_{ext_{2}}=0$ and not for $I_{ext_{2}}=0.45$, we analyze the [SN_1_, SN_2_] interval, on which the Epileptor model is bistable. We determine the branches of the (*z*, *x*_1_) curve existing on this interval ($\forall x_{1}\geq 0$) for $I_{ext_{2}}=0.45$ and $I_{ext_{2}}=0$. Distinct branches coexist on [SN_1_, SN_2_] depending on *m*:

- When *m* = 0, then three branches coexist on [SN_1_, SN_2_], consisting of stable nodes, saddles, and unstable foci for $I_{ext_{2}}=0.45$ (Fig. 5), and they consist of saddles and unstable foci for $I_{ext_{2}}=0$ (Fig. 6).
- When *m* = −1, then three branches coexist on [SN_1_, SN_2_], which consist of stable foci, saddles, and unstable foci for $I_{ext_{2}}=0.45$ (figure not shown) and $I_{ext_{2}}=0$ (Fig. 8).
- When *m* = −8, then three branches coexist for $I_{ext_{2}}=0.45$, which consist of stable nodes, saddles, and unstable foci (Fig. 15*A*). For $I_{ext_{2}}=0$, one branch exists which consists of stable nodes (Fig. 14*A*).

Therefore, a DB occurs when *m* = −8 and $I_{ext_{2}}=0$ because one branch exists on [SN_1_, SN_2_] (∀x≥0), which consists of stable nodes. The SLE attractor is characterized by transitions between *Z*-lower and *Z*-upper branches. The *Z*-lower branch consists of stable nodes, which are the equilibrium points of the NS. When *m* = −8 and $I_{ext_{2}}=0$, the *Z*-upper branch consists of stable nodes, which are the equilibrium points of the DB. Hence, the SLE attractor reduces as *m* further decreases to a periodic switch between DB and NS.

#### Stabilizing equilibrium points in the Epileptor model

The transitions between ictal and normal states of the SLE attractor occur when the equilibrium point is a saddle (*x*_0_ = −1.6). When *x*_0_ increases, the Epileptor stabilizes in the ictal (nonoscillatory) state, and when *x*_0_ decreases, the Epileptor model stabilizes in the normal state.

##### Stabilizing the equilibrium point of the nonoscillatory state

**Figure 16.**
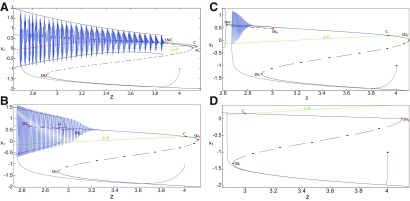
Stabilizing the equilibrium point of the nonoscillatory state and DB. The Epileptor model remains in the ictal nonoscillatory state after a fast discharges period. We plot a (*z*, *x*_1_) bifurcation diagram for different values of *m*, *x*_0_ and *I*_ext2_. ***A–C***, *I*_ext2_ = 0.45, *m * = * *0 and *x*_0_ = −0.9 (***A***), *I*_ext2_ = 0, *m* = −0.5, and *x*_0_ = −0.8 (***B***), and *I*_ext2_ = 0, *m* = −1, and *x*_0_ = −0.8 (***C***). The equilibrium point C is a stable focus for ***A–C***. The Epileptor stabilizes its equilibrium point ***C*** after transient seizure-like fast discharges, which disappear through a SNIC bifurcation (***A***) and a through Hopf bifurcation (***B***). The branches description for ***A–C*** is provided in Figures 5, 7, and 8, respectively. ***D***, We plot a (*z*, *x*_1_) bifurcation diagram with respect to *z* for *m* = −8 and *I*_ext2_ = 0. The *Z*-upper (dashed), *Z*-middle (dash-dotted), and *Z*-lower (solid) branches consist of stable nodes, saddles, and stable nodes, respectively. Let *x*_0_ = −0.6, the *z*-nullcline ($\dot{z}=0$) intersects the *Z*-upper branch at *C*, which is a stable node. The Epileptor model remains in DB after a transient NS. For all deterministic simulations: I.C = [−1 −5 4 0 0 0.01]. *r * = * *0.00095 and *T_s_* = [0:0.001:2000] for ***A***; *r * = * *0.002 and *T_s_* = [0:0.001:2000] for ***B***; *r * = * *0.001 and *T_s_* = [0:0.001:2000] for ***C***; and *r * = * *0.00005 and *T_s_* = [0:0.001:3000] for ***D***.

During the ictal state, epileptiform fast discharges (oscillatory state) disappear through three bifurcation types depending on *m* and $I_{ext_{2}}$: a homoclinic bifurcation (Figs. 4, 6), a SNIC bifurcation (Fig. 5), or a Hopf bifurcation (Fig. 7). When the equilibrium point is a saddle (*x*_0_ = −1.6), the Epileptor switches differently to the normal state according to the bifurcation type: homoclinic, SNIC, or Hopf. The Epileptor switches to the normal state just after a homoclinic bifurcation, and remains in the nonoscillatory state after SNIC and Hopf bifurcations, thereby exhibiting a nonoscillatory solution. The latter disappears as *z* increases through a saddle-node bifurcation, SN_2_, and then the Epileptor switches to the normal state. When increasing *x*_0_, the stability of the equilibrium point changes, and the Epileptor remains in the nonoscillatory state. We illustrate this scenario in Figure 16*A–C*.

Let *m * = * *0 and *x*_0_ = −0.9 (Fig. 16*A*), the *z*-nullcline intersects three branches of the (*z*, *x*_1_) curve: lower and upper (solid) branches above the *Z*-curve, and the *Z*-upper (dashed) branch. The *z*-nullcline intersects the upper branch above *Z* at *C*. Then three equilibrium points coexist: an unstable focus, a saddle, and a stable focus (C). When a trajectory is at the *Z*-lower branch, the stable node disappears as *z* decreases through a saddle-node bifurcation, SN_1_, and the trajectory switches to the *Z*-upper branch exhibiting an oscillatory solution (fast discharges), which terminates as *z* increases in a SNIC bifurcation. Then, the trajectory exhibits a nonoscillatory solution at the upper (solid) branch above the *Z*-curve, and continues to *C* at which *z* stabilizes. Therefore, the transition to the *Z*-lower branch does not occur and the Epileptor remains in the nonoscillatory state. We plot this scenario in a (*Y*, *X*, *z*) phase space (see Fig. 28*A*, top trajectory). Corresponding time series are plotted in Figure 28*B*. We add numbers to identify trajectory segments. We indicate the normal state by (1), fast discharges by (2), and the final state by (3), which corresponds to the equilibrium point of the nonoscillatory state. Here, the NS exists but not the equilibrium point. Hence, after a transient normal state, the Epileptor exhibits fast discharges which disappear in a SNIC bifurcation, and then remains in the nonoscillatory state.

Let *m* = −0.5 and *x*_0_ = −0.8 (Fig. 16*B*), the *z*-nullcline intersects the *Z*-upper (solid) sub-branch at *C* which is a stable focus. When a trajectory is at the *Z*-lower branch, the stable node disappears as *z* decreases through a saddle-node bifurcation SN_1_ and the trajectory switches to the *Z*-upper (dashed) sub-branch exhibiting an oscillatory solution (fast discharges), which terminates as *z* increases in a Hopf bifurcation, *H*. Then, the trajectory exhibits a nonoscillatory solution at the *Z*-upper (solid) sub-branch, which consists of stable foci, and continues to *C* at which *z* stabilizes. The transition to the *Z*-lower branch does not occur, and the Epileptor remains in the nonoscillatory state. We plot this scenario in a (*Y*, *X*, *z*) phase space (see Fig. 29A, top trajectory). Corresponding time series are plotted in Figure 29*B*. We indicate the normal state by (1), fast discharges by (2), and the final state by (3), which corresponds to the equilibrium point of the nonoscillatory state. After a transient normal state, the Epileptor exhibits fast discharges which disappear in a Hopf bifurcation, and then remains in the nonoscillatory state.

Let *m* = −1, the SLE attractor reduces to transitions between nonoscillatory and normal states when *x*_0_ = −1.6, which occur through a fold/fold bifurcation (Fig. 8). When increasing *x*_0_, the Epileptor model stabilizes its stable focus, which corresponds to the equilibrium point of the nonoscillatory state. We illustrate this case in Figure 16*C*. Let *x*_0_ = −0.8, the *z*-nullcline intersects the *Z*-upper branch at *C*, which is a stable focus. When a trajectory is at the *Z*-lower branch, the stable node disappears as *z* decreases through a saddle-node bifurcation, SN_1_, and the trajectory switches to the *Z*-upper branch exhibiting a nonoscillatory solution. Then, the trajectory continues to *C* at which *z* stabilizes. The transition to the *Z*-lower branch does not occur and the Epileptor remains in the nonoscillatory state. We plot this scenario in a (*Y*, *X*, *z*) phase space (see Fig. 30*A*, top trajectory). Corresponding time series are plotted in Figure 30*B*. We indicate the normal state by (1) and the final state by (3), which corresponds to the equilibrium point of the nonoscillatory state. The imaginary part of the eigenvalues of *C* is responsible for the oscillations (2) around the stable focus. After a transient normal state, the Epileptor spirals into the equilibrium point of the nonoscillatory state exhibiting damped oscillations, and does not re-enter the normal state.

##### Stabilizing the equilibrium point of the DB

Figure 14 shows that the SLE attractor reduces to a periodic switch between DB and NS when further decreasing *m* ($I_{ext_{2}}=0$). The equilibrium point is a saddle (*x*_0_ = −1.6). When increasing *x*_0_, the Epileptor model stabilizes its stable node, which corresponds to the equilibrium point of DB. To see this, we plot a (*z*, *x*_1_) bifurcation diagram when *x*_0_ = −0.8 in Figure 16*D*. The *z*-nullcline intersects the *Z*-upper (solid) branch at *C*, which is a stable node. When a trajectory is at the *Z*-lower branch, the stable node disappears as *z* decreases through a saddle-node bifurcation SN_1_ and the trajectory switches to the *Z*-upper branch and enters into a DB. The trajectory continues as *z* increases to *C* at which *z* stabilizes. Thus, the transition to the *Z*-lower branch does not occur and the Epileptor remains in DB. We plot this scenario in a (*Y*, *X*, *z*) phase space (see Fig. 27*A*, top trajectory). Corresponding time series are plotted in Figure 27*B*. We indicate the normal state by (2) and the final state by (4), which corresponds to the equilibrium point of DB. Then, after a transient NS, the Epileptor enters into DB and then remains in it.

##### Stabilizing the equilibrium point of the NS

**Figure 17.**
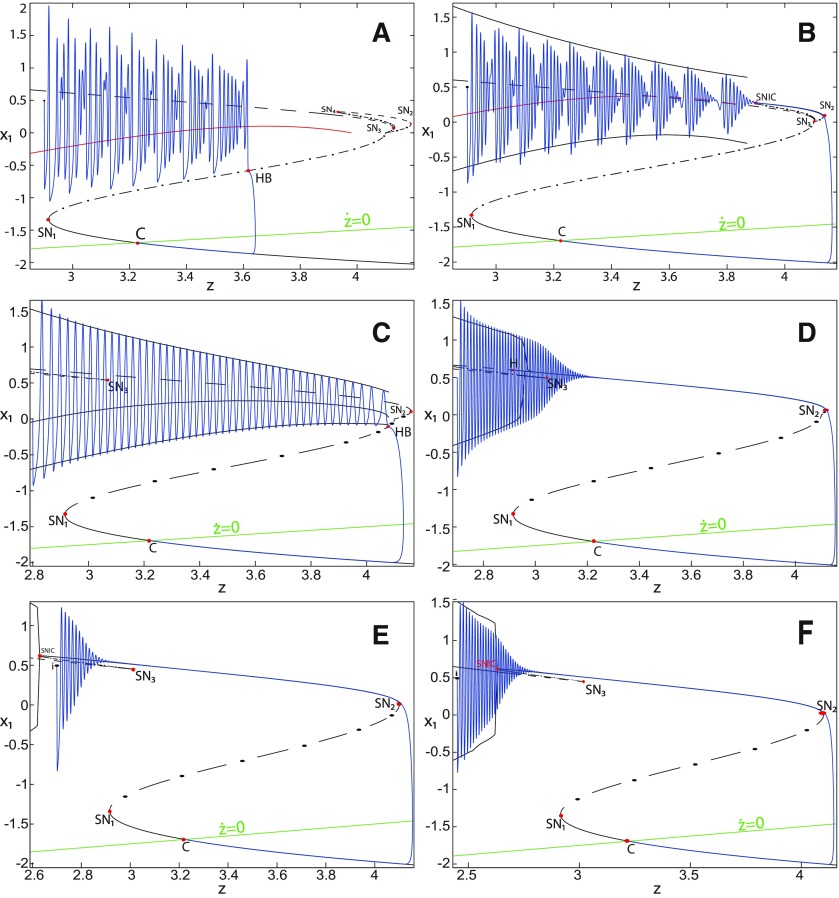
Stabilizing the equilibrium point of the NS. We plot a (*z*, *x*_1_) bifurcation diagram with respect to *z*, as *m* and *I*_ext2_ vary (*x*_0_ = −2.5). ***A–F***, *m * = * *0.5 and *I*_ext2_ = 0.45 (***A***), *m * = * *0 and *I*_ext2_ = 0.45 (***B***), *m * = * *0 and *I*_ext2_ = 0 (***C***), *m* = −0.5 and *I*_ext2_ = 0 (***D***), and *m* = −1 and *I*_ext2_ = 0 (***E***, ***F***). Branches of the (*z*, *x*_1_) curve in ***A*** are the same as in Figure 4. Branches of the (*z*, *x*_1_) curve in ***B*** are the same as in Figure 5. Branches of the (*z*, *x*_1_) curve in ***C*** are the same as in Figure 6. Branches of the (*z*, *x*_1_) curve in ***D*** are the same as in Figure 7. Branches of the (*z*, *x*_1_) curve in ***E*** and ***F*** are the same as in Figure 8. ***A*–*F***, The *z*-nullcline ($\dot{z}=0$) intersects the *Z*-lower branch at *C*, which is a stable node. The Epileptor model remains in NS after a transient ictal state. For all (deterministic) simulations *T_s_* = [0:0.001:4500]. I.C = [0.5 −5 2.9 0 0 0.01] and *r * = * *0.00053 for ***A***; I.C = [0.5 −5 2.9 0 0 0.01] and *r * = * *0.00035 for ***B***; I.C = [0 −5 2.8 0 0 0.01] and *r * = * *0.0007 for ***C***; I.C = [0.5 −5 2.7 0 0 0.01] and *r * = * *0.00035 for ***D***; I.C = [0.5 −5 2.7 0 0 0.01] and *r * = * *0.0005 for ***E***; and I.C = [0.5 −5 2.45 0 0 0.01] and *r * = * *0.0004 for ***F***.

When the equilibrium point is a saddle (*x*_0_ = −1.6), the normal state of the SLE attractor disappears through a saddle-node bifurcation SN_1_. When decreasing *x*_0_, the Epileptor stabilizes its stable node, which is the equilibrium point of the normal state. To see this, we plot a (*z*, *x*_1_) bifurcation diagram when *x*_0_ = −2.5 in Figure 17. The *z*-nullcline intersects the *Z*-lower branch at *C*, which corresponds to the equilibrium point of the normal state. The ictal state (*Z*-upper branch) exists but not the equilibrium point, hence the Epileptor remains in the normal state, at *C* which is a stable node.

When $I_{ext_{2}}=0.45$, the transition from ictal to normal states occurs according to two scenarios, depending on *m*.

- *m* = 0.5 (Fig. 17*A*): When a trajectory is at the *Z*-upper branch (dashed), the oscillatory solution terminates as *z* increases in an HB bifurcation and the trajectory switches to the *Z*-lower branch.
- *m* = 0 (Fig. 17*B*): When a trajectory is at the *Z*-upper branch (dashed), the oscillatory solution terminates as *z* increases in a SNIC bifurcation, then the trajectory exhibits a nonoscillatory solution on the upper branch above the *Z*-curve, which terminates as *z* increases in a saddle-node bifurcation SN_2_. Then the trajectory switches to the *Z*-lower branch.

When $I_{ext_{2}}=0$, the transition from ictal to normal states occurs according to four scenarios, depending on *m*.

- For *m* ≥ 0 (Fig. 17*C*): The trajectory exhibits an oscillatory solution on the *Z*-upper (dashed) branch, which terminates as *z* increases in a homoclinic bifurcation HB, and then it switches to the *Z*-lower branch.
- For *m* = −0.5 (Fig. 17*D*): The trajectory exhibits an oscillatory solution on the *Z*-upper sub-(dashed) branch, which terminates as *z* increases in a Hopf bifurcation *H*. Then the trajectory exhibits a nonoscillatory solution on the *Z*-upper (solid) sub-branch, which terminates as *z* increases in a saddle-node bifurcation SN_2_, and the trajectory switches to the *Z*-lower branch.
- For *m* ≤ −1, when a trajectory starts at *i* with z(SNIC)<z(i) (third scenario), then it exhibits a nonoscillatory solution on the *Z*-upper branch, which terminates as *z* increases in a saddle-node bifurcation, SN_2_. Then the trajectory switches to the *Z*-lower branch (Fig. 17*E*). When a trajectory starts at *i* with z(i)<z(SNIC) (fourth scenario), then it exhibits an oscillatory solution, which terminates as *z* increases in a SNIC bifurcation. Then, the trajectory is at the *Z*-upper branch exhibiting a nonoscillatory solution, which terminates as *z* increases in a saddle-node bifurcation SN_2_. Then, the trajectory switches to the *Z*-lower branch (Fig. 17*F*).

For all *m*, when the trajectory is at the *Z*-lower branch, which consists of stable nodes, it continues as *z* decreases to *C*, at which *z* stabilizes. Therefore, the transition to the *Z*-upper branch does not occur and the Epileptor remains in the NS.

#### Coexistence in the Epileptor model

Figure 11, *A* and *B*, shows the evolution of periodic orbits as *x*_0_ varies. The periodic orbits LC and S disappear as *x*_0_ decreases through a SNPO bifurcation. The dynamics of SLC when it exists, depends on the equilibrium points stability. Figure 4 shows that the *z*-nullcline and the $<x_{1}>$-curve intersect at two points: LC and S. Depending on initial conditions, trajectories either converge to LC or exhibit SLEs. We identify seven types of coexisting attractors in the Epileptor model.

##### Coexistence of LC and seizures (SLEs)

**Figure 18.**
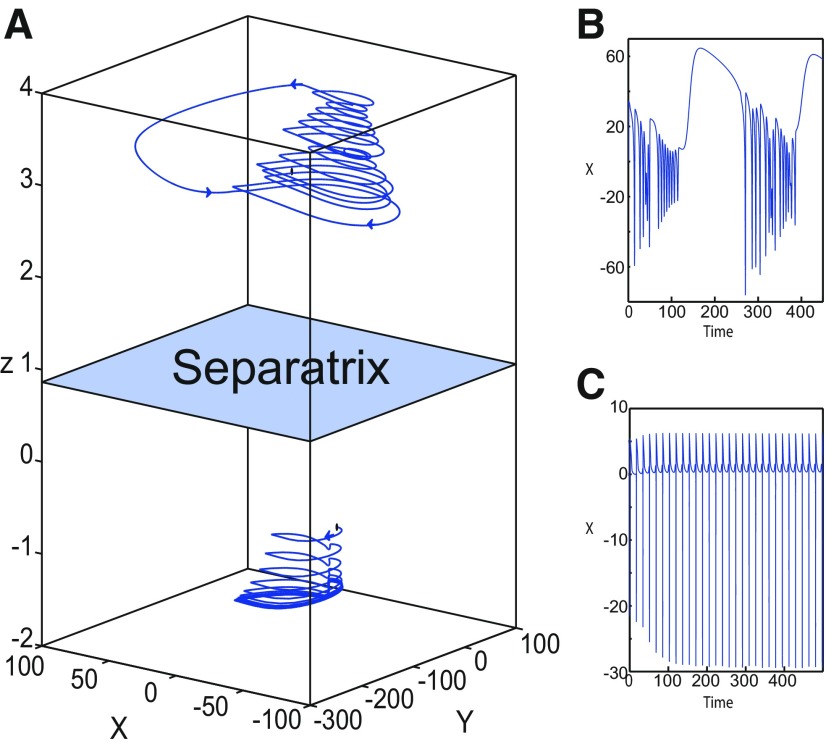
***A***, Coexistence of SLEs and RSE. The simulations are performed without noise. SLEs and a stable LC coexist for *m * = * *0.5 and *x*_0_ = −1.6 ($I_{ext_{2}}=0.45$). SLEs occur through a fold/homoclinic bifurcation. The arrows indicate the direction of trajectories. For easier visualization, we plot generalized coordinates (*X*, *Y*) corresponding to ($-35x_{1}+x_{2},\;15y_{1}$) for seizures (top) and to ($-0.5x_{1}+x_{2},\;0.1y_{1}$) for LC (bottom). LC is characteristic of RSE. ***B***, ***C***, Time series of SLEs (***B***) and LC (***C***). Parameter settings correspond to region VIII in Figure 31 and to region 13 in Figure 32. ***A***, Top, I.C = [0 −5 3 0 0 0.01] and *r * = * *0.035. ***A***, Bottom, I.C = [0 −5 −1 0 0 0.01] and *r * = * *0.035. The coexistence of LC and separatrix (*S*) can be found in area II [Fig. 12A].

Depending on *m* and $I_{ext_{2}}$, transitions between ictal and normal states, which characterize the SLE attractor, occur through a fold/homoclinic bifurcation, a fold/circle bifurcation, or a fold/Hopf bifurcation. We describe for each bifurcation type the coexistence of LC and the SLE attractor:

*A fold/homoclinic bifurcation:* We plot two trajectories in a (*z*, *x*_1_) bifurcation diagram for *m * = * *0.5 (Fig. 4). The equilibrium point is a saddle (*x*_0_ = −1.6). The behavior of the first trajectory (right) corresponds to SLEs, which occur through a fold/homoclinic bifurcation. The *Z*-middle branch acts as a separatrix between ictal and normal states. The second trajectory (left) converges to LC. The saddle periodic orbit (S) separates LC and the SLE attractor. We plot LC, SLEs, and S in a (*Y*, *X*, *z*) phase space (Fig. 18*A*). SLEs and LC coexist and the separatrix between them corresponds to the saddle periodic orbit (S). SLEs lock in LC when *z* declines to below baseline shift (Fig. 18*A*). Time series are plotted in Figure 18*B* for SLEs and in Figure 18*C* for LC.

**Figure 19.**
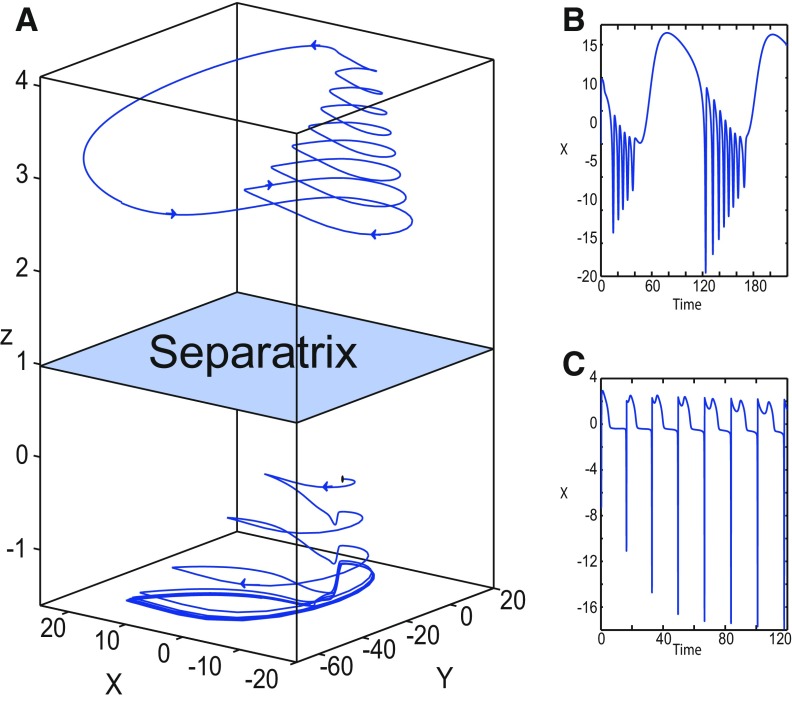
***A***, Coexistence of SLEs and RSE. The simulations are performed without noise. SLEs and a stable LC coexist for *m * = * *0 and *x*_0_ = −1.6 ($I_{ext_{2}}=0$). SLEs occur through a fold/homoclinic bifurcation. The arrows indicate the direction of trajectories. For easier visualization, we plot generalized coordinates (*X, Y*) corresponding to ($-10x_{1}+x_{2},\;5y_{1}$) for seizures (top) and to ($-0.3x_{1}+x_{2},\;0.06y_{1}$) for LC (bottom). LC is characteristic of RSE. ***B***, ***C***, Time series of SLEs (***B***) and LC (***C***). Parameter settings correspond to region X in Figure 34 and to region 15 in Figure 35. ***A***, Top, I.C = [0 −5 3 0 0 0.01], *T_s_* = [0 220], and *r * = * *0.01. ***A***, Bottom, I.C = [4 −1 −0.5 0 0 0.01], *T_s_* = [0 120], and *r * = * *0.01. The coexistence of LC and *S* can be found in area II (Fig. 12*B*).

Figure 6 shows that SLEs occur through a fold/homoclinic bifurcation when $I_{ext_{2}}=0$. Parameter settings in Figure 6 correspond to area II in Figure 12*B*. Hence, SLEs and LC coexist and are separated by S. Only the SLE attractor is plotted in Figure 6. We plot LC, SLEs, and S in a (*Y*, *X*, *z*) phase space (Fig. 19*A*). SLEs and LC coexist and are separated by a separatrix, which corresponds to the saddle periodic orbit (S). Time series are plotted in Figure 19*B* for SLEs and in Figure 19*C* for LC.

**Figure 20.**
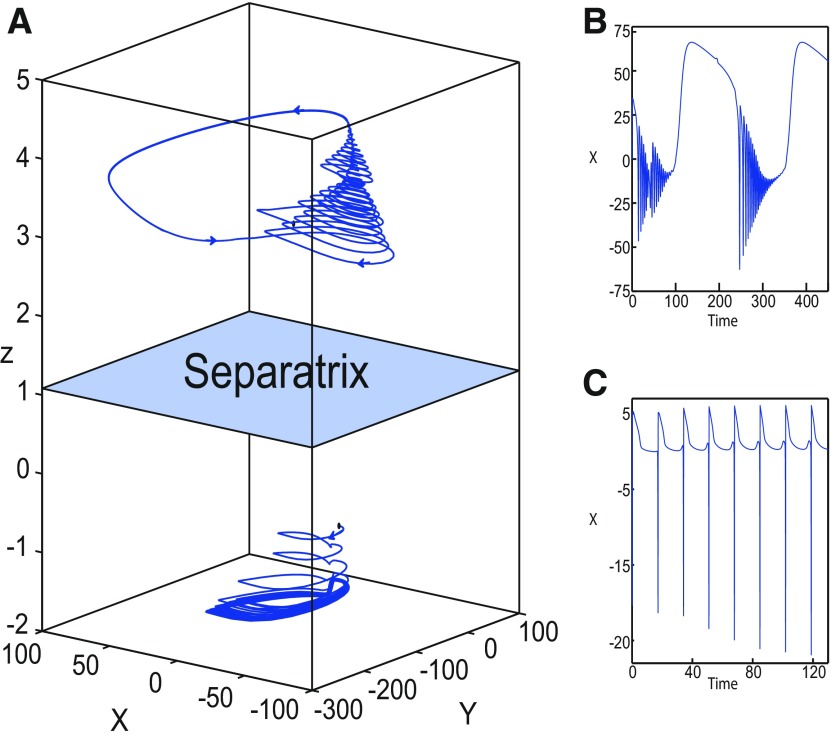
***A***, Coexistence of SLEs and RSE. The simulations are performed without noise. SLEs and a stable LC coexist for *m * = * *0 and *x*_0_ = −1.6 ($I_{ext_{2}}=0.45$). SLEs occur through a fold/circle bifurcation. The arrows indicate the direction of trajectories. For easier visualization, we plot generalized coordinates (*X, Y*) corresponding to ($-35x_{1}+x_{2},\;15y_{1}$) for seizures (top) and to ($-0.5x_{1}+x_{2},\;0.1y_{1}$) for LC (bottom). LC is characteristic of RSE. ***B***, ***C***, Time series of SLEs (***B***) and LC (***C***). Parameter settings correspond to region VII in Figure 31 and to region 12 in Figure 32. ***A***, Top, I.C = [0 −5 3 0 0 0.01], *T_s_* = [0 220], and *r * = * *0.01. ***A***, Bottom, I.C = [4 −1 −0.5 0 0 0.01], *T_s_* = [0 120], and *r * = * *0.01. The coexistence of LC and *S* can be found in area II (Fig. 12*A*).

*A fold/circle bifurcation:* We plot two trajectories in a (*z*, *x*_1_) bifurcation diagram (Fig. 5). The first trajectory (right) exhibits SLEs, and the second one (left) converges to LC. The equilibrium point is a saddle (*x*_0_ = −1.6). The transitions between ictal and normal states occur through a fold/circle bifurcation. Then SLEs and LC coexist and are separated by a saddle periodic orbit (S). We plot LC, SLEs, and S in a (*Y*, *X*, *z*) phase space (Fig. 20*A*). SLEs and LC coexist and are separated by a saddle periodic orbit (S), which is the separatrix. Time series are plotted in Figure 20*B* for SLEs and in Figure 20*C* for LC.

**Figure 21.**
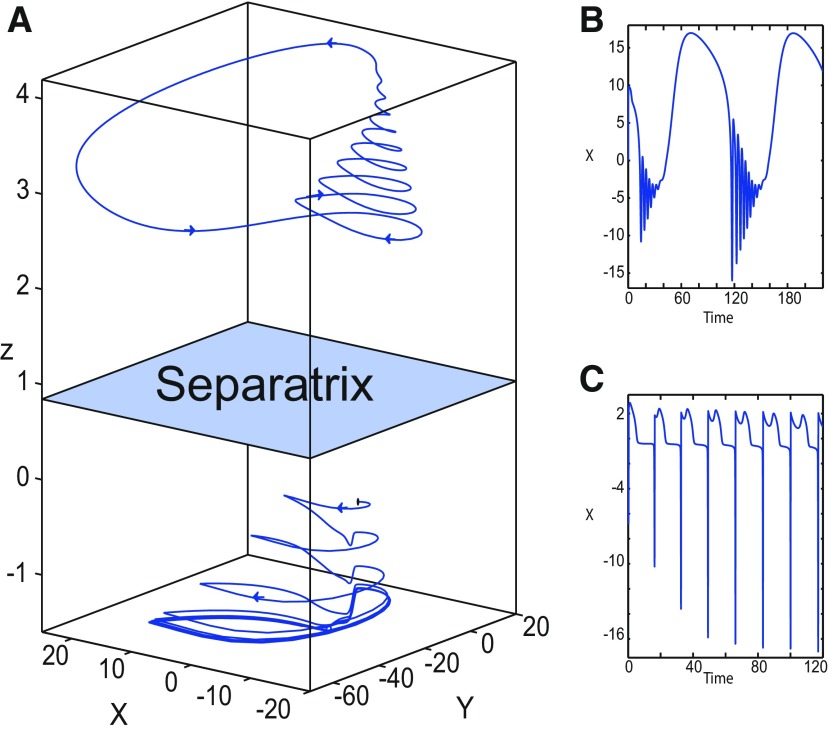
***A***, Coexistence of SLEs and RSE. The simulations are performed without noise. SLEs and a stable LC coexist for *m* = −0.5 and *x*_0_ = −1.6 ($I_{ext_{2}}=0$). SLEs occur through a fold/Hopf bifurcation. The arrows indicate the direction of trajectories. For easier visualization, we plot generalized coordinates (*X, Y*) corresponding to ($-10x_{1}+x_{2},\;5y_{1}$) for seizures (top) and to ($-0.3x_{1}+x_{2},\;0.06y_{1}$) for LC (bottom). LC is characteristic of RSE. ***B***, ***C***, Time series of SLEs (***B***) and LC (***C***). Parameter settings correspond to region IX in Figure 34 and to region 14 in Figure 35. ***A***, Top, I.C = [0 −5 3 0 0 0.01], *T_s_* = [0 220], and *r * = * *0.01. ***A***, Bottom, I.C = [4 −1 −0.5 0 0 0.01], *T_s_* = [0 120], and *r * = * *0.01. The coexistence of LC and *S* can be found in area II [Fig. 12*B*].

*A fold/Hopf bifurcation:* Figure 7 shows that SLEs occur through a fold/Hopf bifurcation when m=−0.5 ($I_{ext_{2}}=0$). The equilibrium point is a saddle (*x*_0_ = −1.6). Parameter settings in Figure 7 correspond to area II in Figure 12*B*. Then SLEs and LC coexist and are separated by S. Figure 7 only shows the SLE attractor. We plot LC, SLEs, and S in a (*Y*, *X*, *z*) phase space (Fig. 21*A*). SLEs and LC coexist and the separatrix between them corresponds to a saddle periodic orbit (S). Time series are plotted in Figure 21*B* for SLEs and in Figure 21*C* for LC.

##### Coexistence of LC and a periodic switch between nonoscillatory state and NS

**Figure 22.**
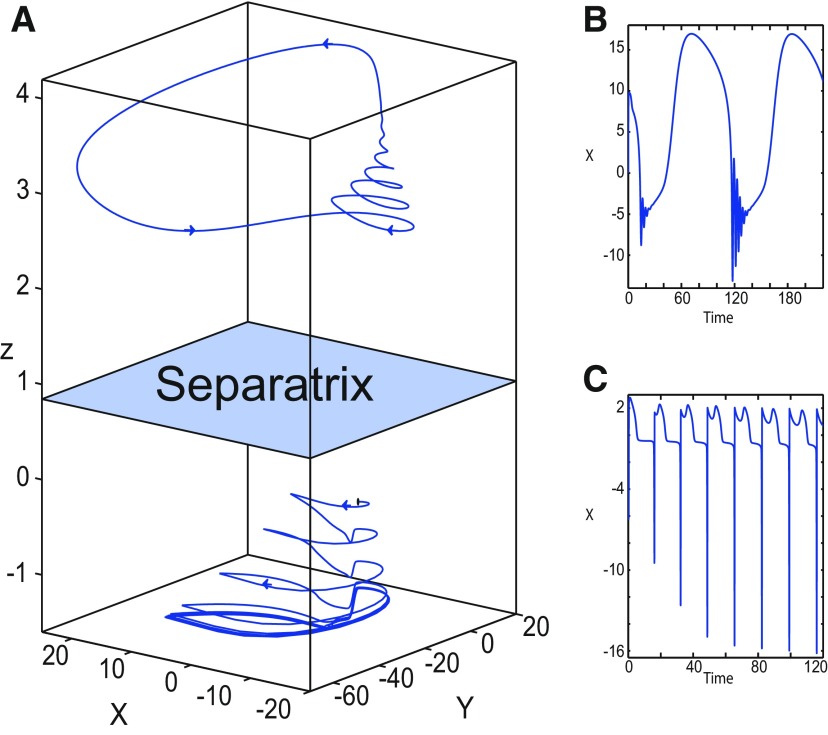
***A***, Coexistence of RSE and the periodic switch between nonoscillatory state and NS. The simulations are performed without noise. Here, the SLE attractor reduces to the periodic switch between nonoscillatory state and NS, which coexists with a stable LC for *m* = −1 and $x_{0}=-1.6$ ($I_{ext_{2}}=0$). This periodic switch occurs through a fold/fold bifurcation. The arrows indicate the direction of trajectories. For easier visualization, we plot generalized coordinates (*X, Y*) corresponding to ($-10x_{1}+x_{2},\;5y_{1}$) for the periodic switch between nonoscillatory state and NS (top) and to ($-0.3x_{1}+x_{2},\;0.06y_{1}$) for LC (bottom). LC is characteristic of RSE. ***B***, ***C***, Time series of this periodic switch (***B***) and LC (***C***). Parameter settings correspond to region VIII in Figure 34 and to region 13 in Figure 35. ***A***, Top, I.C = [0 −5 3 0 0 0.01], *T_s_* = [0 220], and *r * = * *0.01. ***A***, Bottom, I.C = [4 −1 0.5 0 0 0.01], *T_s_* = [0 120], and *r * = * *0.01. The coexistence of LC and *S* can be found in area II (Fig. 12*B*).

Figure 8 shows that the SLE attractor reduces to a periodic switch between nonoscillatory state and NS, which occurs through a fold/fold bifurcation when *m* = −1 ($I_{ext_{2}}=0$). The equilibrium point is a saddle (*x*_0_ = −1.6). Parameter settings in Figure 8 correspond to area II in Figure 12*B*. Then LC and the periodic switch between nonoscillatory state and NS coexist, and are separated by S. Only the periodic switch between nonoscillatory state and NS is plotted in Figure 8. We plot LC, this periodic switch, and S in a (*Y*, *X*, *z*) phase space (Fig. 22*A*). LC and the periodic switch between nonoscillatory state and NS coexist and the separatrix between them corresponds to a saddle periodic orbit (S). Time series are plotted in Figure 22*B* for this periodic switch and in Figure 22*C* for LC.

##### Coexistence of two periodic orbits: LC and SLC

**Figure 23.**
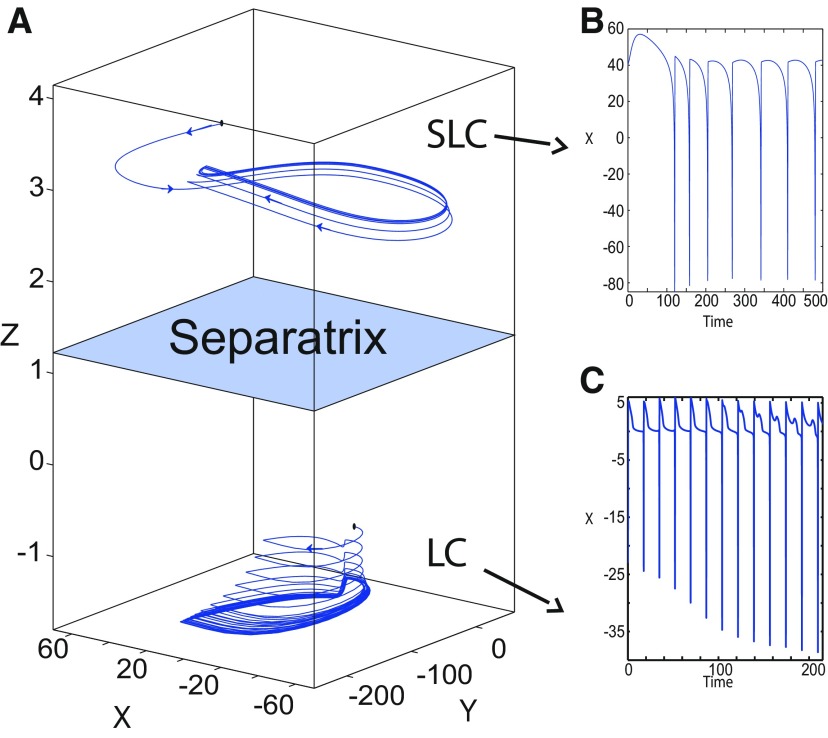
***A***, Coexistence of SLC and RSE. The simulations are performed without noise. SLC and a stable LC coexist for *m * = * *1 and $x_{0}=-1.8$ ($I_{ext_{2}}=0$). The equilibrium point is a saddle. The arrows indicate the direction of trajectories. For easier visualization, we plot generalized coordinates (*X*, *Y*) corresponding to ($-35x_{1}+x_{2},\;15y_{1}$) for SLC (top) and to ($-0.5x_{1}+x_{2},\;0.1y_{1}$) for LC (bottom). LC is characteristic of RSE. ***B***, ***C***, Time series of SLC (***B***) and LC (***C***). Parameter settings correspond to region II in Figure 34 and to region 17 in Figure 35. ***A***, Top, I.C = [−1.15 −5 3.4 0 0 0.01], *T_s_* = [0 500], and *r * = * *0.0035. ***A***, Bottom, I.C = [10 −5 −1 0 0 0.01], *T_s_* = [0 500], and *r * = * *0.0035.The coexistence of LC, *S*, and SLC can be found in area III [Fig. 12*B*].

**Figure 24.**
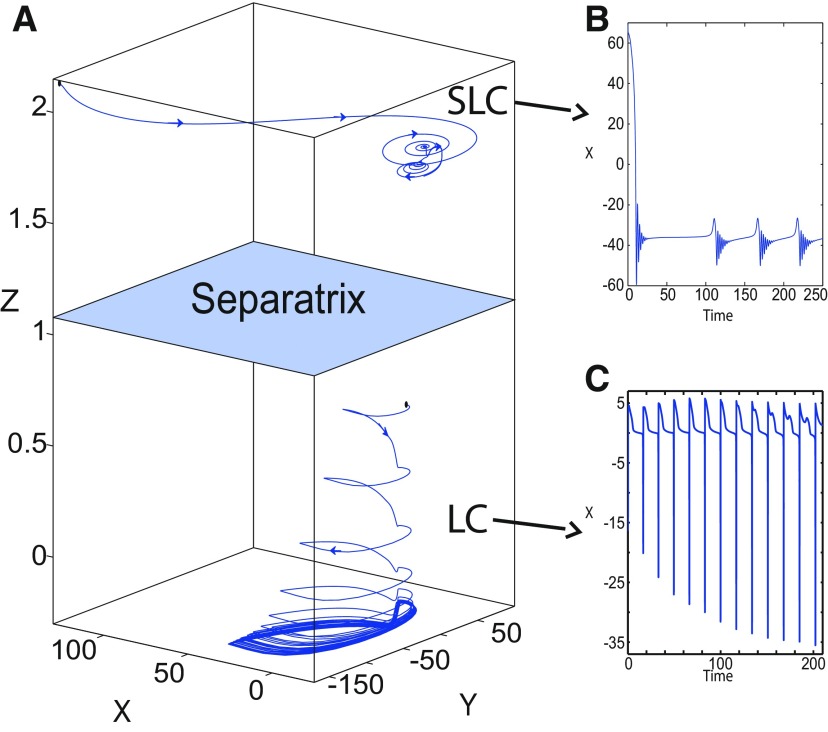
***A***, Coexistence of SLC and RSE. The simulations are performed without noise. SLC and a stable LC coexist for *m* = −2 and $x_{0}=0$ ($I_{ext_{2}}=0$). The equilibrium point is an unstable focus. The arrows indicate the direction of trajectories. For easier visualization, we plot generalized coordinates (*X*, *Y*, *Z*) corresponding to ($-60x_{1}+x_{2},\;60y_{1},\;z-0.5$) for SLC (top) and to ($-0.5x_{1}+x_{2},\;0.1y_{1},\;z+1.7$) for LC (bottom). LC is characteristic of RSE. ***B***, ***C***, Time series of SLC (***B***) and LC (***C***). Parameter settings correspond to region II in Figure 34 and to region 2 in Figure 35. ***A***, Top, I.C = [−1.15 −5 2.9 0 0 0.01], $T_{s}=[0:0.01:250]$, and *r * = * *0.007. ***A***, Bottom, I.C = [10 −5 −1 0 0 0.01], *T_s_* = [0 500], and *r * = * *0.002. The coexistence of LC, *S*, and SLC can be found in area III [Fig. 12*B*].

SLC is a stable periodic orbit with a small amplitude. When $I_{ext_{2}}=0$, then SLC, S, and LC coexist in area III (Fig. 12*B*). Figure 13 shows that the *z*-nullcline intersects the $<x_{1}>$-curve at three points: LC, S, and SLC. Here, we plot the SLC behavior only, which depends on the stability of the equilibrium point. SLC and LC coexist in both cases: when the equilibrium point is a saddle (Fig. 13*A*) and when it is an unstable focus (Fig. 13*B*). We plot SLC, LC, and S in a (*Y*, *X*, *Z*) phase space (Figs. 23, 24). LC and SLC coexist and are separated by a saddle periodic orbit (S), which corresponds to the separatrix. When the equilibrium point is a saddle, the coexistence of LC and SLC is illustrated in Figure 23*A*. Time series are plotted in Figure 23*B* for SLC and in Figure 23*C* for LC. When the equilibrium point is an unstable focus, the coexistence of LC and SLC is illustrated in Figure 24*A*. Time series are plotted in Figure 24*B* for SLC and in Figure 24*C* for LC.

##### Coexistence of LC and a periodic switch between DB and NS

**Figure 25.**
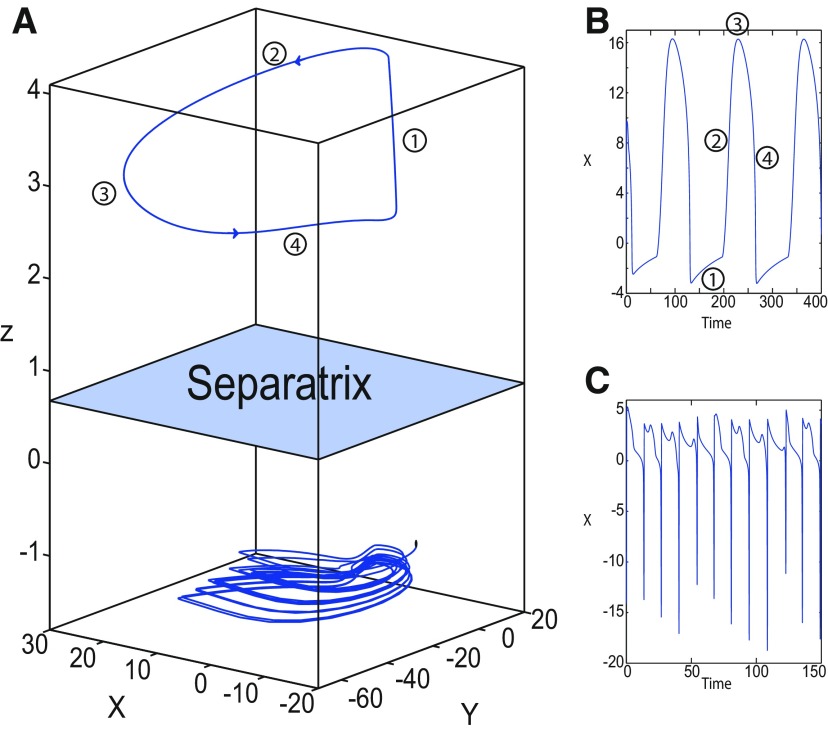
***A***, Coexistence of RSE and a periodic switch between DB and NS. The simulations are performed without noise. Here, the SLE attractor reduces to the periodic switch between DB and NS, which coexists with a stable LC for *m* = −8 and *x*_0_ = −1.4 ($I_{ext_{2}}=0$). Trajectory segments are numbered in ***A*** and ***B***. DB corresponds to the segment 1, and the NS to the segment 3. The arrows indicate the direction of trajectories. For easier visualization, we plot generalized coordinates (*X*, *Y*) corresponding to ($-10x_{1}+x_{2},\;5y_{1}$) for the periodic switch between DB and NS (top) and to ($-x_{1}+x_{2},\;0.3y_{1}$) for LC (bottom). LC is characteristic of RSE. ***B***, ***C***, Time series of the periodic switch between DB and NS (***B***) and LC (***C***). Parameter settings correspond to region VII in Figure 34 and to region 12 in Figure 35. ***A***, Top, I.C = [0 −5 3 0 0 0.01], *T_s_* = [0 450], and *r * = * *0.01. For easier visualization, we plot the trajectory over *T_s_* = [96 244]. ***A***, Bottom, I.C = [9 −5 −1 0 0 0.01], *T_s_* = [0 200], and *r * = * *0.01. The coexistence of LC and *S* can be found in area II (Fig. 12*B*).

A (*z*, *x*_1_) bifurcation diagram is plotted in Figure 14*A* for *m* = −8 ($I_{ext_{2}}=0$). The trajectory characterizes the periodic switch between DB and NS. Parameter settings in Figure 14 correspond to area II in Figure 12*B*. Thus, LC and the periodic switch between DB and NS coexist, and are separated by a saddle periodic orbit (S). We plot LC, S, and the periodic switch between DB and NS in a (*Y*, *X*, *z*) phase space (Fig. 25*A*). We observe that DB occurs when the fast manifold of the SLE collapses to a point that traces out a line under the slow *z*-evolution. The ictal state thus reduces to a silent activity. LC and the periodic switch between DB and NS coexist in the phase space and the separatrix acts as a barrier between them. Time series are plotted in Figure 25*B* for the periodic switch between DB and NS, and in Figure 25*C* for LC. DB and NS are indicated by segment numbers 1 and 3, respectively.

##### Coexistence of LC and a NS

**Figure 26.**
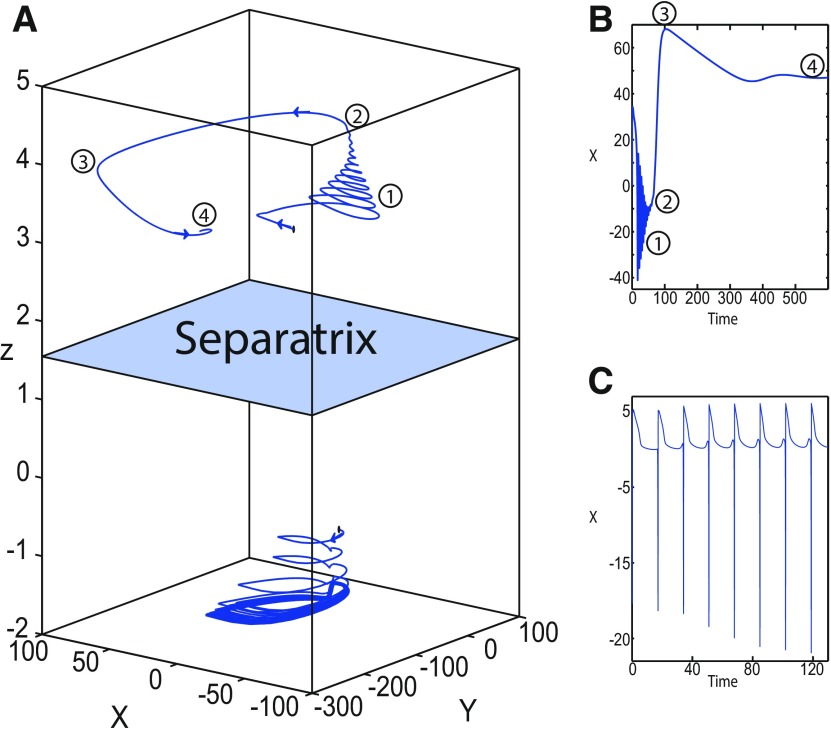
***A***, Coexistence of normal state and RSE. The simulations are performed without noise. The equilibrium point of NS and a stable LC coexist for *m * = * *0 and $x_{0}=-2.1$. Trajectory segments are numbered in ***A*** and ***B***. The transient seizure-like fast discharges correspond to the segment 1, and the NS to the segment 4. The equilibrium point of NS exists. After the transient seizure-like fast discharges, the Epileptor remains in NS. The arrows indicate the direction of trajectories. For easier visualization, we plot generalized coordinates (*X*, *Y*) corresponding to ($-35x_{1}+x_{2},\;15y_{1}$) for DB (top) and to ($-0.5x_{1}+x_{2},\;0.1y_{1}$) for LC (bottom). LC is characteristic of RSE. ***B***, ***C***, Time series of NS (***B***) and LC (***C***). Parameter settings correspond to region X in Figure 31 and to region 18 in Figure 32. The coexistence of LC and *S* can be found in area II (Fig. 12*A*).

Figure 17 shows that when decreasing *x*_0_, the *z*-nullcline is at the *Z*-lower branch, which consists of equilibrium points of the normal state (stable nodes). Let *m * = * *0, the equilibrium point is a stable node when *x*_0_ is below −2.1 (Fig. 3, area 5). LC and S coexist when *x*_0_ = −2.1 (Fig. 12*A*, area II). We plot LC, NS, and S in a (*Y*, *X*, *z*) phase space (Fig. 26*A*). LC and NS coexist in the phase space, and the separatrix acts as a barrier between them. Fast discharges and NS are indicated by segment numbers 1 and 4, respectively. After the transient seizure-like fast discharges, the Epileptor remains in the NS. Time series are plotted in Figure 26*B* for NS and in Figure 26*C* for LC.

##### Coexistence of LC and a DB

**Figure 27.**
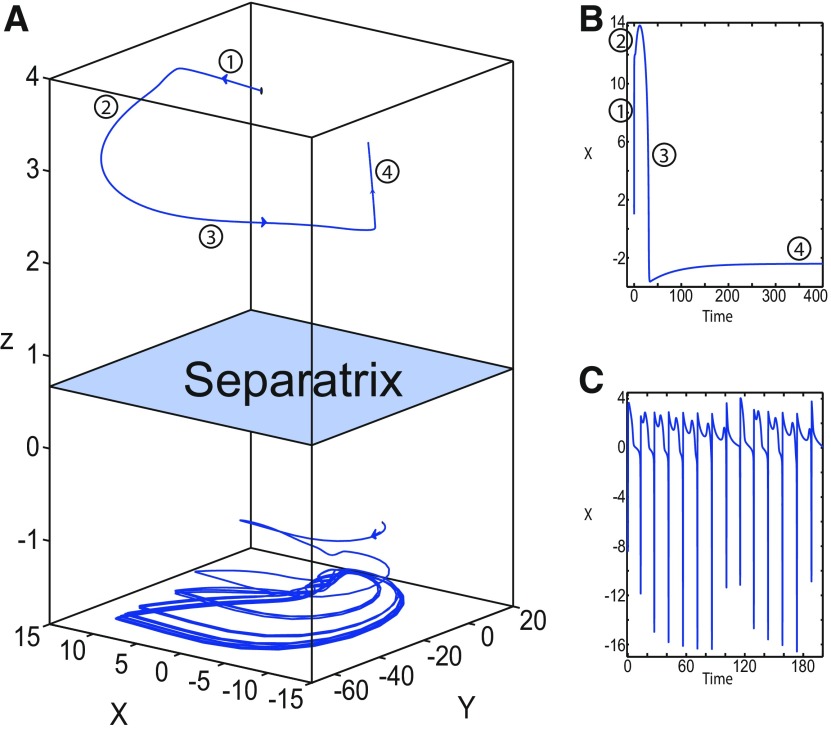
***A***, Coexistence of DB and RSE. The simulations are performed without noise. The equilibrium point of DB and a stable LC coexist for *m* = −8 and *x*_0_ = −0.6. Trajectory segments are numbered in ***A*** and ***B***. DB corresponds to the segment 4, and the NS to the segment 2. The equilibrium point of DB exists. The equilibrium point of NS does not. After a transient NS, the Epileptor stabilizes on DB. The arrows indicate the direction of trajectories. For easier visualization, we plot generalized coordinates (*X*, *Y*) corresponding to ($-10x_{1}+x_{2},\;5y_{1}$) for DB (top) and to ($-0.6x_{1}+x_{2},\;0.2y_{1}$) for LC (bottom). LC is characteristic of RSE. ***B***, ***C***, Time series of DB (***B***) and LC (***C***). Parameter settings correspond to region V in Figure 34 and to region 9 of 5 in Figure 35. ***A***, Top, I.C = [−0.1 −6 3.8 0 0 0.01], *T_s_* = [0 400], and *r * = * *0.01. ***A***, Bottom, I.C = [9 −5 −1 0 0 0.01], *T_s_* = [0 200], and *r * = * *0.009. The coexistence of LC and *S* can be found in area II (Fig. 12*B*).

Figure 16*D* shows that when increasing *x*_0_, the *z*-nullcline is at the *Z*-upper branch, which consists of equilibrium points of DB (stable nodes). Parameter settings in Figure 16*D* correspond to area II in Figure 12*B*, where LC and S coexist. We plot LC, DB, and S in a (*Y*, *X*, *z*) phase space (Fig. 27*A*). The transient NS and DB are indicated by segment numbers 2 and 4, respectively. After the transient normal state, the Epileptor model remains in the depolarization block. Time series are plotted in Figure 27*B* for DB and in Figure 27*C* for LC.

##### Coexistence of LC and a nonoscillatory state

**Figure 28.**
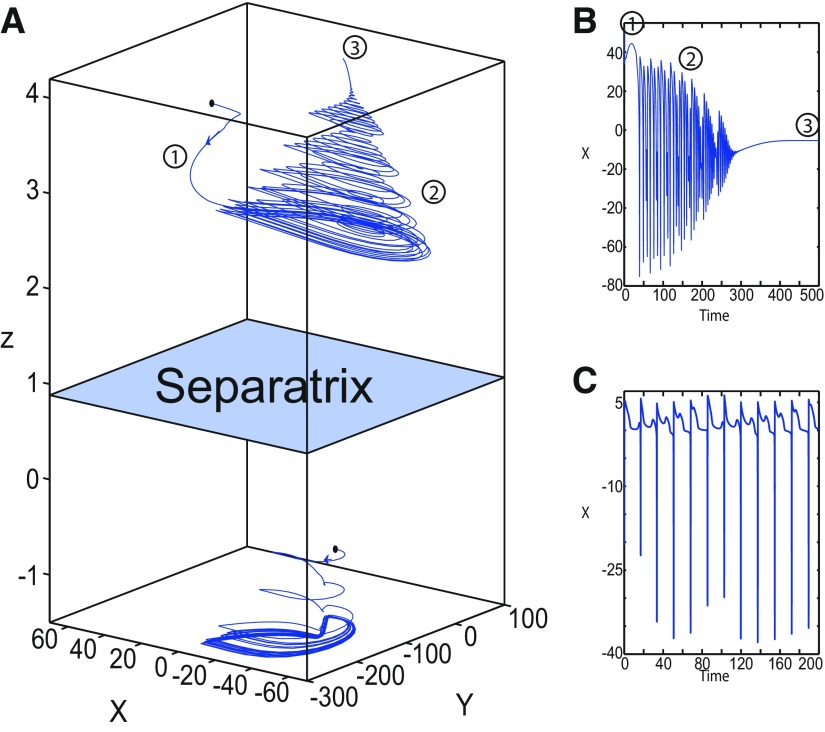
***A***, Coexistence of nonoscillatory state and RSE. Here, transient seizure-like fast discharges disappears through a SNIC bifurcation, and then the nonoscillatory state occurs. The simulations are performed without noise. The equilibrium point of the nonoscillatory state and a stable LC coexist for *m * = * *0 and *x*_0_ = −0.9 ($I_{ext_{2}}=0.45$). Trajectory segments are numbered in ***A*** and ***B***. The transient seizure-like fast discharges correspond to the segment 2, the transient NS to the segment 1, and the nonoscillatory state to the segment 4. The equilibrium point of nonoscillatory state exists. After the transients NS and then seizure-like fast discharges, the Epileptor remains in the nonoscillatory state. The arrows indicate the direction of trajectories. For easier visualization, we plot generalized coordinates (*X*, *Y*) corresponding to ($-35x_{1}+x_{2},\;15y_{1}$) for the nonoscillatory state (top) and to ($-0.5x_{1}+x_{2},\;0.1y_{1}$) for LC (bottom). LC is characteristic of RSE. ***B***, ***C***, Time series of the nonoscillatory state (***B***) and LC (***C***). Parameter settings correspond to region V in Figure 31 and to region 6 in Figure 32. ***A***, Top, I.C = [−1.5 −2.5 3.5 0 0 0.01], *T_s_* = [0 500], and *r * = * *0.007. ***A***, Bottom, I.C = [10 −5 −1 0 0 0.01], *T_s_* = [0 500], and *r * = * *0.004. The coexistence of LC and *S* can be found in area II (Fig. 12*A*).

**Figure 29.**
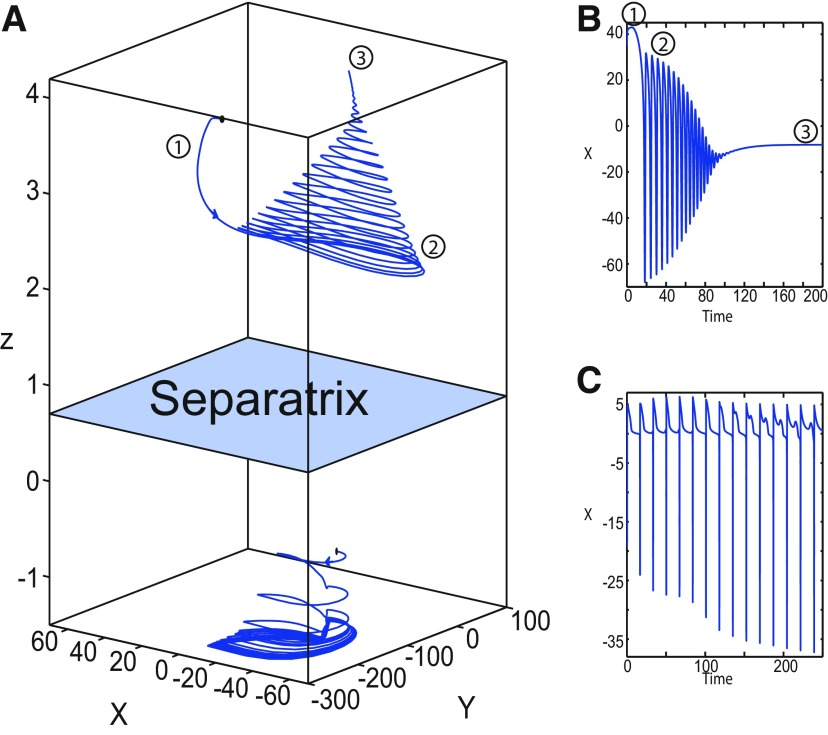
***A***, Coexistence of nonoscillatory state and RSE. Here a transient seizure-like fast discharges disappear through a Hopf bifurcation, and then the nonoscillatory state occurs. The simulations are performed without noise. The equilibrium point of the nonoscillatory state and a stable LC coexist for *m* = −0.5 and *x*_0_ = −0.8 ($I_{ext_{2}}=0$). Trajectory segments are numbered in ***A*** and ***B***. The transient seizure-like fast discharges correspond to the segment 2, the transient NS to the segment 1, and the nonoscillatory state to the segment 4. The equilibrium point of the nonoscillatory state exists. After the transients NS and then seizure-like fast discharges, the Epileptor remains in the nonoscillatory state. The arrows indicate the direction of trajectories. For easier visualization, we plot generalized coordinates (*X, Y*) corresponding to ($-35x_{1}+x_{2},\;15y_{1}$) for the nonoscillatory state (top) and to ($-0.5x_{1}+x_{2},\;0.1y_{1}$) for LC (bottom). LC is characteristic of RSE. ***B***, ***C***, Time series of the nonoscillatory state (***B***) and LC (***C***). Parameter settings correspond to region V in Figure 34 and to region 6 in Figure 32. ***A***, Top, I.C = [−1 −5.5 3.8 0 0 0.01], *T_s_* = [0 200], and *r * = * *0.02. ***A***, Bottom, I.C = [10 −5 −1 0 0 0.01], *T_s_* = [0 250], and *r * = * *0.004. The coexistence of LC and *S* can be found in area II (Fig. 12*B*).

**Figure 30.**
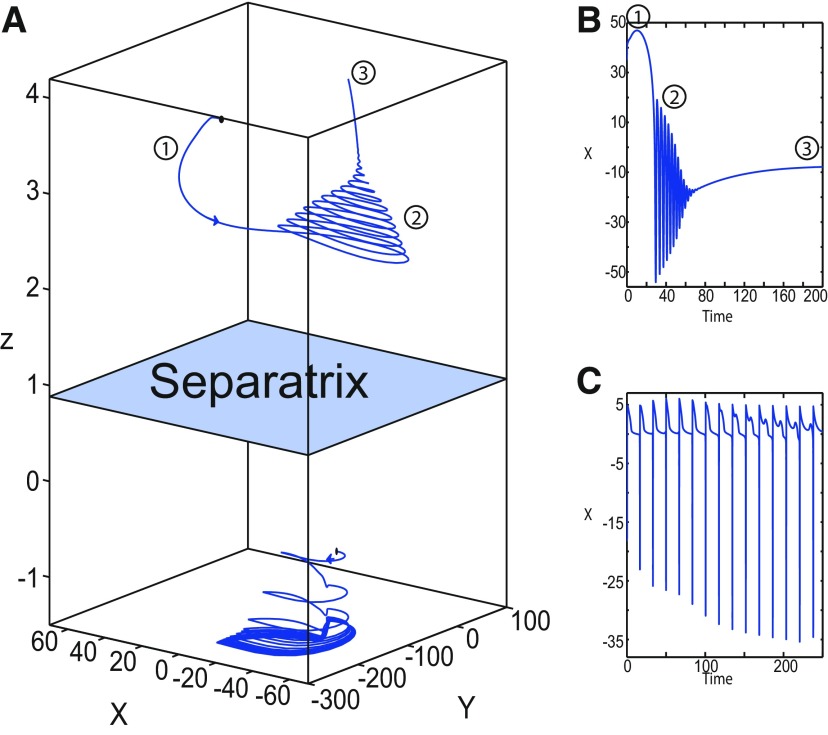
***A***, Coexistence of nonoscillatory state and RSE. Here after a transient NS, the Epileptor enters into the nonoscillatory state. The simulations are performed without noise. The equilibrium point of the nonoscillatory state and a stable LC coexist for *m* = −1 and *x*_0_ = −0.8 ($I_{ext_{2}}=0$). Trajectory segments are numbered in ***A*** and ***B***. The transient NS is indicated by (1) and the nonoscillatory state (final state) by (3). The equilibrium point of nonoscillatory state exists, which is a stable focus. After a transient NS, the Epileptor spirals into the equilibrium point (stable focus) and remains in the nonoscillatory state. The arrows indicate the direction of trajectories. For easier visualization, we plot generalized coordinates (*X*, *Y*) corresponding to ($-35x_{1}+x_{2},\;15y_{1}$) for the nonoscillatory state (top) and to ($-0.5x_{1}+x_{2},\;0.1y_{1}$) for LC (bottom). LC is characteristic of RSE. ***B***, ***C***, Time series of the nonoscillatory state (***B***) and LC (***C***). Parameter settings correspond to region V in Figure 34 and to region 6 in Figure 32. ***A***, Top, I.C = [−1 −5.5 3.5 0 0 0.01], *T_s_* = [0 200], and *r * = * *0.01. ***A***, Bottom, I.C = [10 −5 −1 0 0 0.01], *T_s_* = [0 250], and *r * = * *0.004. The coexistence of LC and *S* can be found in area II (Fig. 12*B*).

Figure 16*A–C* shows that when increasing *x*_0_, the equilibrium point C is at the *Z*-upper branch when $I_{ext_{2}}=0$ and at the upper branch above the *Z*-curve when $I_{ext_{2}}=0.45$. *C* (stable focus) is the equilibrium point of the nonoscillatory state. Figure 16, *A* and *B*, shows that after the transient normal state, and the transient seizure-like fast discharges, the Epileptor remains in the nonoscillatory state. Figure 16*C* shows that after the transient normal state, the Epileptor spirals into the equilibrium point of the nonoscillatory state exhibiting damped oscillations and then stabilizes at C. Parameter settings in Figure 16*A* correspond to area II in Figure 12*A*, and parameter settings in Figure 16, *B* and *C*, correspond to area II in Figure 12*B*, and then LC and S coexist. Only the nonoscillatory state is plotted in Figure 16*A–C*. We plot LC, the nonoscillatory state, and S in a (*Y*, *X*, *z*) phase space (Figs. 28 *A*, 29 *A*, 30 *A*). LC (below) and the nonoscillatory state (above) coexist. The nonoscillatory state shown in Figures 28 *A*, 29 *A*, and 30 *A* is the same as that shown in Figure 16*A–C*, respectively. NS and the nonoscillatory state are indicated by segment numbers 1 and 3, respectively. Time series for each case are plotted in Figures 28*B*, 29*B*, and 30*B* for the nonoscillatory states, and in Figures 28*C*, 29*C*, and 30*C* for LC.

#### Parameter space of equilibrium points and periodic orbits

We identified seven types of coexisting attractors: a coexistence of SLE and LC (Figs. 18–21), a coexistence of LC and periodic switch between nonoscillatory state and NS (Fig. 22), a coexistence of SLC and LC (Figs. 23, 24), a coexistence of LC and periodic switch between DB and NS (Fig. 25), a coexistence of NS and LC (Fig. 26), a coexistence of DB and LC (Fig. 27), and a coexistence of nonoscillatory state and LC (Figs. 28–30). SLEs are characterized by transitions between ictal and normal states. Depending on *m*, *x*_0_ and $I_{ext_{2}}$, these transitions occur through a fold/homoclinic bifurcation (Figs. 18, 19), a fold/circle bifurcation (Fig. 20), or a fold/Hopf bifurcation (Fig. 21).

**Figure 31.**
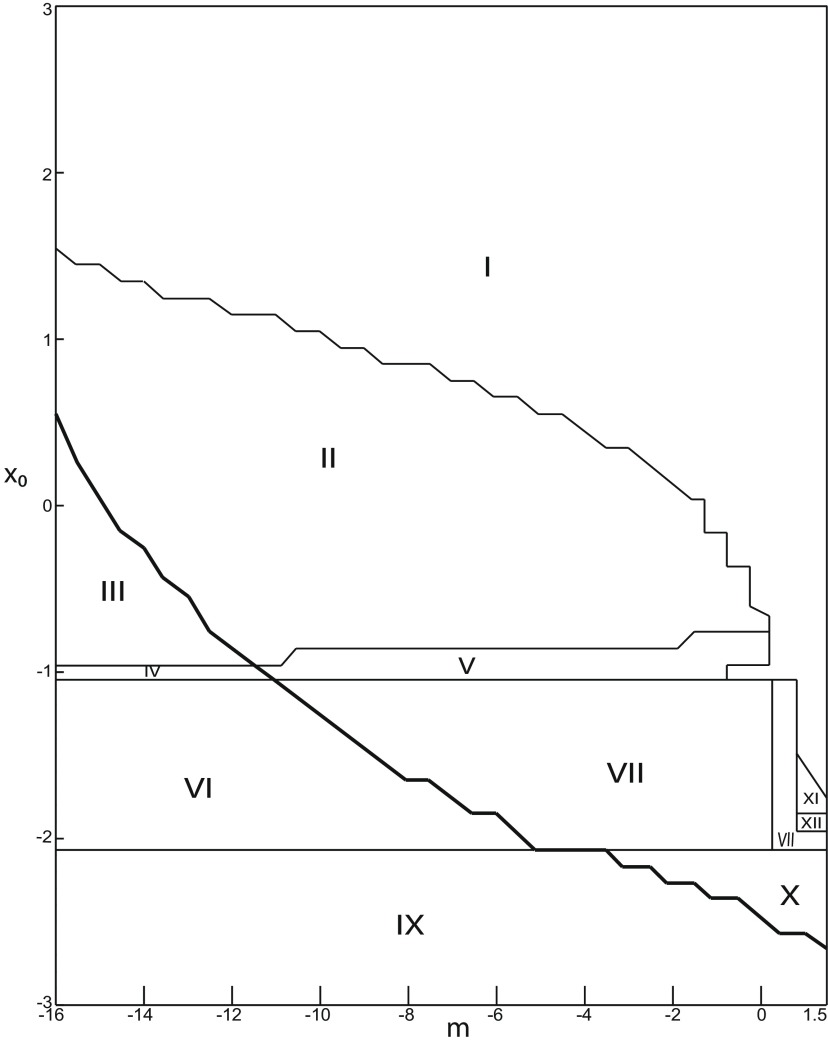
Parameter space of the Epileptor model with respect to the parameters *m* and *x*_0_ ($I_{ext2}=0.45$). There are 12 regions separated by a SNPO bifurcation (bold line). LC exists above and it does not exist below. LC exists in area I. Area II shows bistability of LC and SLC. In area III, only SLC exists. Only a nonoscillatory state with damped oscillation exists in area IV, and coexists with LC in area V. Only SLE exists in area VI, and coexists with LC in areas VII and VIII. SLE occurs through a fold/circle bifurcation in areas VI and VII, and through a fold/homoclinic bifurcation in area VIII. NS exists in area IX and coexists with LC in area X. In areas XI and XII, LC coexists with a chaotic state which is periodic in area XI, and it does not in area XII.

**Figure 32.**
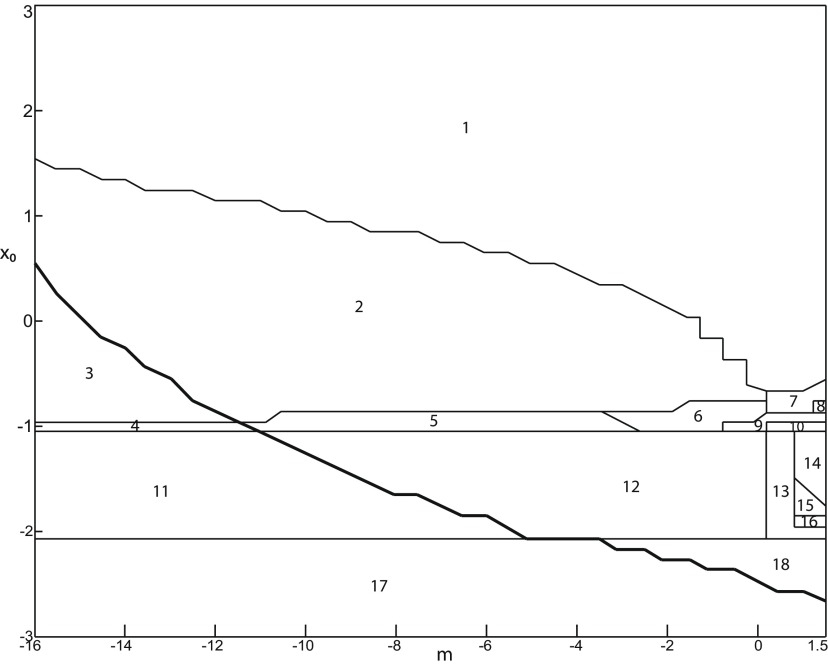
The Epileptor parameter space of equilibrium points and periodic orbits with respect to the parameters *m* and *x*_0_ ($I_{ext2}=0.45$). There are 18 areas separated by a SNPO bifurcation (bold line). LC exists above and it does not exist below. The *z*-nullcline intersects the (*z*, *x*_1_) curve at different equilibrium points. The equilibrium point is an unstable focus for areas 1 through 3. The equilibrium points are a stable node, an unstable focus and a saddle in areas 4 and 5. The equilibrium points are a stable focus, an unstable focus, and a saddle in area 6. The equilibrium points are one saddle and two unstable foci in area 7. The equilibrium points are a saddle, an unstable focus, and an unstable node in area 8. The equilibrium points are one unstable focus and two saddles in area 9. The equilibrium points are three saddles in area 10. The equilibrium point is a saddle for areas 11 through 16. The equilibrium point is a stable node in areas 17 and 18. In areas 1, 7, 8, 9, 10, and 14, only LC exists. Area 2 presents bistability of LC and SLC. In area 3, only SLC exists. In area 4, only a nonoscillatory state exists. In areas 5 and 6, LC and a nonoscillatory state coexist. Only SLE exists in area 11, and coexists with LC in areas 12 and 13. SLE occurs through a fold/circle bifurcation in areas 11 and 12, and through a fold/homoclinic bifurcation in area 13. NS exists in area 17 and coexists with LC in area 18. In areas 15 and 16, LC coexists with a chaotic state which is periodic in area 15, and it does not in area 16.

To characterize the different types of the coexisting attractors, we explore a (*m*, *x*_0_) parameter space of periodic orbits and equilibrium points in Figures 31 and 32 for $I_{ext_{2}}=0.45$.

##### Parameter space of the Epileptor model for $I_{ext_{2}}=0.45$

The parameter space is divided into two parts which are separated by a bold line (SNPO bifurcation; Fig. 31). Above the bold line, LC exists but does not exist below it. Twelve areas exist. LC exists in area I and coexists with SLC in area II. Only SLC exists in area III. The nonoscillatory state exists in area IV and coexists with LC in area V. Only SLE exists in area VI and coexists with LC in areas VII and VIII. An SLE occurs through a fold/circle bifurcation in areas VI and VII. An SLE occurs through a fold/homoclinic bifurcation in area VIII. NS exists in area IX and coexists with LC in area X. LC coexists with a chaotic state in areas XI and XII.

For each area, the Epileptor model has different equilibrium points, depending on *m* and *x*_0_. To explore this further, we determine the equilibrium points and the periodic orbits of each area (I-XII) by using 32, which shows 18 (1–18) areas:

*(I) LC:* Area I in Figure 31 is composed of areas 1, 7, 8, 9, 10, and 14 in Figure 32. The *z*-nullcline intersects the $<x_{1}>$-curve at one periodic orbit, which corresponds to LC. The *z*-nullcline intersects the (*z*, *x*_1_) curve at different equilibrium points. The equilibrium point is an unstable focus in area 1 and a saddle in area 14. The equilibrium points are one saddle and two unstable foci in area 7. The equilibrium points are one unstable focus and two saddles in area 9. The equilibrium points are three saddles in area 10. The equilibrium points are a saddle, an unstable focus, and an unstable node in area 8. Trajectories exhibit only a fast-slow cyclic behavior (LC), which is plotted in Figures 9*D* and 10.

*(II) LC and SLC:* Area II in Figure 31 corresponds to area 2 in Figure 32. The *z*-nullcline intersects the $<x_{1}>$-curve at three periodic orbits in area 2, which correspond to LC, S, and SLC, respectively. The *z*-nullcline intersects the (*z*, *x*_1_) curve at one equilibrium point, which is an unstable focus. LC and SLC coexist. The stable manifold of the saddle periodic orbit (S) separates the basin of attraction of LC and the basin of attraction of SLC. Trajectories exhibit two periodic solutions depending on initial conditions. The first solution corresponds to a fast-slow cyclic behavior (LC). The second solution corresponds to a periodic solution with a small amplitude (SLC).

*(III) SLC:* Area III in Figure 31 corresponds to area 3 in Figure 32. The *z*-nullcline intersects the $<x_{1}>$-curve at one periodic orbit in area 3, which corresponds to SLC. Area three is below the SNPO bifurcation (bold line), which means that SLC exists after a SNPO bifurcation. The *z*-nullcline intersects the (*z*, *x*_1_) curve at one equilibrium point, which is an unstable focus. The trajectories exhibit only a periodic solution with a small amplitude (SLC).

*(IV) Nonoscillatory state:* Area IV in Figure 31 corresponds to area 4 in Figure 32. Here, the *z*-nullcline and the $<x_{1}>$-curve do not intersect, hence periodic orbits LC, S, and SLC do not exist. The *z*-nullcline intersects the (*z*, *x*_1_) curve at different equilibrium points. The equilibrium points are a stable node, an unstable focus, and a saddle in area 4. Trajectories remain in the nonoscillatory state.

*(V) LC and a nonoscillatory state:* Area V in Figure 31 is composed of areas 5 and 6 in Figure 32. The *z*-nullcline intersects the $<x_{1}>$-curve at two periodic orbits in areas 5 and 6, which correspond to LC and S. The *z*-nullcline intersects the (*z*, *x*_1_) curve at different equilibrium points. The equilibrium points are a stable node, an unstable focus, and a saddle in area 5. The equilibrium points are a stable focus, an unstable focus, and a saddle in area 6. Trajectories either exhibit a fast-slow cyclic behavior (LC) or remain in the nonoscillatory state. The coexistence of LC and the nonoscillatory state is plotted in Figure 28*A*. The equilibrium points belong to area 6 in Figure 32. Time series are plotted in Figures 28*B* for the nonoscillatory state and in Figure 28*C* for LC.

*(VI) SLE with a fold/circle bifurcation:* Area VI in Figure 31 corresponds to area 11 in Figure 32. Here, the *z*-nullcline and the $<x_{1}>$-curve do not intersect, hence periodic orbits LC, S, and SLC do not exist. The *z*-nullcline intersects the *Z*-middle branch, which consists of saddles. Thus, a unique saddle equilibrium point exists. Since *m *≤* * 0, an SLE occurs through a fold/circle bifurcation.

*(VII) LC and SLE with a fold/circle bifurcation:* Area VII in Figure 31 corresponds to area 12 in Figure 32. Here, the equilibrium point is a saddle, and an SLE occurs through a fold/circle bifurcation. The *z*-nullcline intersects the $<x_{1}>$-curve at two periodic orbits, which correspond to LC and S. Then LC and an SLE with a fold/circle bifurcation coexist and are separated by a saddle periodic orbit (S). This coexistence is plotted in Figure 20A. Time series are plotted in Figure 20*B* for SLE and in Figure 20*C* for LC.

*(VIII) LC and SLE with a fold/homoclinic bifurcation:* Area VIII in Figure 31 corresponds to area 13 in Figure 32. Here, the equilibrium point is a saddle, and an SLE occurs through a fold/homoclinic bifurcation. The *z*-nullcline intersects the $<x_{1}>$-curve at two periodic orbits, which correspond to LC and S. Then LC and SLE with a fold/homoclinic bifurcation coexist and are separated by a saddle periodic orbit (S). This coexistence is plotted in Figure 18*A*. Time series are plotted in Figure 18*B* for SLE and in Figure 18*C* for LC.

*(IX) NS:* Area IX in Figure 31 corresponds to area 17 in Figure 32. The *z*-nullcline and the $<x_{1}>$-curve do not intersect, hence periodic orbits LC, S, and SLC do not exist. The *z*-nullcline intersects the (*z*, *x*_1_) curve at the *Z*-lower branch, and then the equilibrium point is a stable node in area 17. The Epileptor remains in NS after a transient period, which is shown in Figure 17

*(X) LC and NS:* Area X in Figure 31 corresponds to area 18 in Figure 32. The *z*-nullcline intersects the $<x_{1}>$-curve at two periodic orbits, which correspond to LC and S. The *z*-nullcline intersects the (*z*, *x*_1_) curve at the *Z*-lower branch, which is a stable node in area 18. Then LC and NS coexist and are separated by a saddle periodic orbit (S). This coexistence is plotted in Figure 26*A*. Time series are plotted in Figure 26*B* for NS and in Figure 26*C* for LC.

*(XI*, *XII) LC and a chaotic state:* Areas XI and XII in Figure 31 correspond to areas 15 and 16 in Figure 32. The *z*-nullcline intersects the $<x_{1}>$-curve at three periodic orbits in area 15. Then LC, S, and SLC coexist. S acts as a separatrix between the basin of attraction of LC and the basin of attraction of SLC. Then trajectories exhibit either LC or SLC in area 15. The *z*-nullcline intersects the $<x_{1}>$-curve at two periodic orbits in area 16, which correspond to LC and S. The *z*-nullcline intersects the *Z*-middle branch which consists of saddles. An SLE occurs through a fold/homoclinic bifurcation in area 16. Then LC and SLE coexist in area 16. S acts as a separatrix between the basin of attraction of LC and the basin of attraction of SLE in area 16.

**Figure 33.**
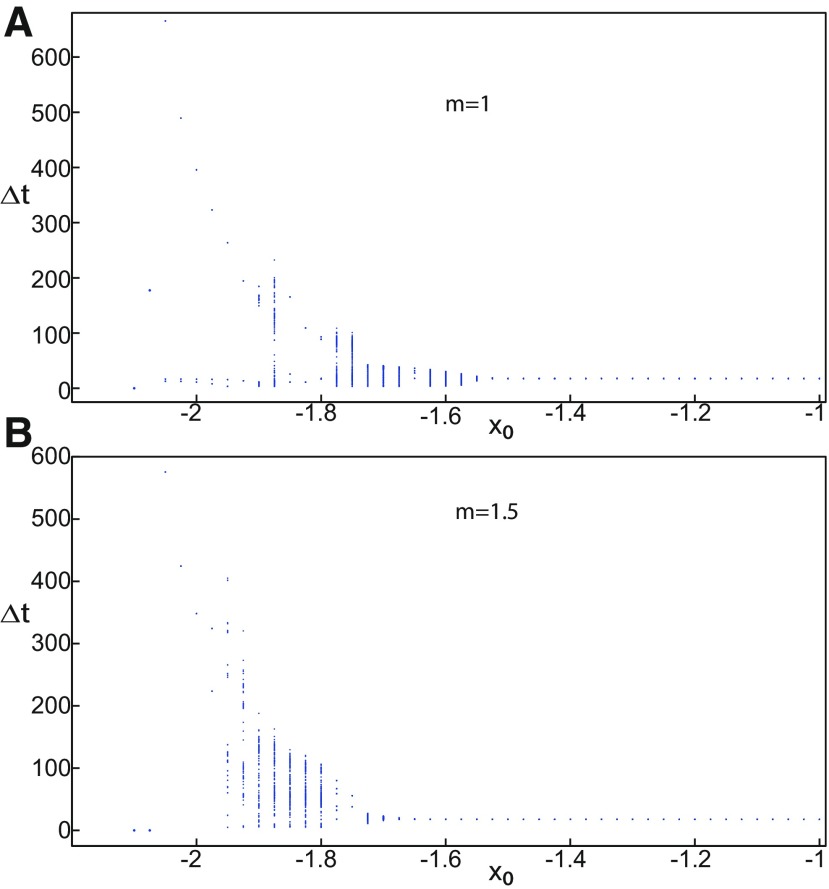
Interspike intervals Δt ($I_{ext_{2}}=0.45$). ***A***, ***B***, *m * = * *1 (***A***) and *m * = * *1.5 (***B***).

We calculate the times between two successive spikes, which are referred to as the interspike intervals (ISIs) with respect to *x*_0_ in Figure 33*A* for *m * = * *1 and in Figure 33*B* for *m * = * *1.5. The system is chaotic when the ISI is irregular. Therefore, LC and the chaotic state coexist in areas 15 and 16 and are separated by a saddle periodic orbit (S).

**Figure 34.**
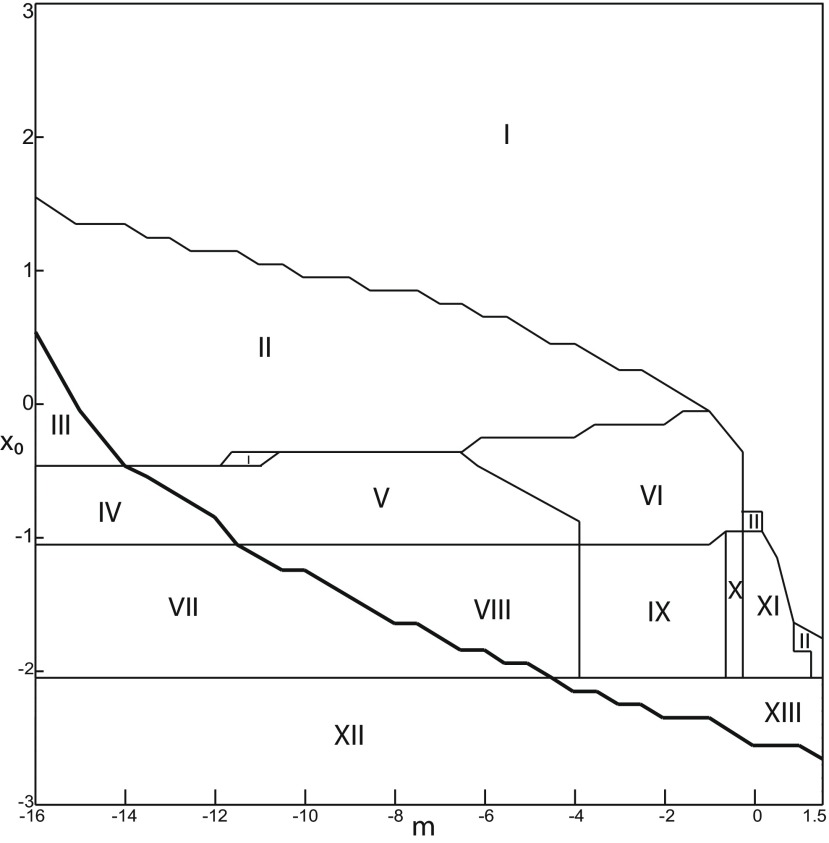
Parameter space of the Epileptor model with respect to the parameters *m* and *x*_0_ ($I_{ext2}=0$). There are 13 regions separated by a SNPO bifurcation (bold line). LC exists above, and it does not exist below. LC exists in area I. Area II presents a bistability of LC and SLC. Only SLC exists in area III. DB exists for areas IV, V, VII, and VIII. In area IV, only DB exists. In area V, DB and LC coexist. In area VII, only a periodic switch between DB and NS exists. In area VIII, a periodic switch between DB and NS coexists with LC. Increasing *m*, DB locks into nonoscillatory state with damped oscillation coexisting with LC in area VI. A periodic switch between a nonoscillatory state and a NS coexists with LC in area IX. SLE coexists with LC in areas X and XI. SLE occurs through a fold/Hopf bifurcation in area X and through a fold/homoclinic bifurcation in area XI. NS exists in area XII and coexists with LC in area XIII.

**Figure 35.**
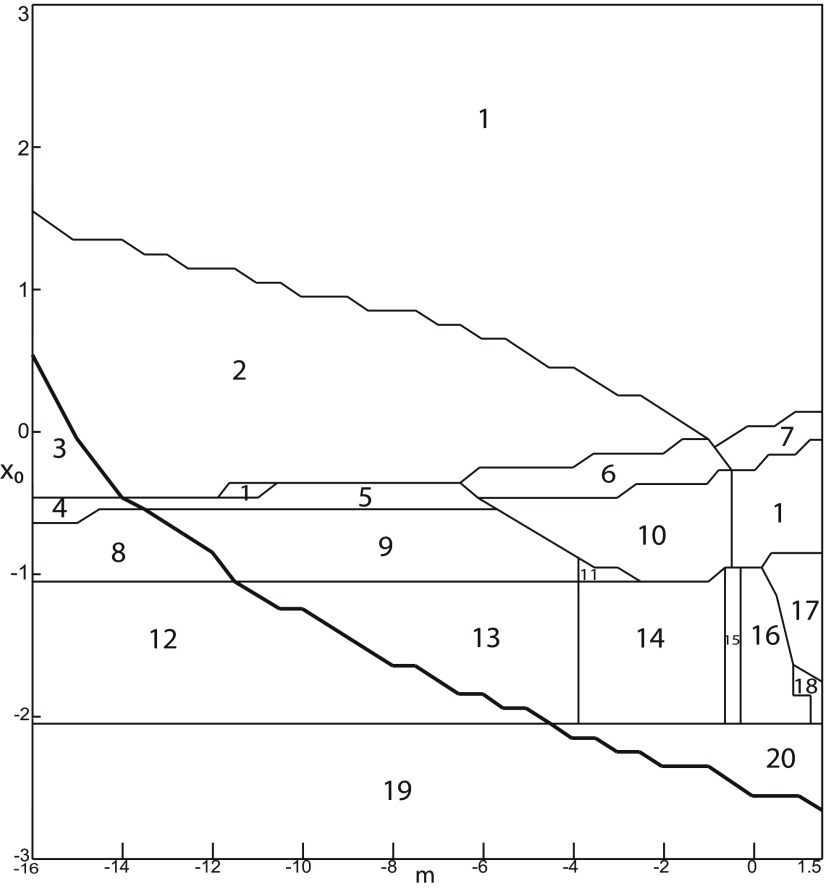
The Epileptor parameter space of equilibrium points and periodic orbits with respect to the parameters *m* and *x*_0_ ($I_{ext2}=0$). There are 20 regions separated by a SNPO bifurcation (bold line). LC exists above and it does not exist below. The *z*-nullcline intersects the (*z*, *x*_1_) curve at different equilibrium points. The equilibrium point is an unstable focus for areas 1 through 3. The equilibrium points are a stable node, an unstable focus, and a saddle in areas 4 and 5. The equilibrium points are a stable focus, an unstable focus, and a saddle in area 6. The equilibrium points are one saddle and two unstable foci in area 7. The equilibrium point is a stable node in areas 8, 9, 11, 19, and 20. The equilibrium point is a stable focus in area 10. The equilibrium point is a saddle for areas 12 through 18. In areas 1, 7, and 17, only LC exists. Areas 2 and 18 present bistability of LC and SLC. In area 3, only SLC exists. In areas 4 and 8, only DB exists. In areas 5 and 9, LC and DB coexist. In areas 6, 10, and 11, LC and nonoscillatory state coexist. Only a periodic switch between DB and NS exists in area 12, and coexists with LC in area 13. A periodic switch between a nonoscillatory state and NS coexists with LC in area 14. LC and SLE coexist in areas 15 and 16. SLE occurs through a fold/Hopf bifurcation in area 15 and through a fold/homoclinic bifurcation in area 16. NS exists in area 19 and coexists with LC in area 20.

The parameter space of the Epileptor model includes attractors as LC, SLE, and normal state, but neither DB nor SLE with a fold/Hopf bifurcation. In fact, using bifurcation analysis, we demonstrated that DB and Hopf bifurcation appeared when decreasing $I_{ext_{2}}$. To characterize these attractors, we explore a (*m*, *x*_0_) parameter space of periodic orbits and equilibrium points in Figures 34 and 35 for $I_{ext_{2}}=0$.

##### Parameter space of the Epileptor model for $I_{ext_{2}}=0$

The parameter space is divided into two parts, which are separated by a bold line (SNPO bifurcation) (Fig. 34). Above the line, LC exists and does not exist below it. Thirteen areas exist. LC exists in area I and coexists with SLC in area II. Only SLC exists in area III. A DB exists in area IV and coexists with LC in area V. A periodic switch between a DB and a NS exists in area VII and coexists with LC in area VIII. A nonoscillatory state coexists with LC in area VI, and a periodic switch between a nonoscillatory state and a NS coexists with LC in area IX. LC and SLE coexist in areas X and XI. an SLE occurs through a fold/Hopf bifurcation in area X and through a fold/homoclinic bifurcation in area XI. A normal state exists in area XII and coexists with LC in area XIII.

For each area, the Epileptor model has different equilibrium points depending on *m* and *x*_0_. To analyze this further, we determine the equilibrium points and the periodic orbits of each area (I-XIII) by using 35, which shows 20 (1–20) areas:

*(I) LC:* Area (I) in Figure 34 is composed of areas 1, 7, and 17 in Figure 35. The *z*-nullcline intersects the $<x_{1}>$-curve at one periodic orbit, which is LC. The *z*-nullcline intersects the (*z*, *x*_1_) curve at different equilibrium points. The equilibrium point is an unstable focus in area 1 and a saddle in area 17. The equilibrium points are one saddle and two unstable foci in area 7. Trajectories exhibit only a fast-slow cyclic behavior (LC).

*(II) LC and SLC:* Area (II) in Figure 34 is composed of areas 2 and 18 in Figure 35. The *z*-nullcline intersects the $<x_{1}>$-curve at three periodic orbits in areas 2 and 18, which correspond to LC, S, and SLC, respectively. Then LC and SLC coexist in areas 2 and 18. The stable manifold of a saddle periodic orbit (S) separates the basin of attraction of LC and the basin of attraction of SLC. The *z*-nullcline intersects the (*z*, *x*_1_) curve at one equilibrium point, which is an unstable focus in area 2 and a saddle in area 18. The trajectories exhibit two periodic solutions depending on the initial conditions. The first solution corresponds to a fast-slow cyclic behavior (LC). The second solution corresponds to a periodic solution with a small amplitude (SLC). Moreover, the SLC behavior depends on the stability of the equilibrium point. The SLC behavior is plotted in Figure 13*A* for area 18 and in Figure 13*B* for area 2. The coexistence of LC and SLC is plotted in a phase space, in Figure 23*A* for area 18, and in Figure 24*A* for area 2. Time series are plotted in Figure 23*B* for SLC and in Figure 23*C* for LC, for area 18. Time series are plotted in Figure 24*B* for SLC and in Figure 24*C* for LC, for area 2.

*(III) SLC:* Area III in Figure 34 corresponds to area 3 in Figure 35. The *z*-nullcline intersects the $<x_{1}>$-curve at one periodic orbit in area 3, which corresponds to SLC. Area 3 is below the SNPO bifurcation (bold line), which means that SLC exists after an SNPO bifurcation occurs. The *z*-nullcline intersects the (*z*, *x*_1_) curve at one equilibrium point, which is an unstable focus. Trajectories exhibit only a periodic solution with a small amplitude (SLC). The SLC behavior is plotted in Figures 13*B* and 24*B*.

*(IV) DB:* Area IV in Figure 34 is composed of areas 4 and 8 in Figure 35. Here, the *z*-nullcline and the $<x_{1}>$-curve do not intersect, hence periodic orbits LC, S, and SLC do not exist. The *z*-nullcline intersects the (*z*, *x*_1_) curve at different equilibrium points. The equilibrium point is a stable node in area 8. The equilibrium points are a stable node, an unstable focus, and a saddle in area 4. The Epileptor remains in DB after a transient period, which is plotted in Figures 16*D* and 27*B*.

*(V) LC and a DB:* Area V in Figure 34 is composed of areas 5 and 9 in Figure 35. The *z*-nullcline intersects the $<x_{1}>$-curve at two periodic orbits, which correspond to LC and S. The *z*-nullcline intersects the (*z*, *x*_1_) curve at different equilibrium points. The equilibrium points are a stable node, an unstable focus, and a saddle in area 5. The equilibrium point is a stable node in area 9. Trajectories either exhibit a fast-slow cyclic behavior (LC) or enter into a DB. The coexistence of LC and DB is plotted in Figure 27*A*. Time series are plotted in Figure 27*B* for DB and in Figure 27*C* for LC.

*(VI) LC and a nonoscillatory state:* Area VI in Figure 34 is composed of areas 6, 10, and 11 in Figure 35. The *z*-nullcline intersects the $<x_{1}>$-curve at two periodic orbits, which correspond to LC and S. The *z*-nullcline intersects the (*z*, *x*_1_) curve at different equilibrium points. The equilibrium points are a stable focus, an unstable focus, and a saddle in area 6. The equilibrium point is a stable focus in area 10 and a stable node in area 11. Trajectories either exhibit a fast-slow cyclic behavior (LC) or remain in a nonoscillatory state. The coexistence of LC and the nonoscillatory state is plotted in Figures 29*A* and 30*A*, which corresponds to area 10 in Figure 35. Time series are plotted in Figures 29*B* and 30*B* for the nonoscillatory state, and in Figures 29*C* and 30*C* for LC.

*(VII) Periodic switch between DB and NS:* Area VII in Figure 34 corresponds to area 12 in Figure 35. Here, the *z*-nullcline and the $<x_{1}>$-curve do not intersect, hence LC, S, and SLC do not exist. The *z*-nullcline intersects the *Z*-middle branch which consists of saddles. Then only a saddle equilibrium point exists in area 12. The ictal state of SLE reduces to DB with decreasing *m*. Periodic switch between DB and NS occurs through a fold/fold bifurcation, and is plotted in Figures 14 and 25*B*.

*(VIII) LC and a periodic switch between DB and NS:* Area VIII in Figure 34 corresponds to area 13 in Figure 35. The *z*-nullcline intersects the $<x_{1}>$ curve at two periodic orbits, which correspond to LC and S. The equilibrium point is a saddle, and the ictal state of an SLE is reduced to DB, with decreasing *m*. Periodic switch between DB and NS occurs through a fold/fold bifurcation, and coexists with LC. Both are separated by a saddle periodic orbit (S). The coexistence of LC and periodic switch between DB and NS is plotted in Figure 25*A*. Time series are plotted in Figure 25*B* for periodic switch between DB and NS, and in Figure 25*C* for LC.

*(IX) LC and a periodic switch between nonoscillatory state and NS:* Area IX in Figure 34 corresponds to areas 14 in Figure 35. The *z*-nullcline intersects the $<x_{1}>$ curve at two periodic orbits, which correspond to LC and S. Here, there is a unique saddle equilibrium point. The SLE attractor reduces to a periodic switch between nonoscillatory state and normal state, which occurs through a fold/fold bifurcation in area 14, and coexists with LC. Both are separated by a saddle periodic orbit (S). This coexistence is plotted in Figure 22*A*. Time series are plotted in Figure 22*B* for the periodic switch between nonoscillatory state and NS, and in Figure 22*C* for LC.

*(X) LC and SLE with a fold/Hopf bifurcation:* Area X in Figure 34 corresponds to area 15 in Figure 35. The *z*-nullcline intersects the $<x_{1}>$-curve at two periodic orbits, which correspond to LC and S. There is a unique saddle equilibrium point, and an SLE occurs through a fold/Hopf bifurcation (Fig. 7). Then LC and SLE with a fold/Hopf bifurcation coexist and are separated by a saddle periodic orbit (S). This coexistence is plotted in Figure 22*A*. Time series are plotted in Figure 21*B* for SLE and in Figure 21*C* for LC.

*(XI) LC and SLE with a fold/homoclinic bifurcation:* Area XI in Figure 34 corresponds to area 16 in Figure 35. The *z*-nullcline intersects the $<x_{1}>$-curve at two periodic orbits, which correspond to LC and S. A unique saddle equilibrium point exists, and an SLE occurs through a fold/homoclinic bifurcation in area 16 (Fig. 6). Then LC and SLE coexist and are separated by a saddle periodic orbit (S). This coexistence is plotted in Figure 19*A*. Time series are plotted in Figure 19*B* for SLE and in Figure 19*C* for LC.

*(XII) A NS:* Area XII in Figure 34 corresponds to area 19 in Figure 35. Here, the *z*-nullcline and the $<x_{1}>$-curve do not intersect, hence periodic orbits LC, S, and SLC do not exist. The *z*-nullcline intersects the (*z*, *x*_1_) curve at the *Z*-lower branch, and then the equilibrium point is a stable node in area 19. The Epileptor remains in NS after a transient period, which is shown in Figure 17.

*(XIII) LC and a NS:* Area XIII in Figure 34 corresponds to area 20 in Figure 35. The *z*-nullcline intersects the $<x_{1}>$-curve at two periodic orbits, which correspond to LC and S. The *z*-nullcline intersects the (*z*, *x*_1_) curve at the *Z*-lower branch, and hence the equilibrium point is a stable node in area 20. Then LC and NS coexist and are separated by a saddle periodic orbit (S). This coexistence is plotted in Figure 26*A*. Time series are plotted in Figure 26*B* for NS and in Figure 26*C* for LC.

#### Epileptor behaviors as a function of *m* and *x*_0_

**Figure 36.**
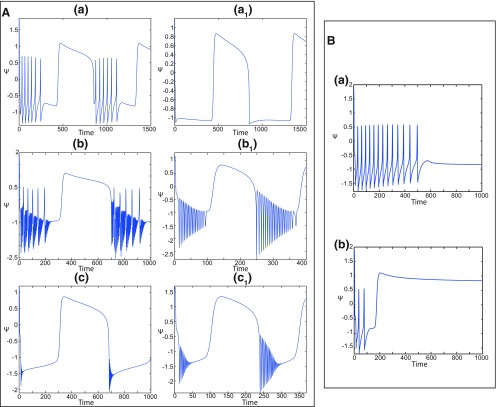
***A***, Deterministic time series of the Epileptor model *ψ* varying *m* and *x*_0_. ***a***, ***a_1_***, *m* = −12 and *x*_0_ = −1.6. *I*_ext2_ = 0.45 (***a***) and *I*_ext2_ = 0 (***a_1_***). The transitions between ictal and normal states occur through a fold/circle bifurcation (***a***), and they reduce to transitions between DB and NS (***a_1_***). ***b***, ***b_1_***, *m * = * *0 and *x*_0_ = −1.6. *I*_ext2_ = 0.45 (***b***) and *I*_ext2_ = 0 (***b_1_***). The transitions between ictal and normal states occur through a fold/circle bifurcation (***b***), and they reduce to transitions between nonoscillatory state and NS (***b_1_***). ***c***, ***c_1_***, *x*_0_ = −1.6 and *I*_ext2_ = 0. *m* = −1 (***c***) and *m* = −0.5 (***c_1_***). The transitions between nonoscillatory state and NS occur through a fold/fold bifurcation (***c***) and a fold/Hopf bifurcation (***c_1_***). *r * = * *0.0009 for (***a***, ***a_1_***), *r * = * *0.001 for (***b***, ***b_1_***), and *r * = * *0.0009 for (***c***, ***c_1_***). ***B***, Deterministic time series of the Epileptor model *ψ*, which show the Epileptor remaining in a nonoscillatory state, with *m* = −2, *x*_0_ = −1 and (***a***) in NS, with *m* = −2, *x*_0_ = −2.5 (***b***). *r * = * *0.001 for ***a*** and ***b***.

**Figure 37.**
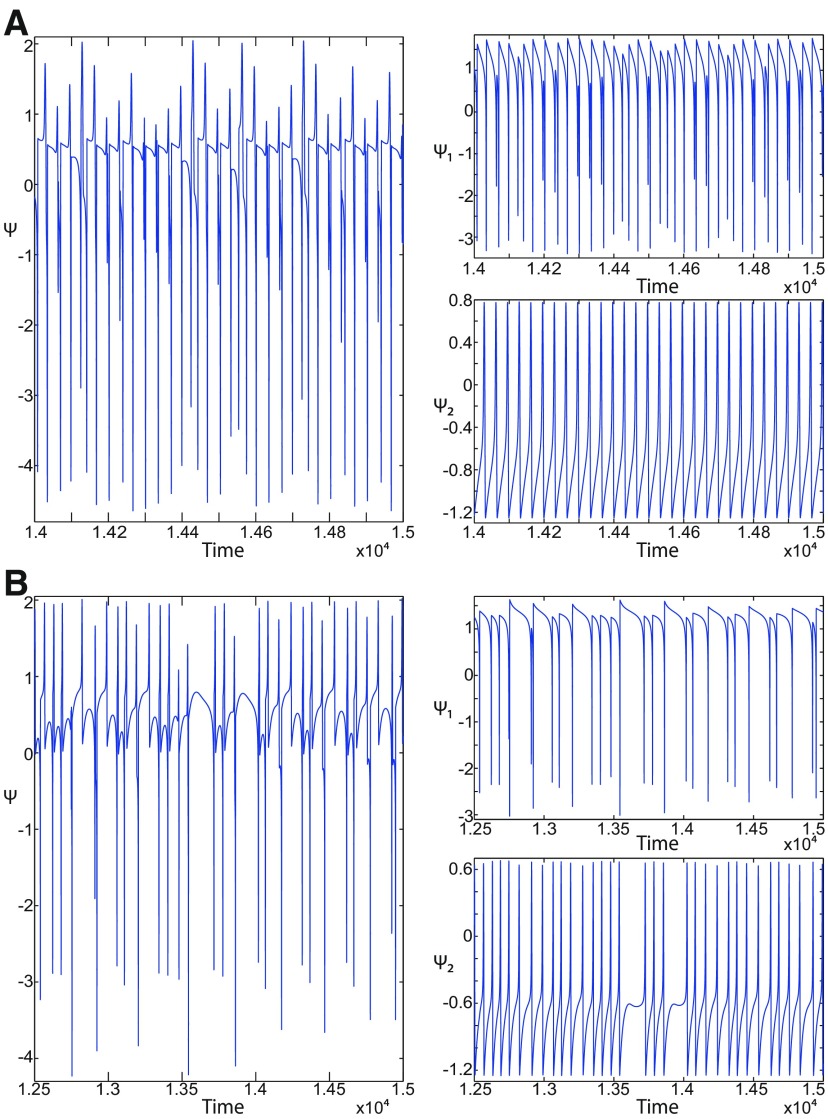
***A***, ***B***, Deterministic time series of the Epileptor model *ψ*, subsystem 1 *ψ*_1_ and subsystem 2 *ψ*_2_ with a chaotic spiking for *m * = * *1, *x*_0_ = −1.6 (***A***), and a chaotic transition between ictal and normal states for *m * = * *1.5, *x*_0_ = −1.9 (***B***).

Different areas exist in the parameter space of the Epileptor model as *m* and *x*_0_ vary (Figs. 31, 34). We show the behavior of the SLE attractor in Figure 36*A*, *a*, *b*, *b*_1_, and *c*_1_; the periodic switch between DB and NS in Figure 36 *A*, *a*_1_; the periodic switch between nonoscillatory state and NS in Figure 36 *A*, *c*; the nonoscillatory state in Figure 36*B*, *a*; the normal state in Figure 36*B*, *b*; *s* and the chaotic state in Figure 37.

*SLE*: it occurs through:

1. A fold/circle bifurcation in Figure 36*A*, *a* and *b*, and corresponds to areas VI and VII, respectively, in Figure 31.
2. A fold/homoclinic bifurcation in Figure 36*A*, *b*_1_, and corresponds to area XI in Figure 34.
3. A fold/Hopf bifurcation in Figure 36 *A*, *c*_1_, and corresponds to area X in Figure 34.

*Periodic switch between DB and NS*: The switch occurs through a fold/fold bifurcation in Figure 36*A*, *a*_1_, and corresponds to area VII in Figure 34, which shows a periodic switch between DB and NS.

*Periodic switch between a nonoscillatory state and NS:* The switch occurs through a fold/fold bifurcation in Figure 36*A*, *c*, and corresponds to area IX in Figure 34, which shows a periodic switch between nonoscillatory state and NS.

*Nonoscillatory state*: The Epileptor remains in the nonoscillatory state after a transient seizure-like fast discharges (Fig. 36*A*). Parameter settings correspond to area V in Figure 31 and to area 6 in Figure 32.

*Depolarization block*: The Epileptor remains in the DB after a transient NS (Fig. 27*A*). Parameter settings correspond to area V in Figure 34 and to area 5 in Figure 32.

*Normal state*: The Epileptor remains in the NS after a transient seizure-like fast discharges (Fig. 36*B*, *b*). Parameter settings correspond to area IX in Figure 31 and to area 17 in Figure 32.

*Chaotic state*: The chaotic state exists in areas XI and XII ($I_{ext_{2}}=0.45$; Fig. 31). Time series of the Epileptor model *ψ*, subsystem 1 *ψ*_1_, and subsystem 2 *ψ*_2_ are plotted in Figure 37. Figure 37*A* correspond to area XI in Figure 31, and Figure 37*B* correspond to area XII in Figure 31.

### Subsystem 1

Here, we present results on the analysis of the subsystem 1 dynamics without coupling. We analytically determine the equilibrium points and use tables to classify them, according to the trace and the determinant of the Jacobian matrix. We present the different tables as a function of the parameter *m*.

#### Analysis of subsystem 1

##### Subsystem 1 equilibrium points and stability

We analytically find the equilibrium points (*x*_1_, *y*_1_) by solving the following equations:

(36) $$\{\begin{array}{l} c-dx_{1}^{2}-f_{1}(x_{1},0)-z+I_{ext_{1}}=0\\ y_{1}=c-dx_{1}^{2} \end{array}$$

where *x*_1_ is a solution of:

(37) $$c+(b-d)x_{1}^{2}-ax_{1}^{3}-z+I_{ext_{1}}=0\;\text{if}\;x_{1}<0$$

(38) $$c-dx_{1}^{2}+(m+0.6{\left(z-4\right)}^{2})x_{1}-z+I_{ext_{1}}=0\;\text{if}\;x_{1}\geq 0.$$

Let $\delta_{-}$ be the discriminant of Equation 37 and $\delta_{+}$ be the discriminant of Equation 38.

- If $\delta_{-}>0$, Equation 37 has one solution.
- If $\delta_{-}=0$, Equation 37 has one solution.
- If $\delta_{-}<0$, Equation 37 has three solutions.

The solutions are equilibrium points if $x_{1}<0$.

- If $\delta_{+}\geq 0$, Equation 38 has two solutions, $x_{1}^{1}$ and $x_{1}^{2}$ are written as:
  (39) $$\{\begin{array}{l} x_{1}^{1}=\frac{R-\sqrt{\delta_{+}}}{2d}\\ x_{1}^{2}=\frac{R+\sqrt{\delta_{+}}}{2d}\\ R=m+0.6{\left(z-4\right)}^{2}\\ \delta_{+}=R^{2}+20(1-z+I_{ext_{1}}). \end{array}$$

Let *X * = * z*−4, then z∈]−∞,min(X)+4]∪[max(X)+4,+∞[ and *X* is a solution of

(40) $$X^{4}+pX^{2}+qX+w=0$$

where

(41) $$\{\begin{array}{l} p=\frac{10m}{3}\\ q=-500/9\\ w=(25/9)(m^{2}+20(I_{ext_{1}}-3)). \end{array}$$

**Figure 38.**
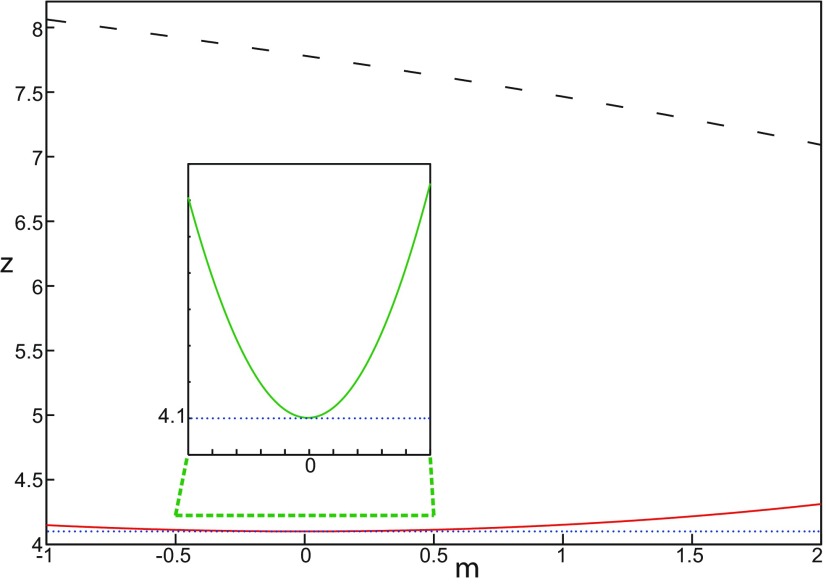
Finding min(*X*) + 4 (red solid) and max(*X*) + 4 (black dashed) with respect to *m*. *X* is a solution of Equation 41. Blue (dotted) curve corresponds to $I_{ext1}+1$. The equilibrium points of the subsystem 1 ($x_{1}\geq 0$) exist according to Equation 42.

We find min(X)+4 and max(X)+4 as *m* varies in Figure 38. Solutions of Equation 38 are equilibrium points if $x_{1}^{1}\geq 0$ and $x_{1}^{2}\geq 0$. Then an equilibrium point *x*_1_ (*x*_1_ ≥ 0) exists if:

(42) $$\{\begin{array}{ll} z\in ]-\infty ,I+1]\cup [max(X)+4,+\infty [ & \forall m\leq 0\\ z\in ]-\infty ,min(X)+4]\cup [max(X)+4,+\infty [ & \forall m>0 \end{array}$$

where *X* is a solution of Equations 40 and 41.

To determine the equilibrium points stability, we analyze the eigenvalues of the following Jacobian matrix *J*:

$$J(x_{1})=\left|\begin{array}{cc} E(x_{1},z) & 1\\ -2dx_{1} & -1 \end{array}\right|$$

where

(43) $$E(x_{1},z)=\{\begin{array}{ll} -3ax_{1}^{2}+2bx_{1} & \;\text{if}\;x_{1}<0\\ m+0.6{\left(z-4\right)}^{2} & \;\text{if}\;x_{1}\geq 0. \end{array}$$

*J* is defined at the equilibrium point (*x*_1_, *y*_1_). To find the eigenvalues of *J*, we solve the characteristic equation:

(44) $$det(J(x_{1})-\lambda I)=\lambda^{2}-Tr(J)\lambda +Det(J)$$

where *Tr*(*J*) and *Det*(*J*) are the trace and the determinant of the matrix *J*, respectively.

The trace and the determinant of *J* are given by:

(45) $$Tr(J)=E(x_{1},z)-1$$

(46) $$Det(J)=2dx_{1}-E(x_{1},z).$$

An equilibrium point is stable if all the real parts of the eigenvalues of J are negative (Izhikevich, 2007). We can classify the equilibrium points according to the trace and the determinant of *J* (Izhikevich, 2007). We determine the intervals on which the *Tr*(*J*) and *Det*(*J*) are negative and positive.

The roots of *Tr*(*J*) are as follows:

(47) $$\{\begin{array}{l} \begin{array}{l} x_{1}(Tr_{1})=(b-\sqrt{b^{2}-3a})/3a\\ x_{1}(Tr_{2})=(b+\sqrt{b^{2}-3a})/3a \end{array}\}\text{if}\;x_{1}<0\\ \begin{array}{l} z(Tr_{1})=4-\sqrt{\left(5/3)(1-m\right)}\\ z(Tr_{2})=4+\sqrt{\left(5/3)(1-m\right)} \end{array}\}\text{if}\;x_{1}\geq 0,\forall m\leq 1. \end{array}$$

The roots of *Det*(*J*) are as follows:

(48) $$\left\{\begin{array}{c} \begin{array}{l} x_{1}(Det_{1})=\frac{2(b-d)}{3a} \end{array}\}\;\text{if}\;x_{1}<0\\ z(Det_{1})=\left\{\begin{array}{ll} 1+I_{ext_{1}} & \;\text{if}\;m\leq 0\\ min(X)+4 & \;\text{if}\;m>0 \end{array}\right\}\text{if}\;x_{1}\geq 0\\ z(Det_{2})=max(X)+4,\forall m \end{array}\right.$$

where *X* is a solution of Equations 40 and 41.

We conclude that an equilibrium point *x*_1_ (*x*_1_ ≥ 0) exists (see Eq. 42) if:

(49) $$z\in ]-\infty ,z(Det_{1})]\cup [z(Det_{2}),+\infty [.$$

We find the stability of equilibrium points $\forall z\in ]-\infty ,z(Det_{1})]$ and $\forall z\in [z(Det_{2}),+\infty [$ in Tables 2–Tables 5, depending on *m*.

**Table 1:** List of acronyms

DB	Depolarization block
Det	Determinant
EEG	Electroencephalographic
H	Hopf bifurcation
HB	Homoclinic bifurcation
J	Jacobian matrix
LC	Limit cycle attractor in the Epileptor
NS	Normal state
RSE	Refractory status epilepticus
S	Separatrix, Saddle periodic orbit
SD	Spreading depression
SLC	Small limit cycle in the Epileptor
SLC1	Small limit cycle in the fast-slow subsystem
SLE	Seizure-like event
SN	Saddle-node bifurcation
SNIC	Saddle-node on invariant circle bifurcation
SNPO	Saddle-node of periodic orbits bifurcation
SWEs	Sharp-wave events
Tr	Trace

**Table 2:** Equilibrium points stability, $\forall z\in ]-\infty ,z(Det_{1})],\;\forall m\leq 0$

*x*_1_	−∞	$\frac{2(b-d)}{3a}$		0		*α*		+∞
Det	+		−		+		+	
Tr	−		−		−		+	
Stability	Stable focus/node		Saddle		Stable focus/node		Unstable focus/node	

**Table 3:** Equilibrium points stability, $\forall z\in ]-\infty ,z(Det_{1})],\;\forall 0<m\leq 1$

*x*_1_	−∞	$\frac{2(b-d)}{3a}$		0		*β*		*α*		+∞
Det	+		−		−		+		+	
Tr	−		−		−		−		+	
Stability	Stable focus/node		Saddle		Saddle		Stable focus/node		Unstable focus/node	

**Table 4:** Equilibrium points stability, $\forall z\in ]-\infty ,z(Det_{1})],\;\forall m>1$

*x*_1_	−∞	$\frac{2(b-d)}{3a}$		0		*β*	+∞
Det	+		−		−		+
Tr	−		−		+		+
Stability	Stable focus/node		Saddle		Saddle		Unstable focus/node

**Table 5:** Equilibrium points stability, $\forall z\in [z(Det_{2}),+\infty [,\forall m$

$x_{1}$	**0**	γ		+∞
Det	−		+	
Tr	+		+	
Equilibrium point stability	Saddle		Unstable focus/node	

**Table 6:** Stability of subsystem 2 equilibrium points, $\forall \tau_{2}\geq 1,\;\forall a_{2}>1$

*x*_2_	−∞	$x_{2}(Det)$		$x_{2}(Tr_{1})$		-0.25		$x_{2}(Tr_{2})$		+∞
Det	+		−		−		+		+	
Tr	−		−		+		+		−	
Stability	Stable focus		Saddle		Saddle		Unstable focus		Stable focus	
	/node						/node		/node	

We graphically determine the equilibrium points in a phase plane. The equilibrium points lie at the intersection of the *x*_1_- and *y*_1_-nullclines. The *x*_1_-nullcline (${\dot{x}}_{1}=0$) corresponds to a cubic curve for *x*_1_ < 0 and a straight line for *x*_1_ ≥ 0.

The cubic curve is written in the form:

(50) $$y_{1}=ax_{1}^{3}-bx_{1}^{2}+z-I_{ext_{1}}$$

and the straight line is written as follows:

(51) $$y_{1}=-(m+0.6{\left(z-4\right)}^{2})x_{1}+z-I_{ext_{1}}.$$

The *y*_1_-nullcline (${\dot{y}}_{1}=0$) corresponds to a parabola given by:

(52) $$y_{1}=c-dx_{1}^{2}.$$

**Figure 39.**
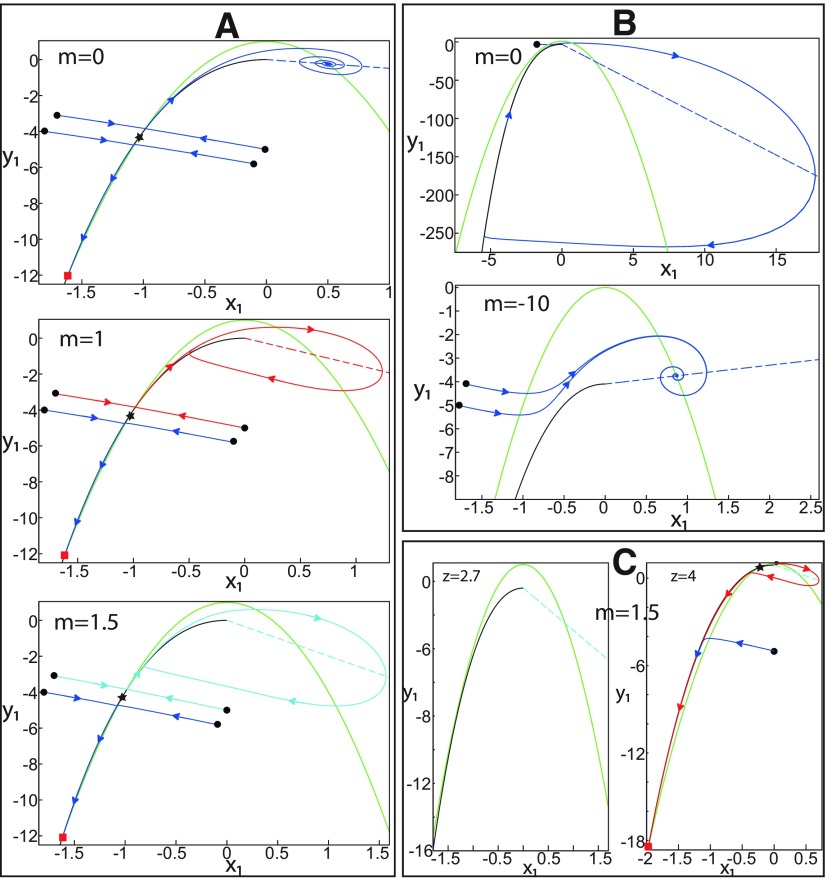
The (*x*_1_, *y*_1_) phase plane of the subsystem 1. Possible intersections of *x*_1_- (black cubic curve $\forall x_{1}<0$ and dashed straight line $\forall x_{1}\geq 0$) and *y*_1_- (green parabola) nullclines depending on *z* and *m*. Trajectories are plotted without noise starting from different initial conditions (black dot). The arrows indicate the direction of trajectories. The equilibrium points where they exist are labeled by red squares for stable nodes, and black stars for saddles. ***A***, *z * = * *3.1, three equilibrium points coexist: a stable node (bottom), a saddle (middle), and a stable focus for *m * = * *0. The stable focus becomes unstable when *m * = * *1 and *m * = * *1.5, surrounded by a stable limit cycle. The limit cycle radius increases as *m* is increased. ***B***, *z * = * *0, one equilibrium point exists which is an unstable focus surrounded by a stable limit cycle for *m * = * *0, and a stable focus for *m* = −10. ***C***, *m * = * *1.5, one equilibrium point exists for *z * = * *2.7, and three equilibrium points exist for *z * = * *4: a stable node, a saddle, and an unstable focus. The stable limit cycle does not surround the unstable focus, and then is broken through a homoclinic bifurcation.

We plot the phase plane of the subsystem 1 in Figure 39*A* for *z * = * *3.1 and in Figure 39*B* for *z * = * *0. We consider only the negative *x*_1_-axis for the cubic curve, and plot a straight line of the *x*_1_-nullcline ($\forall x_{1}\geq 0$) for different *m*.

When *z * = * *3.1, then $z-I_{ext_{1}}=0$ (Fig. 39*A*). We plot a straight line of the *x*_1_-nullcline for *m * = * *0, *m *=* * 1, and *m * =* * 1.5. Then the *x*_1_- and *y*_1_-nullclines intersect at three points for each *m* value. We plot four trajectories with different initial conditions (*i*_1_, *i*_2_, *i*_3_, and *i*_4_). Trajectories *i*_1_ and *i*_2_ converge to one equilibrium point, which is a stable node. Trajectories *i*_3_ and *i*_4_ converge to one equilibrium point, which is a stable focus when *m * = * *0 (Fig. 39*A*, *m * = * *0). By increasing (*m * = * *1 and *m * = * *1.5), the stable focus loses its stability, and the equilibrium point is an unstable focus. Trajectories *i*_3_ and *i*_4_ converge to a stable limit cycle, which surrounds the unstable focus. The radius of the limit cycle increases as *m* increases (Fig. 39*A*, *m * = * *1 and *m * = * *1.5).

We conclude that when *z * = * *3.1, three equilibrium points coexist. The first one is a stable node and the second one is a stable or an unstable focus, depending on *m*. The third equilibrium point is a saddle. The stable manifold of the saddle corresponds to a separatrix between the first and second equilibrium point. The subsystem 1 undergoes a supercritical Andronov–Hopf bifurcation, *H*, as *m* increases at *m*(*H*)* * = * *0.514 [i.e., solution of *Tr*(*J*)* * = * *0; Eq. 45].

When *z * = * *0, then $z-I_{ext_{1}}$ decreases and the *x*_1_-nullcline moves downward in the phase plane (*m* = −10; Fig. 39*B*). We plot a straight line of the *x*_1_-nullcline ($\forall x_{1}\geq 0$) for *m * = * *0 and *m* = −10. Then the *x*_1_- and *y*_1_-nullclines intersect at one equilibrium point, which is a stable focus for *m* = −10 and an unstable focus for *m * = * *0 (Fig. 39*B*). The stable node and saddle disappear. We plot trajectories in the phase plane, which converge to a stable focus for *m* = −10 and to a stable limit cycle for *m * = * *0. The limit cycle surrounds the unstable focus. The subsystem 1 undergoes a supercritical Andronov–Hopf bifurcation, *H*, at *m*(*H*) = −8.6 [i.e., solution of *Tr*(*J*)* * = * *0; Eq. 45].

We now fix *m* on two values to identify the equilibrium points as *z* varies.

- Let *m* = 0, three equilibrium points coexist when *z* = 3.1: a stable node, a saddle, and a stable focus (Fig. 39*A*). When *z* = 0, then only an unstable focus exists (Fig. 39*B*). Hence, as *z* decreases, a stable focus becomes unstable, and the subsystem 1 undergoes a supercritical Andronov–Hopf bifurcation, *H*. Moreover, the stable node and saddle disappear when *z* = 0 (Fig. 39*B*). In fact, the stable node and saddle approach each other as *z* decreases and coalesce at *z* ≈ 2.9 (figure not shown), which corresponds to $z(x_{1}(Det_{1}))$ (see Eq. 48). Decreasing *z* further, the stable node and saddle disappear through a saddle-node bifurcation.
- Let *m* = 1.5, three equilibrium points coexist when *z* = 3.1: a stable node, a saddle, and an unstable focus (Fig. 39*A*). When *z* decreases to *z* ≈ 2.9, then a saddle-node bifurcation occurs (figure not shown). When *z* = 2.7, the stable node and saddle disappear (Fig. 39*C*). In contrast, when *z* = 4 (≥ 3.1), three equilibrium points coexist: a stable node, a saddle, and an unstable focus (Fig. 39*C*). A stable limit cycle surrounds the unstable focus when *z* = 3.1 (Fig. 39*A*), and it does not when *z* = 4 (Fig. 39*C*). In fact, the stable limit cycle approaches as *z* increases to the saddle, touches the saddle, and then terminates. Hence, when *z* = 4, the stable limit cycle disappears through a homoclinic bifurcation, and the trajectories converge to a stable node.

We conclude that the stability of equilibrium points depends on *z* and *m*. In addition, we find three bifurcation types: a saddle-node bifurcation, a homoclinic bifurcation, and a supercritical Hopf bifurcation.

##### Subsystem 1 bifurcation diagram

**Figure 40.**
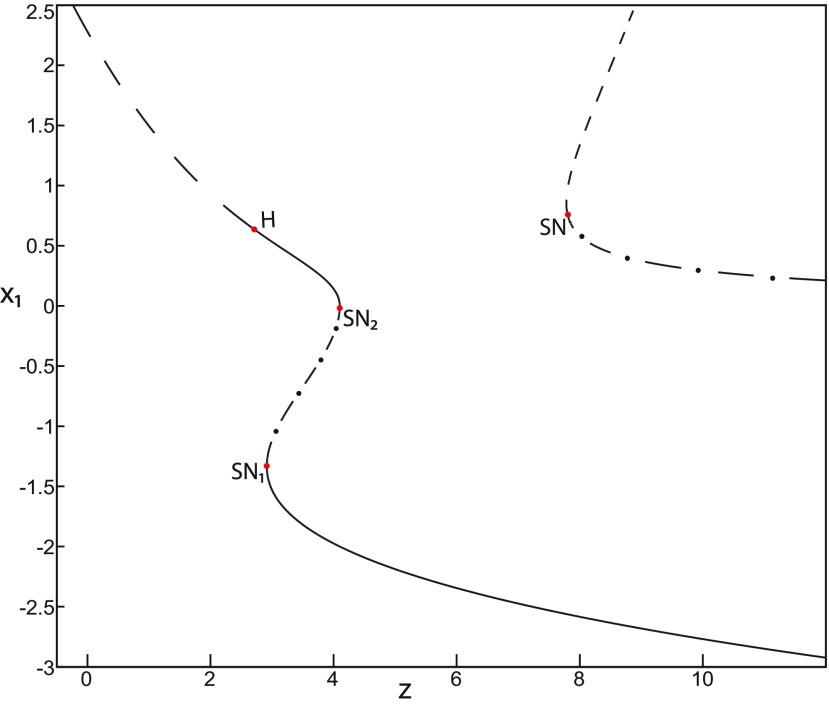
The (*z*, *x*_1_) subsystem 1 bifurcation diagram with respect to *z* when *m * = * *0. On the left, the plot shows a *Z*-shaped curve ∀z<z(SN). *Z*-lower (solid) and *Z*-middle (dash-dotted) branches consist of stable nodes and saddles, respectively. *Z*-upper (solid) sub-branch consists of stable foci and *Z*-upper (dashed) sub-branch consists of unstable foci. The two sub-branches are separated by a Hopf bifurcation *H*. The *Z*-lower and *Z*-middle branches collide as *z* decreases in a saddle-node bifurcation SN_1_. The *Z*-upper and *Z*-middle branches collide as *z* increases in a saddle-node bifurcation SN_2_. The *Z*-shaped curve comprises two branches ∀z≥z(SN): one (dashed) consists of unstable nodes and another (dash-dotted) consists of saddles. Decreasing *z*, they collide in a saddle-node bifurcation, SN.

We find the equilibrium points as *z* varies in a (*z*, *x*_1_) bifurcation diagram plotted in Figure 40 for *m * = * *0. The (*z*, *x*_1_) curve comprises two geometrical shapes. The right one comprises two branches: the lower (dash-dotted) branch consists of saddles; and the upper (dashed) branch consists of unstable foci. When decreasing *z*, the lower and upper branches collide in a saddle-node bifurcation, SN. The left geometrical shape corresponds to a *Z*-curve known from fast-slow subsystems (Ermentrout and Terman, 2010). Left and right shapes are separated by SN_2_ and SN, such that $z(SN_{2})=I_{ext_{1}}+1$ and z(SN)=max(X)+4 (Eq. 42). The discriminant $\delta_{+}$ of Equation 38 is negative between SN_2_ and SN. Lower and upper branches (right shape) consist of saddles and unstable nodes, respectively ∀m. Hence, in what follows, we plot only the *Z*-curve (left shape) in the next bifurcation diagrams.

**Figure 41.**
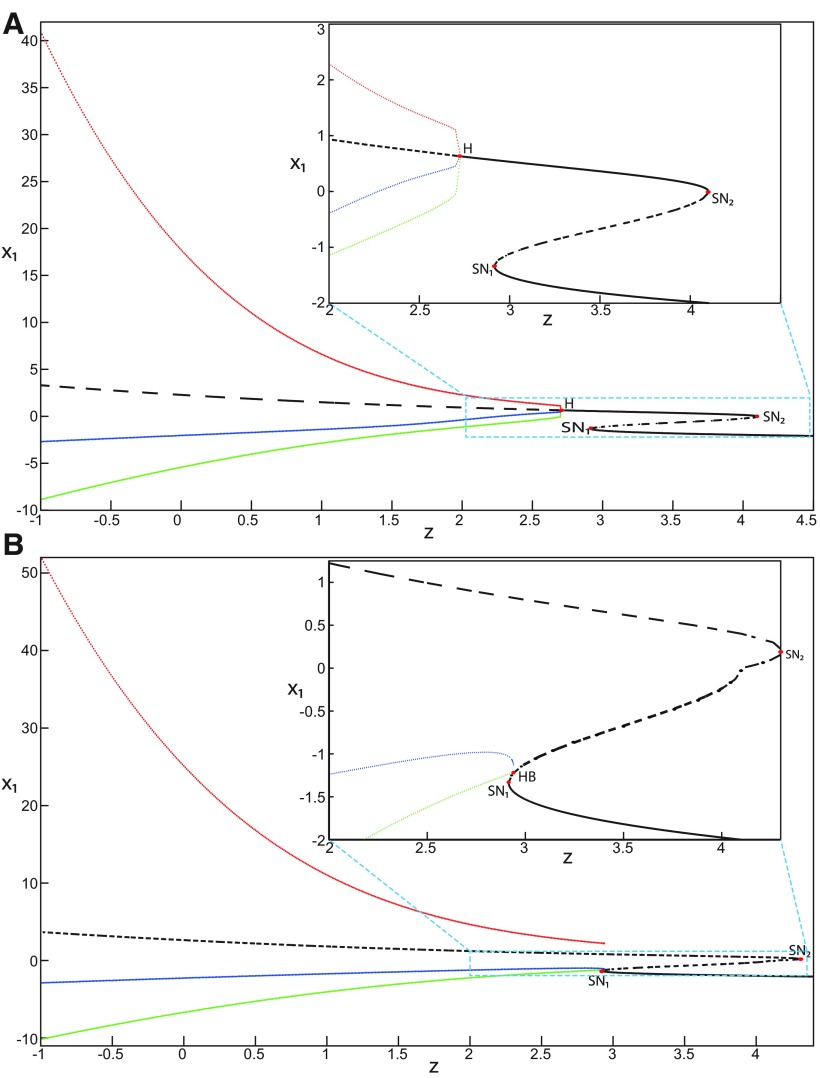
***A***, ***B***, The ($z,x_{1}$) subsystem 1 bifurcation diagram with respect to *z* (z<z(SN)), when *m * = * *0 (***A***) and *m * = * *2 (***B***). *Z*-lower (solid) and *Z*-middle (dash-dotted) branches consist of stable nodes and saddles, respectively. *Z*-upper branch consists of unstable foci (***B***), and it is divided into two sub-branches separated by a Hopf bifurcation, *H* (***A***): one (solid) consists of stable foci and another (dashed) consists of unstable foci. The *Z*-lower and *Z*-middle branches collide as *z* decreases in a saddle-node bifurcation SN_1_. The *Z*-upper (solid for ***A*** and dashed for ***B***) and *Z*-middle branches collide as *z* increases in a saddle-node bifurcation SN_2_. A stable limit cycle ends at a Hopf bifurcation (***A***) and at a homoclinic bifurcation (***B***). The curves $max(x_{1}),\;min(x_{1})$ and $<x_{1})>$ correspond to the maximum, minimum, and averaged values along periodic orbits.

We plot a (*z*, *x*_1_) bifurcation diagram for *m * = * *0 in Figure 41*A*. The geometrical shape of the bifurcation diagram corresponds to a *Z*-curve, which comprises four branches. *Z*-lower (solid) and *Z*-middle (dash-dotted) branches consist of stable nodes and saddles, respectively. Decreasing *z*, *Z*-lower and *Z*-middle branches collide in a saddle-node bifurcation point, SN_1_. The *Z*-middle branch acts as a separatrix between *Z*-lower and *Z*-upper branches.

The *Z*-upper branch comprises two sub-branches separated by a Hopf bifurcation, *H*: one sub-branch (solid) consists of stable foci and another (dashed) consists of unstable foci (Fig. 41*A*). The first sub-branch corresponds to a nonoscillatory state, and the second sub-branch corresponds to an oscillatory state. The subsystem 1 then switches as *z* decreases from nonoscillatory to oscillatory states.

An unstable focus is surrounded by a stable limit cycle, which is limited by red ($max(x_{1})$) and green ($min(x_{1})$) curves. The stable limit cycle radius increases as *z* is decreased. The stable limit cycle terminates in a Hopf bifurcation, *H*, written in the form:

(53) $$z(H)=4-\sqrt{\left(5/3)(1-m\right)}$$

which corresponds to $z(Tr_{1})$ (see Eq. 47), $\forall x_{1}\geq 0$). The Hopf bifurcation point *z*(*H*) exists for *m * ≤* * 1 moving rightward (leftward) on the *Z*-upper curve as *m* increases (decreases), and does not exist for *m * >* * 1.

We plot a (*z*, *x*_1_) bifurcation diagram for *m * = * *2 in Figure 41*B*. *Z*-lower (solid) and *Z*-middle (dash-dotted) branches consist of stable nodes and saddles, respectively. *Z*-upper (dashed) branch consists only of unstable foci, which are surrounded by stable limit cycles. The stable limit cycle amplitude is limited by red ($max(x_{1})$) and green ($min(x_{1})$) curves. The stable limit cycle terminates as *z* increases in a homoclinic bifurcation HB. Decreasing *z*, *Z*-lower and *Z*-middle branches collide in a saddle-node bifurcation SN_1_. Increasing *z*, *Z*-upper and *Z*-middle branches collide in a saddle-node bifurcation SN_2_ (Fig. 41*B*, inset).

**Figure 42.**
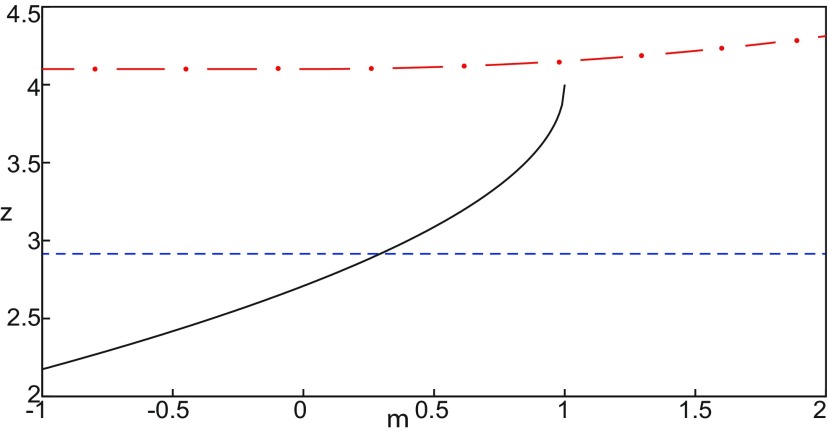
Finding bifurcation points with respect to *m*. SN_1_ (blue dashed) and SN_2_ (red dash-dotted) correspond to saddle-node bifurcations, and *H* (black solid) to a Hopf bifurcation. $I_{ext1}=3.1$.

SN_1_ and SN_2_ correspond to saddle-node bifurcation points, and HB corresponds to a homoclinic bifurcation point. SN_1_ and SN_2_ points are given by the following:

(54) $$z(SN_{1})=-0.1852+I_{ext_{1}}$$

which corresponds to $z(x_{1}(Det_{1}))$ (see Eq. 48), $\forall x_{1}<0$),

(55) $$z(SN_{2})=\{\begin{array}{ll} 1+I_{ext_{1}} & \;\text{if}\;m\leq 0\\ min(X)+4 & \;\text{if}\;m>0 \end{array}$$

where *X* is a solution of Equations 40 and 41. We find saddle-node bifurcation points SN_1_ and SN_2_, and a Hopf bifurcation point, H, in Figure 42 as *m* varies. Figure 42 shows the following three cases:

- For *m* ≤ 0.29, then $z(H)<z(SN_{1})$, and the subsystem 1 is bistable on [SN_1_, SN_2_].
- For 0.29<m<1, then $z(H)>z(SN_{1})$, and the subsystem 1 is bistable on [SN_1_, SN_2_].
- For *m* > 1, then H does not exist, and the subsystem 1 is bistable on [SN_1_, HB].

##### Fast-slow subsystem equilibrium points

We analytically find the equilibrium points by solving the Equations 37 and 38, where *z* is a solution of $\dot{z}=0$ (Eq. 18). We determine the stability of the equilibrium points by analyzing the Jacobian matrix *J* written as:

$$J(x_{1})=\left|\begin{array}{ccc} E(x_{1},z) & 1 & K(x_{1},z)\\ -2dx_{1} & -1 & 0\\ rs & 0 & L(z) \end{array}\right|$$

where

(56) $$E(x_{1},z)=\{\begin{array}{ll} -3ax_{1}^{2}+2bx_{1} & \;\text{if}\;x_{1}<0\\ m+0.6{\left(z-4\right)}^{2} & \;\text{if}\;x_{1}\geq 0 \end{array}$$

(57) $$K(x_{1},z)=\{\begin{array}{ll} -1 & \;\text{if}\;x_{1}<0\\ 1.2(z-4)x_{1}-1 & \;\text{if}\;x_{1}\geq 0 \end{array}$$

(58) $$L(z)=\{\begin{array}{ll} -r(1+0.7z^{6}) & \;\text{if}\;z<0\\ -r & \;\text{if}\;z\geq 0. \end{array}$$

*J* is defined at the equilibrium point (*x*_1_, *y*_1_, *z*). We find the eigenvalues of *J* by solving the characteristic equation:

(59) $$det(J(x_{1})-\lambda I)=\lambda^{2}-Tr(J)\lambda +Det(J).$$

The trace, *Tr*(*J*), and the determinant, *Det*(*J*), of *J* are given by:

(60) $$Tr(J)=E(x_{1},z)-1+L(z)$$

(61) $$Det(J)=2dx_{1}L(z)-E(x_{1},z)L(z)+rsK(x_{1},z).$$

We graphically determine the equilibrium points using the nullclines. The *x*_1_-nullcline corresponds to the Equations 50 and 51, and the *y*_1_-nullcline corresponds to Equation 52.

The *z*-nullcline ($\dot{z}=0$, Eq. 18) corresponds to a straight line for *z * ≥ * *0 written in the form:

(62) $$x_{1}(z)=(z/s)+x_{0}$$

and to a curve for *z *<* *0 written as:

(63) $$x_{1}(z)=((z+0.1z^{7})/s)+x_{0}.$$

**Figure 43.**
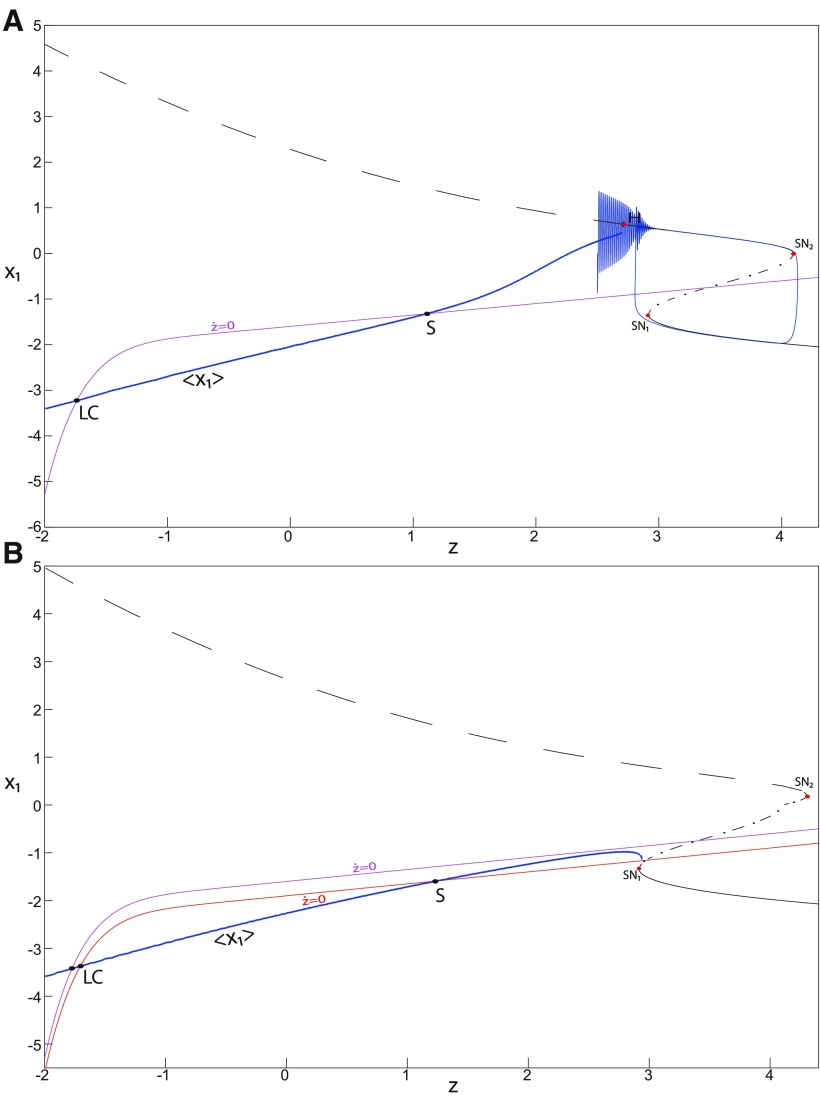
Finding the equilibrium points and periodic orbits of the fast-slow subsystem by using the (*z*, *x*_1_) bifurcation diagram of the subsystem 1 shown in Figure 41. The *z*-nullcline ($\dot{z}=0$) intersects the *Z*-shaped curve at equilibrium points, and the $<x_{1}>$ curve at periodic orbits. ***A***, ***B***, *m * = * *0 and *x*_0_ = −1.6 (***A***), *m * = * *2 and *x*_0_ = −1.6 (top, purple; ***B***) or *x*_0_ = −1.9 (bottom, red; ***B***). LC and S are stable and saddle periodic orbits, respectively.

The equilibrium points lie at the intersection of the *z*-, *x*_1_-, and *y*_1_-nullclines. We graphically find the equilibrium points using the bifurcation diagram of the subsystem 1 (Fig. 41). We plot the *Z*-curve using the intersection of the *x*_1_- and *y*_1_-nullclines for each *z*. Therefore, the equilibrium points of the fast-slow subsystem lie at the intersection of the *z*-nullcline and the *Z*-curve (Fig. 43). A bifurcation diagram of the subsystem 1 is plotted in Figure 43*A* for *m * = * *0 and in Figure 43*B* for *m * = * *2. When *x*_0_ = −1.6, then the *z*-nullcline is at the *Z*-middle branch for *m * =* * 0 (Fig. 43*A*) and *m * = * *2 (Fig. 43*B*), and then only a saddle exists. When *x*_0_ = −1.9, then the *z*-nullcline is at the *Z*-middle branch for *m * =* * 2, and the equilibrium point is a saddle (Fig. 43*B*).

**Figure 44.**
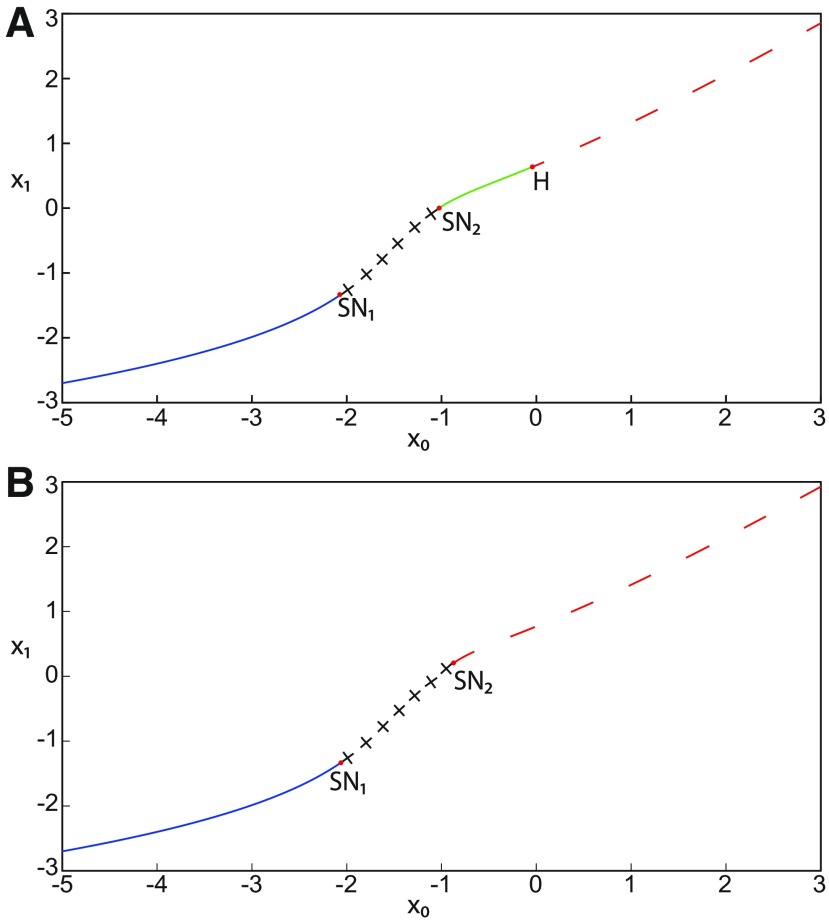
***A***, ***B***, Finding equilibrium points of the fast-slow subsystem with respect to *x*_0_, for *m * = * *0 (***A***) and *m * = * *2 (***B***). Blue (bottom solid), black (plus sign markers), red (dashed), and green (top solid) branches consist of stable nodes, saddles, unstable foci, and stable foci, respectively. SN_1_ and SN_2_ correspond to saddle-node bifurcation points and *H* to a Hopf bifurcation point.

The *z*-nullcline moves downward (*x*_0_ decreases) or upward (*x*_0_ increases) in the bifurcation diagram, and intersects the (*z*, *x*_1_) curve at different points. To determine these points as *x*_0_ varies, we plot a (*x*_0_, *x*_1_) diagram in Figure 44*A* for *m * = * *0 and in Figure 44*B* for *m * = * *2. The curve consists of equilibrium points of the fast-slow subsystem. Blue (bottom solid), black (plus sign markers), red (dashed), and green (top solid) branches consist of stable nodes, saddles, unstable foci, and stable foci, respectively. The branch of stable foci exists for *m * = * *0 but does not for *m * = * *2. SN_1_ and SN_2_ correspond to saddle-node bifurcation points, and *H* to a Hopf bifurcation point (Fig. 44).

##### Fast-slow subsystem behavior

**Figure 45.**
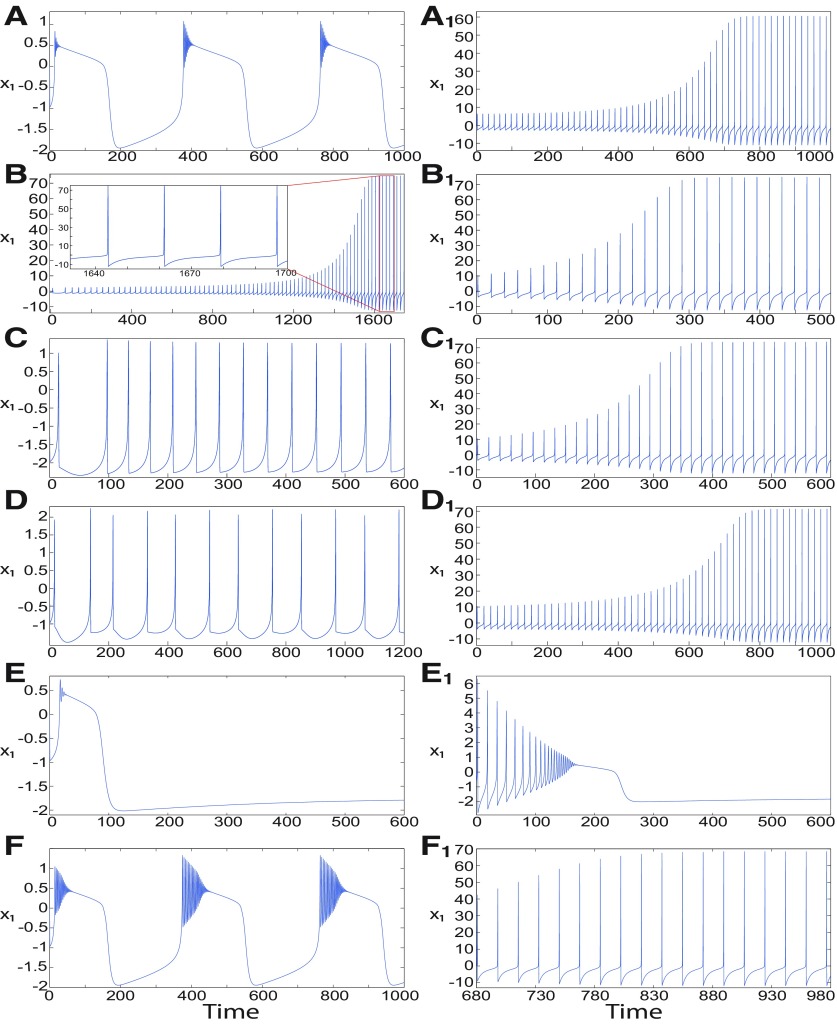
Time series of the fast-slow subsystem as *m* and *x*_0_ vary. Initial conditions are [0 −5 3] for left column and [0 −5 1] for right column. *r * = * *0.002. ***A***, ***A_1_***, *m * = * *0, *x*_0_ = −1.6, transitions between upper and lower states occur through a fold/fold bifurcation for ***A***; LC exists for ***A_1_***. ***B***, ***B_1_***, *m * = * *2, *x*_0_ = −1.6, only LC exists. ***C***, ***C_1_***, $m=2,x_{0}=-1.7$, SLC_1_ exists for ***C*** and LC for ***C_1_***. ***D***, ***D_1_***, *m * = * *2, $x_{0}=-1.9$, transitions between upper and lower states occur through a fold/homoclinic bifurcation for ***D***; LC exists for ***D_1_***. ***E***, ***E*_1_**, $m=0,\;x_{0}=-2.6$, only a resting state exists. ***F***, ***F_1_***, *m * = * *0.5, $x_{0}=-1.6$, transitions between upper and lower states occur through a fold/Hopf bifurcation for ***F***; LC exists for ***F_1_***.

**Figure 46.**
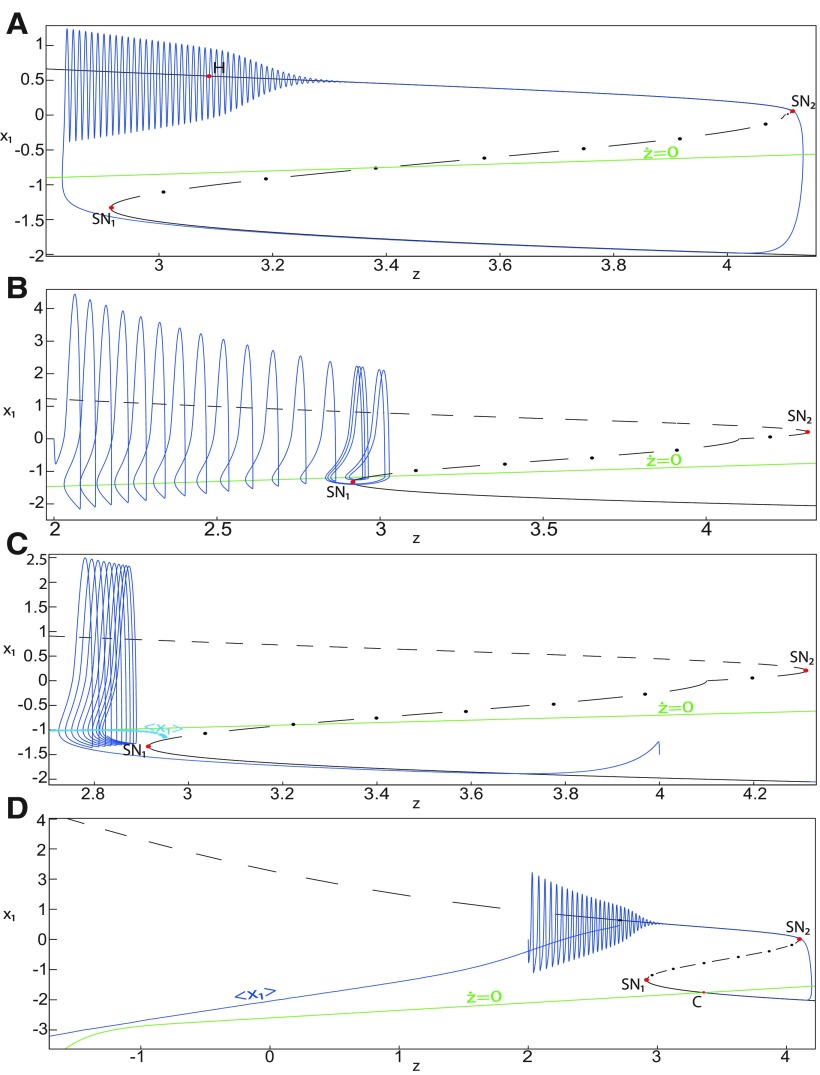
The ($z,x_{1}$) subsystem 1 bifurcation diagram with respect to *z* as *m* and *x*_0_ vary. ***A***, *m * = * *0.5 and *x*_0_ = −1.6, transitions between upper and lower branches occur through a fold/Hopf bifurcation. ***B***, *m * = * *2 and *x*_0_ = −1.9, transitions between upper and lower branches occur through a fold/homoclinic bifurcation. ***C***, *m * = * *2 and *x*_0_ = −1.7, the fast-slow subsystem stabilizes before a homoclinic bifurcation occurs, giving rise to the stable limit cycle SLC_1_. ***D***, *m * = * *0 and *x*_0_ = −2.6, the fast-slow subsystem stabilizes its stable node, which is the equilibrium point of the NS. The *z*-nullcline and $<x_{1}>$ curve do not intersect, hence NS exists and LC does not. *r * = * *0.0006, I.C = [−0.5 −5 2.831], and *T_s_* = [0:0.01: 1120] for ***A***; *r * = * *0.002, I.C = [0 −5 2], and *T_s_* = [0:0.01: 1300] for ***B***; *r * = * *0.003, I.C = [−1.5 −5 4], and *T_s_* = [0:0.01: 600] for ***C***; and *r * = * *0.002, I.C = [0 −5 2], and *T_s_* = [0:0.01: 1000] for ***D***.

The stability of equilibrium points depends on *m* and *x*_0_. Different behavioral patterns are discovered as *m* and *x*_0_ vary: a resting state, a fold/fold bifurcation, a fold/homoclinic bifurcation, a fold/Hopf bifurcation and a periodic solution (Fig. 45). To observe this dynamical change, we integrated the fast-slow subsystem equations for different *m* and *x*_0_. We plot time series of the fast-slow subsystem in Figure 45, and trajectories in Figure 46, as *m* and *x*_0_ vary.

*NS:* We plot a (*z*, *x*_1_) bifurcation diagram for *m * = * *0 in Figure 46*D*. Let *x*_0_ = −2.6, the *z*-nullcline is at the *Z*-lower branch. The equilibrium point is a stable node. When a trajectory is at the *Z*-upper (dashed) sub-branch, then it exhibits an oscillatory solution, which terminates as *z* increases in a Hopf bifurcation *H*. Then, the trajectory exhibits a nonoscillatory solution on the *Z*-upper (solid) sub-branch, which terminates as *z* increases in a saddle-node bifurcation point, SN_2_, and the trajectory switches to the *Z*-lower branch. Decreasing *z*, the trajectory continues to *C* at which *z* stabilizes. Time series are plotted in Figure 45, *E* and *E1*.

If *m * = * *2, then the trajectory exhibits an oscillatory solution on the *Z*-upper branch, which terminates as *z* increases in a homoclinic bifurcation and the trajectory switches to the *Z*-lower branch. The trajectory continues as *z* decreases to *C*, at which *z* stabilizes (figure not shown).

*Fold/Hopf bifurcation:* We plot a (*z*, *x*_1_) bifurcation diagram for *m * = * *0.5 in Figure 46*A*. When *m * = * *0.5, then $z(H)>z(SN_{1})$ (Fig. 42). Let *x*_0_ = −1.6, the *z*-nullcline is at the *Z*-middle branch. The equilibrium point is a saddle. When a trajectory is at the *Z*-lower branch, the stable node disappears as *z* decreases through a saddle-node bifurcation, SN_1_. Then, the trajectory switches to the *Z*-upper (dashed) sub-branch exhibiting an oscillatory solution, which terminates as *z* increases in a Hopf bifurcation, *H*. The trajectory exhibits a nonoscillatory solution on the *Z*-upper (solid) sub-branch, which terminates as *z* increases in a saddle-node bifurcation, SN_2_, and then switches to the *Z*-lower branch. Therefore, the transitions between *Z*-lower and *Z*-upper branches occur through a fold/Hopf bifurcation. Time series are plotted in Figure 45*F*.

*Fold/fold bifurcation:* We plot a (*z*, *x*_1_) bifurcation diagram for *m * = * *0 in Figure 43*A*. When *m * = * *0, then $z(H)<z(SN_{1})$ (Fig. 42). Let *x*_0_ = −1.6, the *z*-nullcline is at the *Z*-middle branch (Fig. 43*A*). The equilibrium point is a saddle. If a trajectory is at the *Z*-upper (dashed) sub-branch, then it exhibits an oscillatory solution, which terminates as *z* increases in a Hopf bifurcation *H*. The trajectory exhibits a nonoscillatory solution on the *Z*-upper (solid) sub-branch, which terminates as *z* increases in a saddle-node bifurcation SN_2_, and then switches to the *Z*-lower branch. Decreasing *z*, the stable node disappears through a saddle-node bifurcation SN_1_ and the trajectory switches to the *Z*-upper (solid) branch, which consists of stable foci. Therefore, the transitions between *Z*-lower and *Z*-upper branches occur through a fold/fold bifurcation. Time series are plotted in Figure 45*A*.

*Fold/homoclinic bifurcation:* We plot a (*z*, *x*_1_) bifurcation diagram for *m * = * *2 in Figures 43*B* and 46*B*. Here, a Hopf bifurcation points does not exist (Fig. 42). Let *x*_0_ = −1.9, the *z*-nullcline is at the *Z*-middle branch. The equilibrium point is a saddle. When a trajectory is at the *Z*-upper (dashed) branch, it exhibits an oscillatory solution, which terminates as *z* increases in a homoclinic bifurcation HB, and then switches to the *Z*-lower branch. Decreasing *z*, the stable node disappears through a saddle-node bifurcation, SN_1_, and the trajectory switches to the *Z*-upper branch. Therefore, the transitions between *Z*-lower and *Z*-upper branches occur through a fold/homoclinic bifurcation. Time series are plotted in Figure 45*D*.

*Periodic solution (SLC_1_):* We plot a (*z*, *x*_1_) bifurcation diagram for *m * = * *2 in Figure 46*C*. Let *x*_0_ = –1.7, the *z*-nullcline is at the *Z*-middle branch. The equilibrium point is a saddle. When a trajectory is at the *Z*-lower branch, the stable node disappears as *z* decreases through a saddle-node bifurcation, SN_1_. Then, the trajectory switches to the *Z*-upper (dashed) branch exhibiting as *z* increases an oscillatory solution which stabilizes at *C*. The *z*-nullcline intersects the $<x_{1}>$-curve at *C*, hence the system solution is periodic and corresponds to a stable limit cycle, with a small amplitude denoted by SLC_1_. Time series are plotted in Figure 45*C*.

Figure 45*B* shows the existence of a periodic solution with a large amplitude when *m * = * *2 and *x*_0_ = −1.6. Here, the equilibrium point is a saddle (Fig. 44*B*). The solution is a stable limit cycle with a fast-slow cyclic behavior, which corresponds to LC.

#### Finding LC

LC and SLC_1_ are stable limit cycles with large and small amplitudes, respectively. Here we show when periodic orbits exist and how they evolve, determining their stability.

##### Sensitivity to initial conditions

The Epileptor model is sensitive to initial conditions. In fact, integrating the fast-slow subsystem in Equations 18–20 from an initial condition results in different behaviors as *m* and *x*_0_ are varied (Fig. 45*A–F*, left). We considered another initial condition and used numerical integration techniques to solve the fast-slow subsystem equations. Figure 45*A1–F1*, (right), shows two different solutions as *m* and *x*_0_ vary. The first solution corresponds to a stable LC and appears in Figure 45, *A1–D1*, and *F1*. The second one corresponds to a normal state, which exists when *x*_0_ = −2.6 (Fig. 45*E*_1_).

We now fix *m* and *x*_0_, and compare the solutions of the fast-slow subsystem as the initial conditions vary:

- When *m* = 0 and *x*_0_ = −1.6, two solutions coexist: a fold/fold bifurcation (Fig. 45*A*) and LC (Fig. 45*A_1_*).
- When *m* = 0.5 and *x*_0_ = −1.6, two solutions coexist: a fold/Hopf bifurcation (Fig. 45*F*) and LC (Fig. 45*F_1_*).
- When *m* = 2 and *x*_0_ = −1.9, two solutions coexist: a fold/homoclinic bifurcation (Fig. 45*D*) and LC (Fig. 45*D_1_*).
- When *m* = 2 and *x*_0_ = −1.7, then two periodic solutions coexist: SLC_1_ (Fig. 45*C*) and LC (Fig. 45*C_1_*).

Therefore, the fast-slow subsystem is sensitive to initial conditions and exhibits a bistability of LC and SLC_1_, a fold/fold bifurcation, a fold/Hopf bifurcation or a fold/homoclinic bifurcation.

##### Finding periodic orbits

For *x*_0_ = −2.6, the normal state exists and LC does not (Fig. 45*E*,*E_1_*). However, when *m * = * *2 and *x*_0_ = −1.6, only the LC exists (Fig. 45*B*,*B_1_*). We explain this using the averaging method. We introduce $<x_{1}>$, which is the average value of *x*_1_-coordinate associated with the subsystem 1, given by:

(64) $$<x_{1}(z)>=\frac{1}{T(z)}\int_{0}^{T(z)}\phi (t;z)\;\text{d}t$$

where $x_{1}=\phi (t;z)$.

We plot $<x_{1}>$ in a (*z*, *x*_1_) bifurcation diagram (Fig. 43). Periodic orbits lie at the intersection of the *z*-nullcline and the $<x_{1}>$-curve. A periodic orbit branch is referred to the *Z*-upper branch, which is surrounded by periodic orbits. This branch and the $<x_{1}>$-curve terminate as *z* increases in a Hopf bifurcation for *m * ≤* * 1 (Fig. 43A) and in a homoclinic bifurcation for *m * >* * 1 (Fig. 43*B*).

Let *x*_0_ = −1.6, Figure 43 shows that the *z*-nullcline intersects the $<x_{1}>$-curve at two points: LC and S when *m * = * *0, and only at LC when *m * = * *2. However, LC and S coexist when *m * = * *2 and *x*_0_ = −1.9 (Fig. 43*B*).

We graphically determine the periodic orbit by using the Pontryagin’s averaging technique (Shilnikov and Kolomiets, 2008). We introduce the following slow averaged nullcline:

(65) $$<\dot{z}>=\{\begin{array}{ll} r(s(<x_{1}>-x_{0})-z-0.1z^{7}) & \;\text{if}\;z<0\\ r(s(<x_{1}>-x_{0})-z) & \;\text{if}\;z\geq 0 \end{array}=0$$

where the periodic orbits correspond to the zeros of the $<\dot{z}>$.

We determine the slow averaged nullcline by substituting the *x*_1_-coordinates by their averaged values in the *z* dynamics Equation 18 and setting the right-hand side of the equation equal to zero (Eq. 65). The slow averaged nullcline depends on *x*_0_ and *z*. We determine the periodic orbit stability by using the following derivative:

(66) $${\frac{d<\dot{z}>}{dz}|}_{z=z^{*}}$$

that represents the dynamics of the averaged equation. A periodic orbit is stable when Equation 66 is negative and is unstable when Equation 66 is positive.

**Figure 47.**
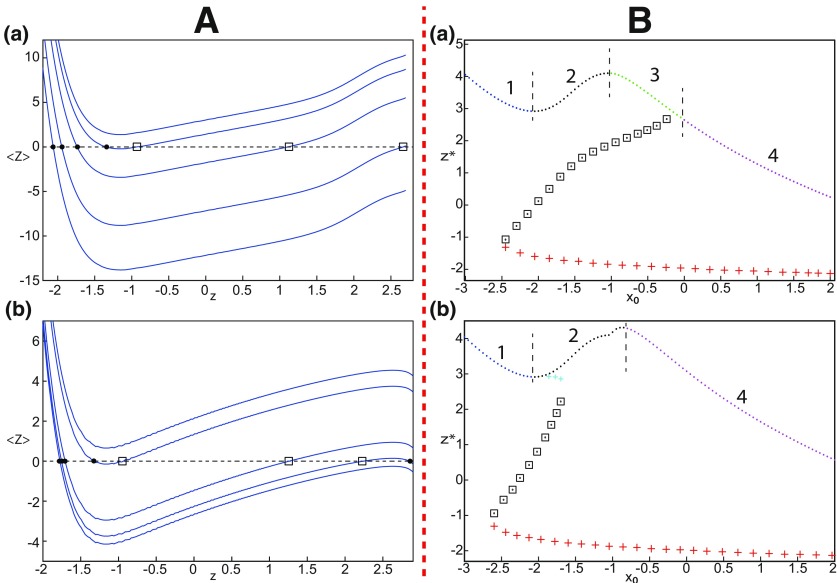
Periodic orbits of subsystem 1. ***A***, Finding periodic orbits with respect to *z* (constant). We plot the graph of the Equation 65 on its right-hand side for different values of *x*_0_ (from bottom to top; *x*_0_ = 1, −0.25, −1.6, −2.4, −2.8) for *m * = * *0 (***a***) and (*x*_0_ = −1.6, −1.7, −1.9, −2.6, −2.8) for *m * = * *2 (***b***). Stable and saddle periodic orbits are labeled as black circles and squares, respectively, which disappear as *x*_0_ decreases through a SNPO bifurcation. ***B***, The fast-slow subsystem parameter space of periodic orbits and equilibrium points with respect to *x*_0_. ***a***, *m * = * *0. ***b***, *m * = * *2. Saddle periodic orbits, *S*, are labeled as black squares (with dot). Stable periodic orbits LC (bottom, red) and SLC_1_ (top, blue) are labeled as (+) markers. Equilibrium points are labeled as dots. Blue (segment 1), black (segment 2), green (segment 3), and purple (segment 4) dots correspond to stable nodes, saddles, stable foci and unstable foci, respectively.

We plot the $<\dot{z}>$-curve in Figure 47*A* for *m * = * *0 (Figure 47*A*,*a*), and *m * = * *2 (Figure 47*A,b*). *z* is a control parameter. The maximal value of *z* corresponds to a Hopf bifurcation point H for m≤1 and to a homoclinic bifurcation point HB for *m *> 1. The periodic orbits are the zeros of the $<\dot{z}>$. A periodic orbit is stable when the $<\dot{z}>$-curve decreases over *z* at the given zero and is unstable when the $<\dot{z}>$ curve increases. Stable and saddle periodic orbits are labeled as black circles and squares respectively.

- Let *m* = 0 (Fig. 47*A*,*a*), $<\dot{z}>$ has a simple zero when *x*_0_ = 1, which is stable. Decreasing *x*_0_ to −0.25, another periodic orbit is born, which is unstable (saddle). When *x*_0_ = −2.45, stable and saddle periodic orbits coalesce forming a saddle-node periodic orbit, which then fades $\forall x_{0}<-2.45$. Stable and saddle periodic orbits disappear through a SNPO bifurcation (Shilnikov and Kolomiets, 2008).
- Let *m* = 2 (Fig. 47*A*,*b*), $<\dot{z}>$ has a simple zero when *x*_0_ = −1.6, which is stable. When $x_{0}=-1.9,$ $<\dot{z}>$ has two zeros: one stable and another saddle. When $x_{0}=-1.7,$ $<\dot{z}>$ has three zeros: two stable periodic orbits separated by a saddle periodic orbit. By decreasing *x*_0_, SNPO bifurcation occurs.

We conclude that LC and S are stable and saddle periodic orbits, respectively (Fig. 43). When *m * = * *2 and *x*_0_ = −1.7, then two zeros of $<\dot{z}>$ are LC and S. The third zero is a stable periodic orbit, which we denote by SLC_1_.

##### Evolution of periodic orbits

LC and S are stable and saddle periodic orbits, respectively. SLC_1_ is a stable periodic orbit, which exists for *m * = * *2. We now analyze how the periodic orbits evolve when *x*_0_ varies slowly. We plot a (*x*_0_, z^*^) bifurcation diagram of periodic orbits in Figure 47*B* for *m * = * *0 (Figure 47*B*,*a*), and *m * = * *2 (Figure 47*B*, *b*). We localize the periodic orbits at z^*^. *x*_0_ is a control parameter.

- Let *m* = 0, only LC exists [red bottom (+) markers] for large *x*_0_. Decreasing *x*_0_, S (black dotted squares) appears. Stable (LC) and saddle (S) periodic orbits approach each other as *x*_0_ decreases, collide in a saddle-node periodic orbit bifurcation, and then fades. LC and S disappear through a SNPO bifurcation (Fig. 47*B*,*a*).
- Let *m* = 2, only LC exists for large *x*_0_. Decreasing *x*_0_, S and SLC_1_ [blue top (+) markers] appear. Hence, LC and SLC_1_ coexist and are separated by S. Decreasing *x*_0_, SLC_1_ disappears. Further decreasing *x*_0_, LC and S disappear through an SNPO bifurcation (Fig. 47*B*,*b*).

We can now explain the solutions found in Figure 45. We plot in the (*x*_0_, z^*^) bifurcation diagram the equilibrium points, labeled as dots (Fig. 47*B*). Blue (segment 1), black (segment 2), green (segment 3), and purple (segment 4) dots correspond to stable nodes, saddles, stable foci, and unstable foci, respectively. For instance, when *m * = * *0 and *x*_0_ = −2.6, a stable node exists and LC does not exist, whence trajectories exhibit a normal state in Figure 45, *E* and *E1*. We discuss all solutions found in Figure 45 in the sections: “Monostability in the fast-slow subsystem” and “Coexistence in the fast-slow subsystem”.

#### Periodic switch between a DB and a NS in the fast-slow subsystem

**Figure 48.**
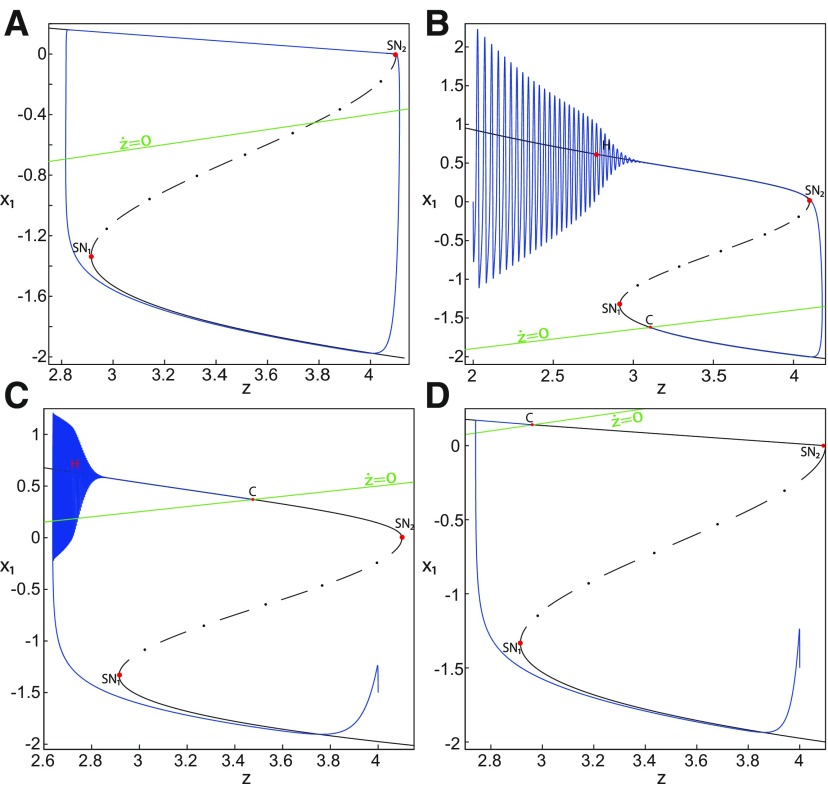
Bifurcation diagram of fast-slow subsystem with respect to z, as *m* and *x*_0_ vary. ***A***, *m* = –8, *x*_0_ = −1.4, the fast-slow subsystem exhibits a periodic switch between DB and NS. ***B***, $m=0,x_{0}=-2.4$, the fast-slow subsystem stabilizes its stable node which is the equilibrium point of NS. ***C***, $m=0,x_{0}=-0.5$, the fast-slow subsystem stabilizes its stable focus, which is the equilibrium point of nonoscillatory state. ***D***, $m=-8,x_{0}=-0.6$, the fast-slow subsystem stabilizes its stable node, which is the equilibrium point of DB. *r * = * *0.0005, I.C = [0 −5 2.817], and *T_s_* = [0:0.01: 3000] for ***A***; *r * = * *0.001, I.C = [0 −5 2], and *T_s_* = [0:0.01: 2000] for ***B***; *r * = * *0.001, I.C = [−1.5 −5 4], and *T_s_* = [0:0.01: 3500] for ***C***; and *r * = * *0.0005, I.C = [−1.5 −5 4], and *T_s_* = [0:0.01: 5000] for ***D***.

**Figure 49.**
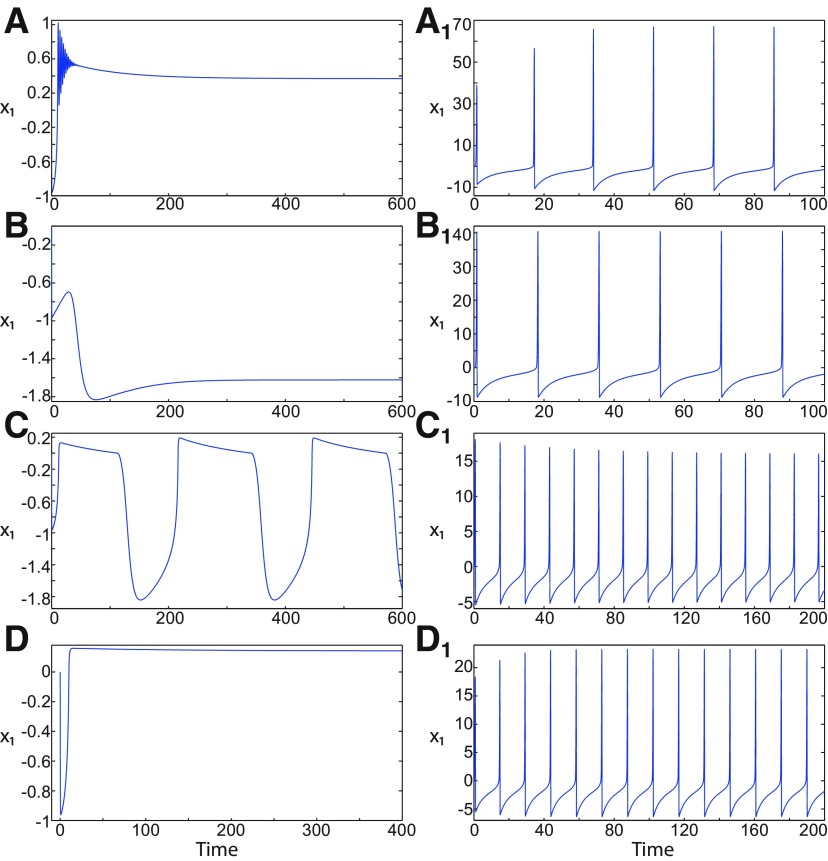
Time series of the fast-slow subsystem as *m* and *x*_0_ vary. Initial conditions are [0 −5 3] for left column and [0 −5 −1] for right column. ***A***, ***A_1_***, *m * = * *0, *x*_0_ = −0.5, the fast-slow subsystem remains in the nonoscillatory state (***A***), which coexists with LC (***A_1_***). ***B***, ***B_1_***, *m * = * *0, *x*_0_ = −2.4, the fast-slow subsystem remains in the NS (***B***), which coexists with LC (***B_1_***). ***C***, ***C_1_***, *m* = −8, *x*_0_ = −1.4, the fast-slow subsystem switches between DB and NS (***C***), coexisting with LC (***C_1_***). ***D***, ***D_1_***, *m* = −8, *x*_0_ = −0.6, the fast-slow subsystem remains in DB (***D***), which coexists with LC (***D_1_***). *r * = * *0.001 (***A***, ***A_1_***), *r * = * *0.005 (***B***), *r * = * *0.001 (***B_1_***), *r * = * *0.005 (***C***), *r * = * *0.001 (***C_1_***), *r * = * *0.001 for (***D***), and *r * = * *0.01 (***D_1_***).

When *m * ≤* * 1, a Hopf bifurcation *H* partitions the *Z*-upper branch into two sub-branches: one consists of an unstable focus (∀z<z(H)) and another consists of a stable focus (∀z>z(H); Fig. 41*A*). When decreasing *m*, the imaginary part of the complex-conjugate eigenvalues corresponding to the stable focus goes to zero, and then the stable focus becomes a stable node. We plot a (*z*, *x*_1_) bifurcation diagram when *m* = −8 in Figure 48*A*. The *Z*-lower branch consists of stable nodes, which are the equilibrium points of the NS. The *Z*-upper branch consists of stable nodes ($z>z(SN_{1})>z(H)$) which are responsible for the silent activity, similar to a DB. The *z*-nullcline is at the *Z*-middle branch which consists of saddles (*x*_0_ = −1.6). When a trajectory is at the *Z*-lower branch, the stable node disappears as *z* decreases through a saddle-node bifurcation SN_1_ and then the trajectory enters into a depolarization block, which terminates as *z* increases in a saddle-node bifurcation, SN_2_. Thus, the transitions between the *Z*-lower and *Z*-upper branches are reduced to a periodic switch between a NS and a DB. Time series are plotted in Figure 49*C*.

#### Stabilizing equilibrium points in the fast-slow subsystem

The transitions between *Z*-lower and *Z*-upper branches occur when the equilibrium point is a saddle (*x*_0_ = −1.6). When *x*_0_ is increased, the fast-slow subsystem stabilizes the equilibrium point of the *Z*-upper branch, and when *x*_0_ is decreased, the fast-slow subsystem stabilizes the equilibrium point of the *Z*-lower branch (normal state).

##### Stabilizing the equilibrium point of the nonoscillatory state

Let *x*_0_ = −0.5. We plot a (*z*, *x*_1_) bifurcation diagram when *m * = * *0 in Figure 48*C*. The *z*-nullcline is at the *Z*-upper branch which consists of a stable focus. Decreasing z, the stable node disappears through a saddle-node bifurcation SN_1_ and the fast-slow subsystem switches to the *Z*-upper branch (dashed) exhibiting an oscillatory solution, which terminates in a Hopf bifurcation, H. The fast-slow subsystem exhibits a nonoscillatory solution at the *Z*-upper branch (solid) and continues to *C*, at which *z* stabilizes. The imaginary part of the eigenvalues of *C* is responsible for the oscillations around the stable focus, which is the equilibrium point of the nonoscillatory state. Time series are plotted in Figure 49*A* for the nonoscillatory state.

##### Stabilizing the equilibrium point of the DB

Let *x*_0_ = −0.5. We plot a (*z*, *x*_1_) bifurcation diagram when *m* = −8 in Figure 48*D* The *z*-nullcline is at the *Z*-upper branch which consists of stable nodes. Decreasing *z*, the stable node disappears through a saddle-node bifurcation, SN_1_, then the fast-slow subsystem switches to the *Z*-upper branch (solid) exhibiting DB and continues to *C*, at which *z* stabilizes. The imaginary part of the eigenvalues of *C* goes to zero, and therefore the oscillations around *C* disappear. Here, the stable node (*C*) is the equilibrium point of DB. Time series are plotted in Figure 49*D* for the DB.

##### Stabilizing the equilibrium point of the NS

Let *x*_0_ = −2.4. We plot a (*z*, *x*_1_) bifurcation diagram when *m * = * *0 in Figure 48*B*. The *z*-nullcline is at the *Z*-lower branch which consists of stable nodes. When a trajectory is at the *Z*-upper branch (dashed), it exhibits an oscillatory solution, which terminates as *z* increases in a Hopf bifurcation, *H*, and then a nonoscillatory solution on the *Z*-upper branch (solid), which terminates as *z* increases in a saddle-node bifurcation SN_2_. The fast-slow subsystem continues to *C*, at which *z* stabilizes. The stable node (*C*) is the equilibrium point of the NS. Time series are plotted in Figure 49*B* for the NS.

#### Monostability in the fast-slow subsystem

Figure 45 shows that the fast-slow subsystem presents a monostability in two cases:

*Case 1:* Only LC exists. $<\dot{z}>$ has a simple zero for *m * = * *2 and *x*_0_ = −1.6, which is LC (Fig. 47*A*,*b*). Then only LC exists, to which all trajectories converge (Fig. 45,*B* and *B_1_*).

*Case 2:* Only normal state exists. The *z*-nullcline and $<x_{1}>$-curve do not intersect when *m * = * *0 and *x*_0_ = −2.6 (Fig. 46*D*). Then LC disappears through an SNPO bifurcation (Fig. 47*B*,*a*). As a consequence, only a stable node exists to which all trajectories converge (Fig. 45,*E* and *E_1_*).

#### Coexistence in the fast-slow subsystem

Figure 45 shows that LC coexists with SLE or SLC_1_. Using bifurcation analysis, we identify all coexisting attractors in the fast-slow subsystem.

##### Coexistence of LC and SLE

A coexistence occurs when $<\dot{z}>$ has two zeros, which are LC and S. LC coexists with SLE, which occurs through a fold/Hopf bifurcation (Fig. 45,*F* and *F1*) or a fold/homoclinic bifurcation (Fig. 45,*D* and *D1*). The equilibrium point is a saddle. A saddle periodic orbit (*S*) acts as a separatrix between LC and SLE (Fig. 47*B*).

##### Coexistence of LC and SLC_1_

$<\dot{z}>$ has three zeros, which are LC, S, and SLC_1_ (Fig. 47*A*,*b*). LC coexists with a periodic solution (SLC_1_; Fig. 45*C* and *C_1_*). The equilibrium point is a saddle. A saddle periodic orbit (S) acts as a separatrix between LC and SLC_1_ (Fig. 47*B*,*b*).

##### Coexistence of LC and a NS

Figure 47*B* shows the coexistence of LC and a stable node (blue dots), which is the equilibrium point of the normal state. Therefore, LC and the normal state coexist and are separated by S. Let *m * = * *0 and *x*_0_ = −2.4, time series are plotted in Figure 49*D* for the normal state and in Figure 49*D*_1_ for LC. The bifurcation diagram is plotted in Figure 48*B*. Only the normal state is shown.

##### Coexistence of LC and a nonoscillatory state

(Figure 47*B*,*a*), shows the coexistence of LC and a stable focus (green dots), which is the equilibrium point of the nonoscillatory state. Then, LC and the nonoscillatory state coexist and are separated by S. Time series are plotted in Figure 49*A* for the nonoscillatory state and in Figure 49*A*_1_ for LC. The bifurcation diagram is plotted in Figure 48*C*. Only the nonoscillatory state is shown.

##### Coexistence of LC and a periodic switch between nonoscillatory state and NS

When *m* is decreased and the equilibrium point is a saddle, the fast-slow subsystem switches between the nonoscillatory state and the NS through a fold/fold bifurcation (Fig. 45*A*). (Figure 47*B*,*a*), shows the coexistence of LC and a saddle (black dots), which is the equilibrium point of this periodic switch. Therefore, LC and the periodic switch between nonoscillatory state and NS coexist and are separated by S. Time series are plotted in Figure 45*A* for the periodic switch between nonoscillatory state and NS, and in Figure 45*A*_1_ for LC.

##### Coexistence of LC and a periodic switch between DB and NS

When *m* is further decreased and the equilibrium point is a saddle, the fast-slow subsystem switches between DB and NS (Fig. 48*A*). Time series are plotted in Figure 49*C* for the periodic switch between DB and NS, and in Figure 49*C1* for LC. Parameters *m* and *x*_0_ are the same, only initial conditions change. Then, LC and the periodic switch between DB and NS coexist.

##### Coexistence of LC and a DB

When *m* is decreased, the fast-slow subsystem enters into DB (Fig. 49*C*). When *x*_0_ is increased, the fast-slow subsystem remains in DB (Fig. 48*D*). Time series are plotted in Figure 49*D* for DB, and in Figure 49*D*_1_ for LC. Hence, LC and DB coexist.

#### Parameter space of equilibrium points and periodic orbits

**Figure 50.**
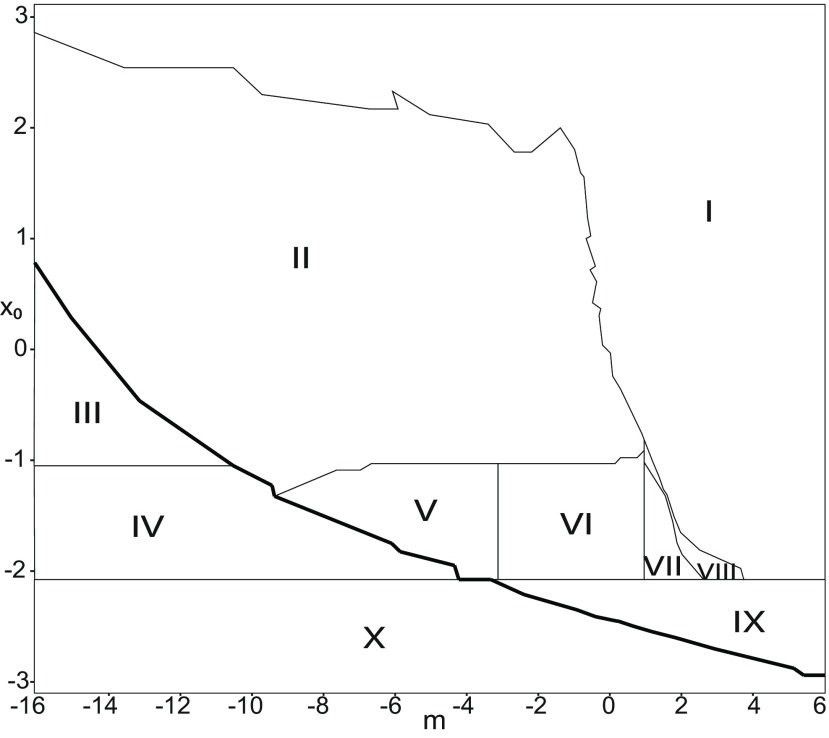
Parameter space of the fast-slow subsystem with respect to the parameters *m* and *x*_0_. There are 10 areas separated by a boundary (bold line), above which LC exists, and below it does not. For large values of *m* and *x*_0_ (area *I*) only LC exists. The adjacent area II shows the bistability of LC and a stable focus. Both attractors are separated by a saddle periodic orbit. In the middle, LC coexists with the SLE attractor separated by a saddle periodic orbit. SLE occurs via a saddle-node/saddle-node bifurcation (area VI) and a saddle-node/homoclinic bifurcation (area VII). In area VIII, the HB is not completed and gives rise to another (coexisting) stable periodic orbit with a small amplitude. In area V, DB coexists with LC. In area IX, normal brain activity and LC coexist. In area X, only normal activity exists.

To characterize the coexisting attractors, we explore a (*m*, *x*_0_) parameter space of the fast-slow subsystem in Figure 50, using numerical techniques. The parameter space is divided in two parts separated by a bold line (SNPO bifurcation), above which LC exists, while it does exist below it. There are 10 areas. For large values of *m* and *x*_0_ (area I) only LC exists. The adjacent area II shows bistability of LC and a stable focus. Both attractors are separated by a saddle periodic orbit. In the middle, LC coexists with the SLE attractor separated by a saddle periodic orbit. SLE occurs via a saddle-node/saddle-node bifurcation (area VI) and a saddle-node/homoclinic bifurcation (area VII). In area VIII, the homoclinic bifurcation HB is not completed and gives rise to another (coexisting) stable periodic orbit with a small amplitude, SLC_1_. In area V, DB coexists with LC. In area IX, normal brain activity and LC coexist. In area X, only normal activity exists.

*(I) LC:* The *z*-nullcline intersects the $<x_{1}>$-curve at one point which is LC, and intersects the (*z*, *x*_1_) curve at different equilibrium points. The equilibrium point can be a stable focus, an unstable focus, or a saddle. All trajectories converge to LC. Time series are plotted in Figure 45, *b* and *b_1_*, for LC.

*(II) Coexistence of LC and a stable focus:* The *z*-nullcline intersects the (*z*, *x*_1_) curve at one equilibrium point, which is a stable focus, and intersects the $<x_{1}>$-curve at two points: LC and S. The trajectories converge to LC or a stable focus, depending on the initial conditions. A saddle periodic orbit (S) limits the basin of attraction of LC and the basin of attraction of a stable focus. The stable focus is the equilibrium point of the nonoscillatory state. Then, LC and nonoscillatory state coexist and are separated by S.

If a trajectory is in the basin of attraction of the stable focus, then it exhibits a nonoscillatory solution following two scenarios. When the trajectory is at the *Z*-lower branch, the stable node disappears through a saddle-node bifurcation SN_1_ and the trajectory switches to the *Z*-upper branch, which comprises a Hopf bifurcation point, *H*. If $z(H)>z(SN_{1})$ (first scenario), then the trajectory exhibits an oscillatory solution, which terminates as *z* decreases in a Hopf bifurcation, *H*. Then, the trajectory continues to the stable focus at which *z* stabilizes. If $z(H)<z(SN_{1})$ (second scenario), then the trajectory exhibits a nonoscillatory solution, which continues as *z* decreases to the stable focus at which *z* stabilizes. The stable focus is the equilibrium point of the nonoscillatory state. The system is bistable on [SN_1_, SN_2_].

Using analytic techniques, we observe that the stable focus reduces as *m* decreases to a stable node (i.e., the imaginary part of the complex-conjugate eigenvalues corresponding to the stable focus goes to zero). Here, the stable node is the equilibrium point of the DB, and then DB is present in area II. LC and DB coexist as *m* decreases.

*(V) Coexistence of LC and a periodic switch between DB and NS:* The *z*-nullcline intersects the (*z*, *x*_1_) curve at one equilibrium point, which is a saddle, and intersects the $<x_{1}>$-curve at two points: LC and S. A Hopf bifurcation point, *H*, exists for *m *≤* *1 (Fig. 42). Using analytic techniques (Fig. 42), we can deduce that $z(H)<z(SN_{1})$. The transitions between *Z*-upper and *Z*-lower branches occur through a fold/fold bifurcation. For small *m*, the *Z*-upper branch consists of stable nodes, which is the equilibrium point of a DB. The system is bistable on [SN_1_, SN_2_]. The transitions between *Z*-upper and *Z*-lower branches reduce to a periodic switch between DB and NS. The LC and periodic switch between DB and NS coexist and are separated by S.

*(VI) Coexistence of LC and SLE with a fold/fold bifurcation:* Using numerical techniques, there is a saddle-node bifurcation at both offset and onset seizures. We can deduce that SLE occurs with a fold/fold bifurcation in area VI. The *z*-nullcline intersects the $<x_{1}>$-curve at two points: LC and S. As a consequence, LC and SLE with a fold/fold bifurcation coexist and are separated by S. In contrast to area V, the *Z*-upper branch consists of a stable focus, which is the equilibrium point of the nonoscillatory state. The SLE attractor reduces then to a periodic switch between nonoscillatory state and NS, which occurs through a fold/fold bifurcation, coexisting with LC. Time series are plotted in Figure 45*A* for a periodic switch between nonoscillatory state and NS, and in Figure 45*A_1_* for LC.

Using analytic techniques (Fig. 42), we can deduce that as *m* increases, $z(H)>z(SN_{1})$. This means that although there is a saddle-node bifurcation at both offset and onset seizures, there is a Hopf bifurcation occurring during the ictal period before offset seizure. Thus, an SLE occurs as *m* increases through a fold/Hopf bifurcation in area VI, coexisting with LC. Time series are plotted in Figure 45*F* for SLE with a fold/Hopf bifurcation, and in Figure 45 *F1*for LC.

*(VII) Coexistence of LC and SLE with a fold/homoclinic bifurcation:* The *z*-nullcline intersects the (*z*, *x*_1_) curve at one equilibrium point, which is a saddle, and intersects the $<x_{1}>$-curve at two points: LC and S. Since *m *>* *1, then the transitions between *Z*-upper and *Z*-lower branches occur through a fold/homoclinic bifurcation. For *m * = * *1, a Hopf bifurcation point, *H*, exists such that $z(H)>z(SN_{1})$ (Fig. 42). When a trajectory is at the *Z*-upper branch, then it exhibits an oscillatory solution, which touches the *Z*-middle branch before the Hopf bifurcation, *H*. Then the transitions between *Z*-upper and *Z*-lower branches occur through a fold/homoclinic bifurcation for *m * = * *1. LC and a fold/homoclinic bifurcation coexist and are separated by S. Time series are plotted in Figure 45*D* for fold/homoclinic bifurcation and in Figure 45*D_1_* for LC.

*(VIII) Coexistence of LC and SLC_1_:* The *z*-nullcline intersects the (*z*, *x*_1_) curve at one equilibrium point, which is a saddle, and intersects the $<x_{1}>$-curve at three points: LC, SLC_1_, and S. Then LC and SLC_1_ coexist and are separated by S. If a trajectory is in the basin of attraction of SLC_1_, then it exhibits a periodic solution with a small amplitude. When the trajectory is at the *Z*-lower branch, the stable node disappears through a saddle-node bifurcation, SN_1_, and the trajectory switches to the *Z*-upper branch. Then, the trajectory exhibits, as *z* increases, an oscillatory solution, which stabilizes at SLC_1_. Time series are plotted in Figure 45*C* for SLC_1_ and in Figure 45*C_1_* for LC.

*(IX) Coexistence of LC and a NS:* The *z*-nullcline intersects the (*z*, *x*_1_) curve at one equilibrium point which is a stable node, and intersects the $<x_{1}>$-curve at two points: LC and S. The stable node is the equilibrium point of the normal state. Then, LC and the normal state coexist and are separated by S. Time series are plotted in Figure 49*B*) for the normal state and in Figure 49*B_1_* for LC. The fast-slow subsystem switches to NS according to three scenarios, depending on *m*. We discuss these scenarios below in area X.

In the following areas, LC and S disappear through a SNPO bifurcation.

*(III) DB:* The *z*-nullcline intersects the (*z*, *x*_1_) curve at one equilibrium point, which is a stable node (Fig. 43*A*). The range of *m* indicates that a Hopf bifurcation point *H* exists, with $z(H)<z(SN_{1})$ (Fig. 42). When a trajectory is at the *Z*-lower branch, the stable node disappears through a saddle-node bifurcation, SN_1_, and the trajectory switches to the *Z*-upper branch exhibiting DB and continues to the equilibrium point, at which *z* stabilizes. Therefore, the fast-slow subsystem remains in the depolarization block.

*(IV) Periodic switch between DB and NS:* This area exhibits a periodic switch between DB and NS, like area V. In contrast, LC and S disappear through a SNPO bifurcation.

Therefore, the fast-slow subsystem only switches between DB and NS.

*(X) NS:* The *z*-nullcline intersects the (*z*, *x*_1_) curve at one equilibrium point, which is a stable node. Time series of this solution are plotted in Figure 45, *E* and *E1*. Trajectories switch to the normal state according to three scenarios, depending on *m*.

- For *m* < 1 (first scenario), a Hopf bifurcation point, *H*, exists. If a trajectory is at the *Z*-upper sub-branch, which consists of unstable foci, it exhibits an oscillatory solution that terminates as *z* increases in a Hopf bifurcation, *H*. Then, the trajectory exhibits a nonoscillatory solution on the *Z*-upper sub-branch, which consists of stable foci. The nonoscillatory solution terminates as *z* decreases in a saddle-node bifurcation SN_2_ and the trajectory switches to the *Z*-lower branch.
- For *m* = 1 (second scenario), a Hopf bifurcation point *H* exists. If a trajectory is at the *Z*-upper sub-branch, which consists of unstable foci, it exhibits an oscillatory solution, which touches the *Z*-middle branch before the Hopf bifurcation, *H*, as *z* increases. Then the trajectory switches to the *Z*-lower branch through an HB.
- For *m* > 1 (third scenario), a Hopf bifurcation point, *H*, does not exist. If a trajectory is at the *Z*-upper branch, which consists of unstable foci, then it exhibits an oscillatory solution, which terminates as *z* increases in a homoclinic bifurcation, and the trajectory switches to the *Z*-lower branch.

The *Z*-lower branch consists of stable nodes, which are the equilibrium points of the normal state. At the *Z*-lower branch, the trajectory continues to a stable node, at which *z* stabilizes. Therefore, the fast-slow subsystem only remains in the normal state. Time series are plotted in Figure 45, *E* and *E1*, for the normal state.

Note that the SNPO curve (bold line) decreases as *m* increases.

#### Special cases

The fast-slow subsystem has one equilibrium point, which is a stable focus, an unstable focus, a saddle, or a stable node, depending on *m* and *x*_0_. When LC coexists with an attractor and both are separated by S, the trajectories converge to LC or the attractor, depending on initial conditions. When S does not exist (*x*_0_ = −1.6; Fig. 47*B*,*b*), then all trajectories converge to LC (Fig. 45*B*,*B1*).

**Figure 51.**
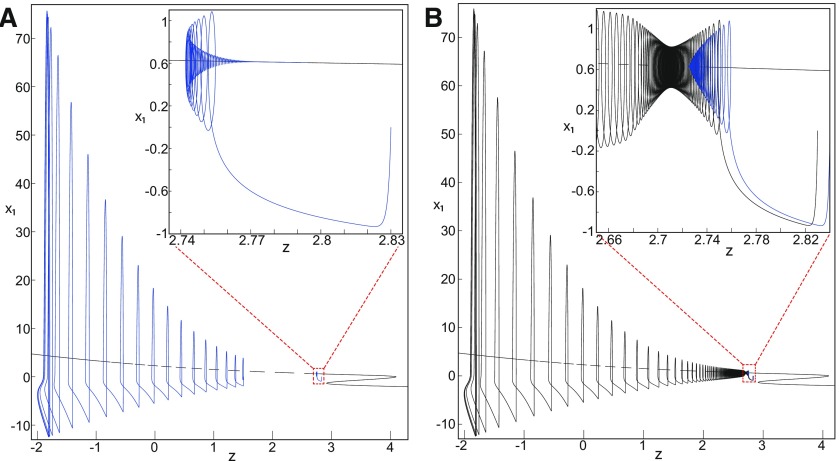
Special cases. ***A***, ***B***, Coexistence of LC and a stable focus even if a saddle periodic orbit does not exist. ***A***, *m * = * *0, *x*_0_ = −0.1. Parameters are as follows: *r * = * *0.002, I.C = [0 −5 2.83] on the right; I.C = [0 −5 1.5] on the left, and *T_s_* = [0:0.01:600]. ***B***, *m * = * *0, *x*_0_ = −0.05. Parameters are as follows: *r * = * *0.002, black trajectory: I.C = [0 −5 2.83] and *T_s_* = [0:0.01:600]; Blue trajectory: I.C = [0 −5 2.84] and *T_s_* = [0:0.01:900].

Figure 47*B*,*a*, shows that a stable focus and LC coexist, and S does not for *x*_0_ = −0.1 and *x*_0_ = −0.05. We plot trajectories in a (*z*, *x*_1_) bifurcation diagram for *x*_0_ = −0.1 (Fig. 51*A*) and *x*_0_ = −0.05 (Fig. 51*B*). Even if S does not exist, trajectories converge to either LC or a stable focus, depending on the initial conditions.

### Subsystem 2

Here we present results of our analysis on the dynamics of subsystem 2 without coupling. We partition the results into two main parts: the equilibrium points and their stability, and bifurcations.

#### Subsystem 2 equilibrium points

We analytically determine the equilibrium points (*x*_2_, *y*_2_) by solving the following equations:

(67) $$x_{2}-x_{2}^{3}-f_{2}(x_{2})+I_{ext_{2}}=0$$

and

(68) $$y_{2}=f_{2}(x_{2}).$$

We determine the stability of equilibrium points by evaluating the eigenvalues of the Jacobian matrix, *J*_2_, as follows:

$$J_{2}=\left|\begin{array}{cc} 1-3x_{2}^{2} & -1\\ G_{2} & \frac{-1}{\tau_{2}} \end{array}\right|$$

where

(69) $$G_{2}=\{\begin{array}{ll} 0 & \;\text{if}\;x_{2}<-0.25\\ \frac{a_{2}}{\tau_{2}} & \;\text{if}\;x_{2}\geq -0.25. \end{array}$$

The trace $Tr(J_{2})$ and the determinant $Det(J_{2})$ of *J*_2_ are given by:

(70) $$Tr(J_{2})=1-3x_{2}^{2}-\frac{1}{\tau_{2}}$$

(71) $$Det(J_{2})=G_{2}-(1-3x_{2}^{2})/\tau_{2}.$$

The roots of $Tr(J_{2})$ are $\forall \tau_{2}\geq 1$:

(72) $$\{\begin{array}{l} x_{2}(Tr_{1})=-\frac{1}{\sqrt{3}}\sqrt{1-\frac{1}{\tau_{2}}}\\ x_{2}(Tr_{2})=\frac{1}{\sqrt{3}}\sqrt{1-\frac{1}{\tau_{2}}}. \end{array}$$

The roots of $Det(J_{2})$ are $\forall a_{2}>1$:

(73) $$x_{2}(Det)=-\frac{1}{\sqrt{3}}.$$

We find the stability of equilibrium points in Table 6.

We graphically determine the equilibrium points (*x*_2_, *y*_2_) by using the *x*_2_- and *y*_2_-nullclines, where the *x*_2_-nullcline corresponds to a cubic curve given by:

(74) $$y_{2}(x_{2})=x_{2}-x_{2}^{3}+I_{ext_{2}}\;$$

and the *y*_2_-nullcline corresponds to two straight lines given by:

(75) $$y_{2}(x_{2})=\{\begin{array}{ll} 0 & \;\text{if}\;x_{2}<-0.25\\ a_{2}(x_{2}+0.25) & \;\text{if}\;x_{2}\geq -0.25. \end{array}$$

**Figure 52.**
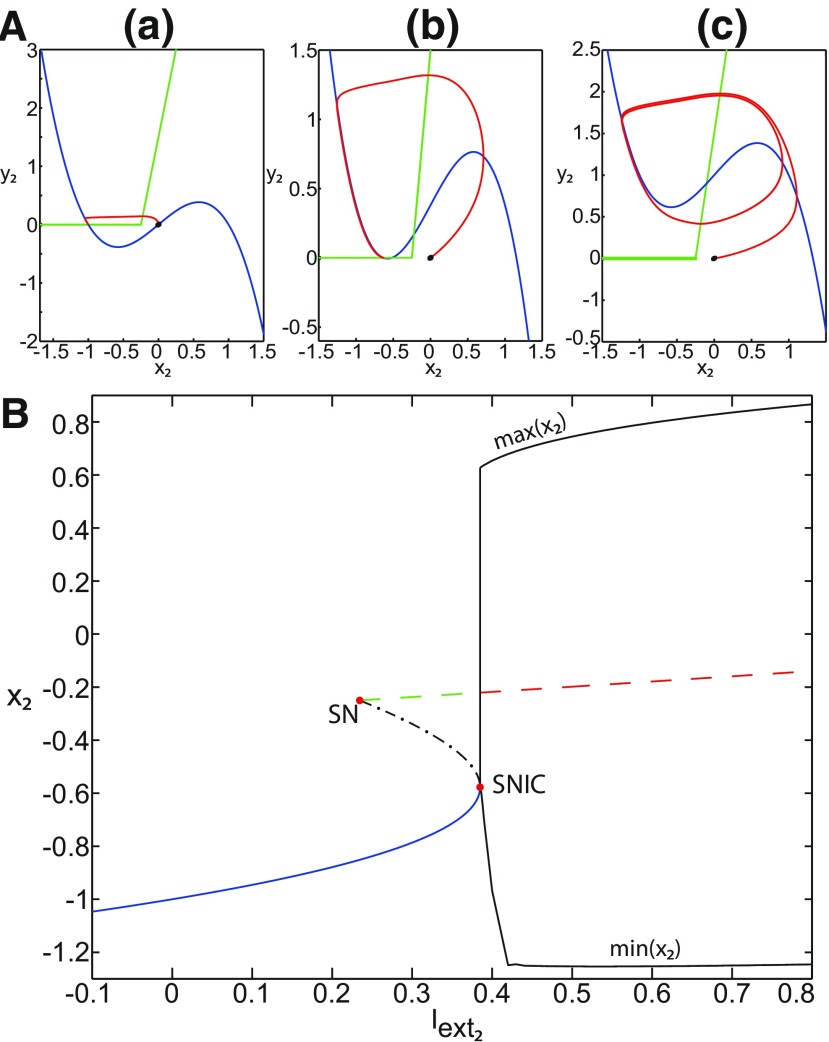
Subsystem 2 dynamics. ***A***, The (*x*_2_, *y*_2_) phase plane of the subsystem 2. Possible intersections of *x*_2_- (cubic curve) and *y*_2_-nullclines depending on $I_{ext_{2}}$. ***a***, $I_{ext_{2}}=0$, one equilibrium point (stable node) exists, to which trajectory converges. ***b***, $I_{ext_{2}}=0.38$, three equilibrium points coexist: a stable node, a saddle, and an unstable focus. Trajectory converges to the stable node. ***c***, $I_{ext_{2}}=1$, one equilibrium point exists that is an unstable focus. Trajectory converges to a stable limit cycle. ***B***, Bifurcation diagram of subsystem 2 with respect to $I_{ext_{2}}$. The lower (solid), middle (dash-dotted), and upper (dashed) branches of the S-shaped curve consist of stable nodes, saddles, and unstable foci, respectively. A branch of limit cycles (red) originates at a SNIC bifurcation. The curves above and below correspond to the maximum and minimum values along periodic orbits, respectively.

We plot a phase plane of the subsystem 2 in Figure 52, for $I_{ext_{2}}=0$ (Figure 52*A*,*a*); $I_{ext_{2}}=0.38$ (Figure 52*A*,*b*); and $I_{ext_{2}}=1$ (Figure 52*A*,*c*).

Let $I_{ext_{2}}=0$, the *x*_2_- and *y*_2_-nullclines collide in one point, which is a stable node (Fig. 52*A*,*a*). Increasing $I_{ext_{2}}$, the *x*_2_- and *y*_2_-nullclines collide in three points: a stable node, a saddle, and an unstable focus (Fig. 52*A*,*b*). The stable node and saddle approach each other as $I_{ext_{2}}$ increases and coalesce at $I_{ext_{2}}\approx 0.385$ forming an invariant circle (figure not shown). When $I_{ext_{2}}=1$, the stable node and saddle disappear, and the invariant circle becomes a limit cycle through a SNIC bifurcation. Then, trajectories converge to the stable limit cycle (Fig. 52*A*,*c*).

We analytically determine the SNIC bifurcation point by solving the following equation:

(76) $$I_{ext_{2}}=f_{2}(x_{2})+x_{2}-x_{2}^{3}$$

where *x*_2_ is a solution of

(77) $$Det(J_{2})=0.$$

#### Subsystem 2 bifurcation diagram

The dynamics of subsystem 2 changes as $I_{ext_{2}}$ varies (Fig. 52*A*). We plot a ($I_{ext_{2}}$, *x*_2_) bifurcation diagram of the subsystem 2 in Figure 52*B*. $I_{ext_{2}}$ is a control parameter. The ($I_{ext_{2}}$, *x*_2_) curve is an *S*-shaped curve, which comprises four branches. *S*-lower and *S*-upper branches consist of stable nodes and unstable foci, respectively. The *S*-middle branch consists of saddles and acts as a separatrix between the *S*-lower and *S*-upper branches. The *S*-upper branch is divided into two sub-branches: a stable limit cycle surrounds one sub-branch (red) but does not surround the other sub-branch (green). We call the red sub-branch a limit cycle branch, which is limited by $max(x_{2})$- and $min(x_{2})$-curves. Increasing $I_{ext_{2}}$, *S*-middle and *S*-upper branches collide in a saddle-node bifurcation, SN. Increasing further $I_{ext_{2}}$, *S*-middle and *S*-lower branches collide in a SNIC bifurcation ($I_{ext_{2}}\approx 0.385$). The limit cycle branch terminates as $I_{ext_{2}}$ decreases in a SNIC bifurcation.

We identify three intervals on which the equilibrium points change:

- ]-*∞*, SN[: only a stable node exists.
- ]SN, SNIC[: a stable node, a saddle, and an unstable focus coexist. Here, the unstable focus is not surrounded by a stable limit cycle.
- ]SNIC,+*∞*[: only an unstable focus exists, which is surrounded by a stable limit cycle.

The subsystem 2 is stable in the three intervals. Since the stable limit cycle emerges after a SNIC bifurcation, then there is no bistability in the subsystem 2.

**Figure 53.**
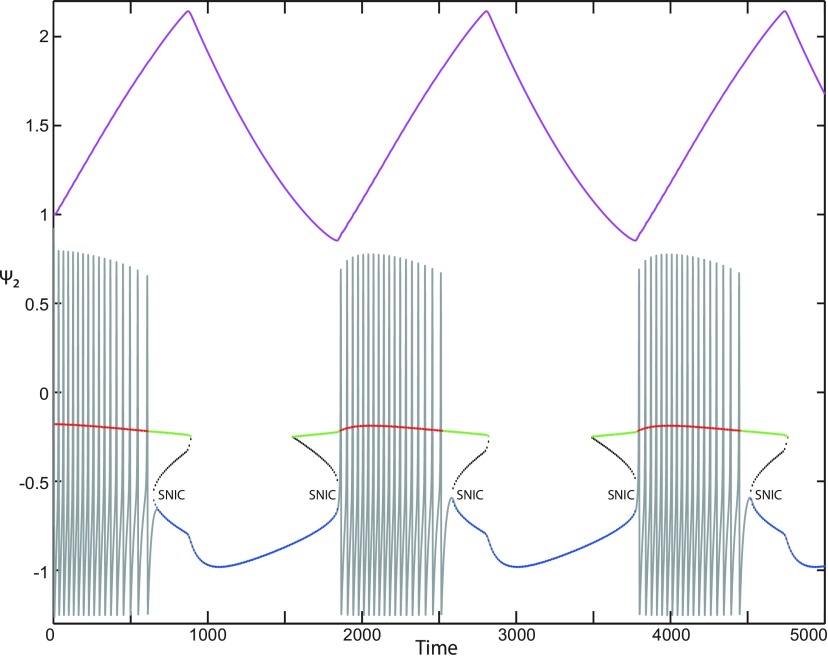
On the subsystem 2 dynamics. Time series and equilibrium points of subsystem 2 are plotted showing how the subsystem 2 evolves with time when coupled to *z* and subsystem 1, which is a periodic switch between oscillatory and resting states. On top, time series of *Z* is plotted. For easier visualization, *Z* corresponds to *z* − 2. Blue, black, and green dots correspond to stable nodes, saddles, and unstable foci, respectively. Red dots correspond to unstable foci, which are surrounded by stable limit cycles. Here all simulations were performed without noise.

We now link the dynamics of subsystem 2 to its behavior (see *ψ*_2_; Fig. 1, main file) in the Epileptor model, which corresponds to transitions between an oscillatory state and a resting state. To explain these transitions, we plot time series *ψ*_2_ and equilibrium points of the subsystem 2 in Figure 53. The equilibrium points are labeled as dots. First, we observe that the bifurcation diagram of the subsystem 2 (Fig. 52*B*) is repeated over time. Blue, black, and green dots correspond to stable nodes, saddles, and unstable foci, respectively. Red dots, which are surrounded by stable limit cycles, correspond to unstable focus. A SNIC bifurcation occurs at the intersection of blue (stable nodes) and black (saddles) dots. Second, transitions from and to the limit cycle occur through a SNIC bifurcation, and under the evolution of $I_{ext_{2}}+0.002g-0.3(z-3.5)$. In fact, the coupling function *g*(*x*_1_) and *z* change the input $I_{ext_{2}}$ of the subsystem 2 to $I_{ext_{2}}+0.002g-0.3(z-3.5)$ (see Epileptor equations). We plot time series of *z* in Figure 53. Let $I_{ext_{2}}=0.45$, the subsystem 2 is at the limit cycle branch exhibiting an oscillatory solution (Fig. 52*B*). When *z* increases, then $I_{ext_{2}}+0.002g-0.3(z-3.5)$ is decreased, and the oscillatory solution terminates in a SNIC bifurcation. Hence, the subsystem 2 exhibits a resting state. When *z* decreases, then $I_{ext_{2}}+0.002g-0.3(z-3.5)$ is increased, and the oscillatory solution emerges through a SNIC bifurcation. We conclude that the transitions between the oscillatory state and the resting state (see *ψ*_2_; Fig. 1, main file) occur under the evolution of *g*(*x*_1_) and *z*.

## Discussion

Epileptic seizures take many forms, as well as transitions from and to these pathological states. The mechanisms involved in epileptic dynamics are assumed to be different according to seizure patterns. We have used a neural mass model of partial seizures the “Epileptor” to analyze seizure dynamics. Using a mathematical approach, we showed that the model can generate different dynamic behaviors including SLEs, as the value of the parameters change. Most computational models tried to reproduce experimental data with biophysical realistic parameters (Kager et al., 2000; Traub et al., 2001; Destexhe, 2008; Cressman et al., 2009; Ullah et al., 2009). In contrast, parameters of the Epileptor model do not directly relate to biological variables. Their meaning can only be inferred based on dynamics. The SLE attractor consists of two states, an oscillatory state (limit cycle) interpreted as ictal activity, and a normal state interpreted as interictal activity. The ictal and normal states coexist and occupy two separated regions in the phase space. The SLE attractor corresponds to a periodic transition between the ictal and normal states. Transitions from ictal to normal states, and vice versa, can autonomously occur under the slow evolution of the variable *z* and without changing the parameters. The transitions from ictal to normal states occur through a bifurcation of the limit cycle, and the transitions from normal to ictal states occur through a bifurcation of the equilibrium point. The combination of two such bifurcations results in classes of (point-cycle) bursters as proposed by Izhikevich (2000). The Epileptor switches from normal to ictal states through a saddle-node bifurcation (Jirsa et al., 2014). In contrast, to return to the normal state, the Epileptor may undergo different bifurcation types, depending on the value of some selected parameters. There are two main parameters having effects on the Epileptor dynamics. The first, *m*, controls the Epileptor dynamics during the ictal period, and the second, *x*_0_, controls the equilibrium points of the Epileptor. Bifurcations that define how the Epileptor qualitatively changes its behavior were identified using the scaling behavior of frequency and amplitude during seizure onset and offset. A taxonomy of seizures (SLEs) assembles the bifurcations into 16 different classes (Jirsa et al., 2014). SLE offsets in the Epileptor show the logarithmic scaling of homoclinic bifurcations that occurred more often (83%) in drug-resistant epileptic patients As a consequence, the fold/homoclinic bifurcation was identified as the predominant class of SLEs (Jirsa et al., 2014). Through a bifurcation diagram, we showed that the Epileptor undergoes a homoclinic bifurcation at seizure offset.

On the other hand, the behavior of SLEs in the 17% of patients with a non fold/homoclinic bifurcation was potentially consistent with two classes: fold/Hopf and fold/fold cycle (Jirsa et al., 2014). We evaluated whether other bifurcations may occur at seizure offset, when changing some parameters of the model. Upon change of the first main parameter, we indeed found that the Epileptor may undergo two different types of bifurcation at seizure offset: Hopf and SNIC bifurcations. Hence, the Epileptor can generate two classes: fold/Hopf and fold/circle. Our analysis demonstrated the existence of another burster, defined by Izhikevich (2000) as a point-point type (i.e., both bifurcations are of equilibrium points), called fold/fold bifurcation. This burster does not pertain to the 16 classes (point-cycle) defined by Izhikevich (2000), and then it does not describe any behavior of SLEs.

We proposed in this study a modification of the slow *z* variable equation (Eq. 34). This modification resolves the divergences observed in the model behavior for some initial conditions, in particular when the initial *z* value is negative. More, the modification unraveled the existence of a stable LC whose behavior results from the fast-slow structure of the Epileptor model. To explore this issue theoretically, we explained in this study how the modification of the *z* variable equation leads the model to generate a fast-slow cyclic behavior, in particular for negative initial *z* values, using bifurcation analysis and the averaging method. We demonstrated that LC does not destabilize, but disappears through a SNPO bifurcation. Interestingly, LC resembles the RSE and does not exist for certain initial conditions. As a consequence, if we consider LC as a prime candidate for RSE, our analysis of the LC dynamics would contribute to the understanding of the mechanisms underlying RSE and to the prediction of how to treat RSE.

In addition to SLEs and RSE, our analysis unraveled another pathological activity, called DB. Using bifurcation analysis, we explained how the Epileptor enters into DB. Indeed, we showed that reducing the first main parameter, the frequency of oscillations during the ictal period decreases until they disappear and DB occurs. In this case, the SLE attractor reduces to a periodic switch between DB and NS. The Epileptor model can therefore reproduce SLEs, RSE, and a periodic switch between DB and NS. Interestingly, it can remain in DB if we increase the value of the second main parameter *m*, and in NS if we decrease *m*.

We predicted that epileptic seizures, refractory status epilepticus, and “normal” brain activity can coexist in the brain, under some conditions. In fact, we demonstrated that the normal and ictal states of the SLE attractor coexist in the phase space, and, for certain conditions, LC also exists below. The transitions between them require a large change of *z*. The “fold/homoclinic bifurcation” was proposed as the predominant class of SLEs (Jirsa et al., 2014). We demonstrated that the coexistence of LC and SLEs occurs for the predominant class of the SLE attractor, as well as two other classes of the SLE attractor: fold/Hopf and fold/circle bifurcations. More, we demonstrated that only the SLEs with fold/circle type can exist alone in the phase space, for certain conditions.

The Epileptor model presents another type of bistability, which consists in the coexistence of LC and a periodic switch between DB and NS. Under certain conditions, this periodic switch between DB and NS can exist in the phase space without the presence of LC. Therefore, we could deduce that DB, normal brain activity and RSE coexist in the brain. However, epileptic seizures and DB do not coexist in the brain. In fact, the SLEs and periodic switch between DB and NS do not coexist in the phase space. DB was developed during the ictal period when reducing the first parameter value *m*.

We explored the parameter space of, first, the fast-slow subsystem and extend to the whole system Epileptor. For the Epileptor, we explore two parameter spaces, as the parameter control of the subsystem 2 is varied. In one of them, DB appears, when reducing the parameter value. The parameter space can provide pathways to switch among SLEs, RSE, DB, and NS. In fact, there is a region in the parameter space representing the coexistence of LC and DB in the phase space, another where only DB exists, and a third where only LC exists. Based on these parameters, we can then propose how to switch from SLEs to DB to RSE, and how to escape the RSE and DB. Interestingly, there is a region representing the coexistence of LC and NS in the phase space, and another where only the NS exists. Therefore, we could propose how to stay in the NS, and thus how to return to the normal brain activity.

In addition to the stable limit cycle LC, the Epileptor model generates another stable periodic orbit, which we denoted by SLC. This limit cycle has higher frequency and smaller amplitude than LC, and occupies the same region as the SLE attractor. SLC is different from LC; both coexist in the phase space. The transitions between them requires a large change of the *z* variable. Furthermore, there are two different patterns of SLC depending on the equilibrium point stability of the Epileptor, which can be a saddle as for the SLE attractor, or an unstable focus. SLC mimics the dynamics of epileptic seizures during the ictal period of the SLE attractor. For the SLE attractor, the Epileptor returns to the normal state after a transient epileptic seizures, while for SLC, epileptic seizures do not stop. Hence, we believe that SLC could characterize the status epilepticus, or might have another possible clinical and physiological explanation.

In conclusion, our study provides a better understanding of the Epileptor dynamics, exploring the different behaviors that the model can generate. Three classes can be generated by the Epileptor model, which are: fold/homoclinic, fold/Hopf and fold/circle bifurcations. We predict the coexistence of epileptic seizures (SLEs), RSE, DB, and normal brain activity (NS) in the brain, under a variety of conditions. Furthermore, the model predicts the existence of different paths between these neuroelectric activities which may help to explain the mechanisms underlying their genesis, making progress in clinical and brain research.

(78) $$\{\begin{array}{ll} \alpha =\frac{R+\sqrt{\delta }}{2d} & \\ R=m+0.6{\left(z(Tr_{1})-4\right)}^{2} & \\ \delta =R^{2}+20(1-z(Tr_{1})+I_{ext_{1}}). & \end{array}$$

(79) $$\{\begin{array}{ll} \beta =\frac{R+\sqrt{\delta }}{2d} & \\ R=m+0.6{\left(z(Det_{1})-4\right)}^{2} & \\ \delta =R^{2}+20(1-z(Det_{1})+I_{ext_{1}}). & \end{array}$$

$$\beta =\frac{R+\sqrt{\delta }}{2d}\;$$

(80) $$\{\begin{array}{ll} \gamma =\frac{R+\sqrt{\delta }}{2d} & \\ R=m+0.6{\left(z(Det_{2})-4\right)}^{2} & \\ \delta =R^{2}+20(1-z(Det_{2})+I_{ext_{1}}). & \end{array}$$

## Synthesis

Reviewing Editor: Gustavo Deco, Universitat Pompeu Fabra Decisions are customarily a result of the Reviewing Editor and the peer reviewers coming together and discussing their recommendations until a consensus is reached. When revisions are invited, a fact-based synthesis statement explaining their decision and outlining what is needed to prepare a revision will be listed below. The following reviewer(s) agreed to reveal their identity: Krasimira Tsaneva-Atanasova.

Reviewer 1:

The paper content is generally fine. The structure and writing could benefit from revisions. For instance, it could be beneficial to move substantial parts (especially figures) of the manuscript to supplementary.

- Figures are not clear, circles often look like dots, there are extra (not explained in captions or main text) curves, colours and labels; it is not clear which simulations are stochastic and which are not.

- Methods are not clear enough, e.g. how were the 2D bifurcation plots computed (number of points, duration of the simulations, solvers), I’d like to see an explanation if the numerical continuation techniques were considered

- I would like to suggest including a table of all acronyms used in the paper, such as SLEs, SNIC, etc.

Some specific points:

- Page 3, Why do the methods start with a description of the paper structure?

- Page 5, I.1 Epileptor equations: What does SLE mean in this context? Seizure-like event does not make sense?

- Figure 39, please explain colour coding. There is no need to show the part of the cubic curve for x_1>0. What are i_1 to i_4

- Pages 8 and 13, the way “bifurcation curve” is used in the paper is very confusing, author’s themselves say that it is a curve of equilibria! In dynamical sytems literature, bifurcation curves are curves consisting of codimension one bifurcations and are plotted in the planes of two parameters.

- Page 12, Results, Equilibrium points: Please state explicitly what numerical techniques?

- Figure 3, It is a standard practice to show 2D bifurcation diagrams surrounded by panels with examples of dynamics. Please explain in methods how were the 2D/ parameter bifurcation diagrams computed

- Page 13, What the authors want to say by: “that equilibrium points correspond graphically to intersections of the nullclines”? Aren’t the equilibrium points, by definition, the intersection of nullclines?

- Figure 4, are the simulations in the plot stochastic? Is the <x_1> curve the average dynamics?

- Figure 11, Again it would be nice to have dynamics represented on the panels around the 2D bifurcation diagrams. Also, the tiny region 3 does not look realistically considering how coarse are the curves in the plot.

Reviewer 2:

Without having dug into all mathematical details, I have general considerations about the presentation and discussion of the results of this manuscript:

1-Abstract/Significance statement: In my opinion both the abstract and the significance statement are excessively general, devote most of the wording to talk about the landmarking features of the Epileptic model (which was already published in 2014) and lack specific references to the novelty of the paper, i.e, the new phenomena (refractory status epilepticus, RSE; depolarization block, DB) that the model is demonstrated to generate together with SLE and normal activity.

3- Introduction: I am missing more information about the connection between DP and seizures beyond the current sentence ‘DB can occur during migraine and some seizures’. Which type of seizures? Which is the neuropathological substrate of epilepsy in those cases?

3-Discussion: I suggest to summarize more the overview of the main bifurcation results and devote at least one final paragraph to discuss the potential clinical relevance of the ‘coexistence’ results and their relation, if any, to previous empirical studies on epilepsy.

Minor comments:

-page 3, line 8: ‘harness the (the)’

-page 3, line 9: what does it mean ‘resection of disconnection’?

-The first SLE abbreviation appearing in the manuscript (page 5, line 14) is not properly expanded.

-I would introduce the reference Jirsa et al, 2014 in the Discussion at the following sentence ‘We found that the Epileptor switches from normal to ictal states through a saddle-node bifurcation’ (lines 14-15 from the bottom, page 57).
